# Defining metabolic niches for marine microbial heterotrophs

**DOI:** 10.1126/sciadv.adz0537

**Published:** 2026-04-22

**Authors:** Ryan C. Reynolds, Anna C.B. Weiss, Chase C. James, Conner Y. Kojima, Jackie L. Weissman, J. Cameron Thrash, Naomi M. Levine

**Affiliations:** ^1^ Department of Marine and Environmental Biology, University of Southern California, Los Angeles, CA, USA.; ^2^ Department of Ecology and Evolution, Stony Brook University, Stony Brook, NY, USA.; ^3^ Institute for Advanced Computational Sciences, Stony Brook University, Stony Brook, NY, USA.; ^4^ Department of Biology, The City College of New York, New York, NY, USA.

## Abstract

Ocean microbial communities are made up of thousands of diverse taxa whose metabolic demands set the rates of both biomass production and degradation. Thus, these microscopic organisms play a critical role in ecosystem dynamics, global carbon cycling, and climate. While we have frameworks for relating phytoplankton diversity to rates of carbon fixation, our knowledge of how variations in heterotrophic microbial populations drive changes in carbon cycling is in its infancy. Here, we leverage global metagenomic datasets and metabolic models to identify a set of metabolic niches with distinct growth strategies. These groupings provide a simplifying framework for describing microbial communities in different oceanographic regions and for understanding how heterotrophic microbial populations function. This framework, predicated directly on metabolic capability rather than taxonomy, will enable us to tractably link heterotrophic diversity directly to biogeochemical rates in large scale ecosystem models.

## Teaser

Understanding carbon cycling requires simplifying bacterial diversity into functional groups defined by genetic signatures.

## INTRODUCTION

Classification of heterotrophic microbes into metabolic functional guilds can provide a framework for coalescing diverse microbial communities (*1*–*3*) into more tractable units for incorporation into biogeochemical models (*4*, *5*). Historically, we have grouped marine microbial heterotrophs into copiotrophic organisms, which thrive in high resource environments and generally have faster growth rates with flexible metabolisms, and oligotrophic organisms, which dominate resource poor environments and have slower growth rates (*6*). While these broad categories are useful, they do not inherently facilitate defining metabolic niches or substrate preferences that are critical when considering rates of biogeochemical cycling. Specifically, there is no intrinsic linkage between fast or slow maximum growth rates and substrate preferences for organisms (*7*). In this analysis, we expand beyond the copiotroph-oligotroph paradigm and independently assess metabolic strategy and growth rates to generate a generalizable functional categorization of marine microbial heterotrophic metabolisms.

Genome-scale metabolic models (GEMs) provide a means for translating genomic information into cellular metabolisms (*8*) but have historically been labor intensive to generate and have been generally restricted to cultured isolates (*9*). The advent of fast automated metabolic model construction software, such as CarveMe, ModelSEED, Agora (*10*–*12*), etc., has enabled the generation of GEMs for large numbers of genomes and from uncultured organisms (*13*). The metabolic potential of these genomes can further be explored using flux balance analysis (FBA) [e.g., using packages in COBRApy (*9*)]. These combined analyses provide insights into the minimal metabolic requirements for a cell and hypotheses about the preferred substrates for growth (*14*).

Here, we leveraged a large global dataset of marine microbial genomes [Ocean Microbial Database (OMD)] (*15*) to identify patterns in metabolic strategies among marine bacteria through GEMs. Testing model sensitivity to growth on different substrates allowed us to define unique clusters of marine heterotrophic bacteria with shared growth strategies. We identified a classic fast-growing copiotrophic cluster, three slow-growing oligotrophic clusters each with a unique metabolic strategy, and four intermediate-growth clusters, also with unique metabolic strategies. We demonstrate that these clusters form the building blocks of distinct microbial communities that are found in different ecological regimes.

## RESULTS

### CarveMe model quality

**Fig. 1.**
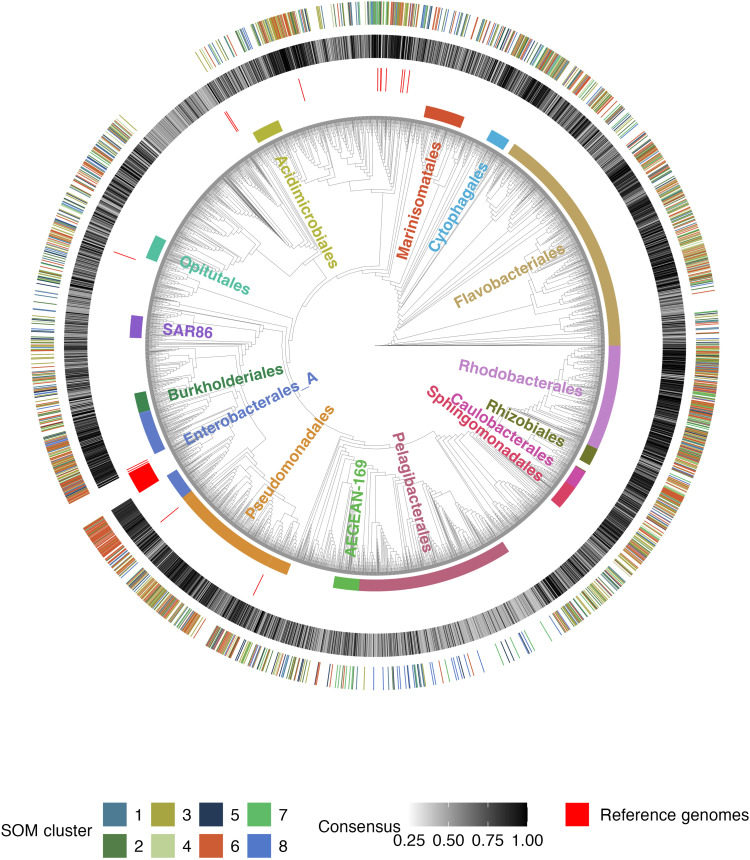
Diversity of dataset, quality of metabolic models, and designation of metabolic clusters. Phylogenetic tree of all 3738 heterotrophic bacterial genomes included in this study (including the 66 reference genomes from the BiGG database). The tree is contextualized by several external rings that describe different qualitative and quantitative components of the genomes in this study. The innermost ring shows the location of the top 15 most abundant orders. The second sparse ring of red lines denotes the position of the 66 BiGG reference genomes present in the tree. The third ring shows the ensemble consensus score (see the “Model generation and quality assessment” section in Materials and Methods and Eq. 1) for each genome in the tree. Last, the outermost ring denotes both the position of high-quality ensembles within the tree as well as the assignment of these genomes to each of our eight SOM clusters.

We generated GEMs for 3738 heterotrophic bacterial genomes from OMD (>80% completeness, <5% contamination) using CarveMe (*10*). This dataset spanned a wide diversity of marine bacteria representing 220 distinct taxonomic orders, 14 of which had 50 or more genomes (Fig. 1). To build the highest quality models possible, we chose to run CarveMe in ensemble mode (*10*, *16*), which takes into account uncertainty caused by missing annotation information and generates a user-prescribed set of *N* independent models from the same input genome (see the “Model generation and quality assessment” section in Materials and Methods). We generated large ensembles for each genome (*N* = 60 models) and assessed the robustness of the models using a consensus metric. Specifically, higher confidence can be placed in models where a consistent set of enzymatic reactions are included across the entire ensemble of models generated by CarveMe for a single genome (high consensus value).

Consensus values for the OMD genomes ranged from 1 (all model reactions were the same across all ensemble members) to 0.24 (a given reaction appeared in just 24% of ensemble models on average, thus providing low confidence in the CarveMe models). Systematic differences in consensus values were seen between phylogenetic groups (Fig. 1, third ring). The *Enterobacterales*, *Rhodobacterales*, *Cytophagales*, *Sphingomonadales*, and *Pseudomonadales* had the largest number of genomes with high consensus values: On average, 65.0% of genomes from each of these orders had consensus values above 0.8 (range of 51.3 to 81.3%). Genomes that were phylogenetically similar to the reference genomes used to develop CarveMe generally had higher consensus values (Fig. 1). Several orders had a large proportion of genomes with high consensus values despite being phylogenetically distant from the reference genomes.

The *Rhodobacterales*, for example, have no reference genome but 72.3% of these genomes had a consensus value greater than or equal to 0.8. CarveMe struggled to generate high consensus ensembles for several orders. Only 10.8% of *Pelagibacterales* and 23.3% of *Marinisomatales* genomes had consensus values at or above 0.8, while 66.5 and 28.2% of genomes in these groups had consensus values at or below 0.5, respectively. This analysis suggests that adding reference genomes (experimentally validated metabolic models used to improve the CarveMe tool) in orders with low consensus scores would substantially improve the ability for CarveMe to generate high consensus ensembles. This would greatly improve our ability to robustly apply CarveMe broadly to environmental datasets (*17*). Our further analyses only used the 1578 genomes with consensus values of 0.8 or greater.

We validated the ability of the CarveMe models to capture experimentally observed substrate preferences using an extensive culture-based analysis of carbon substrate preferences for 186 marine microbes (*18*). Specifically, we built CarveMe models for the genomes in the Gralka *et al.* study (*18*) and then performed an analogous in silico experiment using FBA to test for growth on the same set of carbon sources. Overall, we achieved a 75.5% accuracy and an estimated precision of 87.4% between the models and the data from (*18*) (fig. S1). Our measured accuracy rate was significantly higher than random, which we tested by performing a bootstrap analysis of random predictions (fig. S2). We identified two types of “problematic errors,” both with low occurrences in our dataset with a combined error of 5.5%: (i) where the model predicted growth on a compound but the experimental data showed no growth (1.0%) and (ii) where the model did not contain the reaction to use a compound but the organism was able to grow on that compound (4.5%). We also compared the growth rates predicted by the model runs against the experimental data and found that compounds yielding the fastest growth rates in the experiments also produced the highest growth rates in the model (fig. S3). A full comparison between the model predictions and experimental results is provided in text S1.

### Metabolic strategy assessment

We defined metabolic strategy as the set of substrates that are preferred by an organism for growth. We assessed the metabolic strategy for each genome using a suite of sensitivity studies. Specifically, model growth was evaluated via FBA using the slim_optimize() function in the software package COBRApy under “replete” conditions (defined here as providing all potential growth substrates at maximum flux). This was then compared to growth under “limiting” conditions in which the availability of individual compound classes was reduced. Specifically, each “limiting” condition was constructed by reducing the available flux of a compound class to 50% of the amount taken up by the organism under the “replete” condition (see the “Growth sensitivity analysis” section in Materials and Methods and Eq. 2). If this 50% reduction in substrate availability resulted in a corresponding 50% decrease in modeled growth rate, we concluded that the compound class was fully limiting growth (defined as a growth sensitivity of 1). If no change in modeled growth rate was observed, we concluded that the organism was insensitive to the availability of the compound class (growth sensitivity of 0).

The highest growth sensitivity occurred under carboxylic acid limitation, with 39.6% of all models in the dataset demonstrating substantial growth sensitivity when the availability of this compound class was limited (figs. S4 and S5A).We defined “substantial” growth sensitivity as a sensitivity value of 0.8 or higher (see the “Growth sensitivity analysis” section in Materials and Methods and Eq. 2) corresponding to a growth rate decrease of at least 40% for a 50% reduction in substrate availability. Reducing the availability of amino acids or carbohydrates resulted in substantial growth sensitivity in 28.8 and 17.9% of the models, respectively. In contrast, the models were generally insensitive to the reduction of amines/amides, alcohols, or ketones/aldehydes with only 0.13, 0.2, and 0.21% of models showing substantial growth sensitivity, respectively, when limited by the substrate class. Among the top 15 most abundant taxonomic orders, we found that the most sensitive taxa to substrate limitation across all compound classes were the *Pelagibacterales* (25.8% of models showed substantial growth sensitivity to at least one compound class), *Marinisomatales* (17.5% showed substantial growth sensitivity), and *Opitutales* (16.8% showed substantial growth sensitivity) (fig. S6A). This is consistent with these groups being classically oligotrophic organisms with streamlined genomes and limited metabolic flexibility (*19*–*21*). By contrast, the classically copiotrophic groups (the *Enterobacterales*, *Rhizobiales*, and *Mycobacteriales*) showed the least growth sensitivity to substrate reduction, with only 5.4, 6.9, and 7.4% of these models showing substantial growth sensitivity across all compound classes, respectively. This indicates that the classically designated copiotrophic orders may have more flexible metabolisms where they can achieve high growth rates using many different compound classes.

### Metabolic clusters

Metabolic niches were identified based on the substrate preference profiles using self-organizing maps (SOMs). The SOM method is an unsupervised machine learning technique that reduces large, high-dimensional datasets to a topologically defined two-dimensional grid space (*22*, *23*). We conducted an additional filtering step where we excluded genomes (*N* = 100) with high cumulative variance in the predicted substrate sensitivity across models (“Growth sensitivity analysis” section in Materials and Methods and fig. S7). Our resulting input dataset consisted of 88,680 data entries (1478 genomes × 60 models), each with 11 feature columns of metabolic sensitivity values. For our analysis, each of the ensemble models were treated individually to account for uncertainty in the growth sensitivity predictions. Eight clusters were identified using *k*-means clustering on the nodes in the SOM map (“Generation of self-organized maps” section in Materials and Methods). Each genome was assigned to the cluster that had the greatest plurality of ensemble models for that genome (“Generation of self-organized maps” section in Materials and Methods and Table 1). We tested the sensitivity of our results to the SOM and clustering parameterizations as well as our data thresholding criteria, which showed strong correlation between the clusters produced under different scenarios, providing confidence in the robustness of our findings (see the “Generation of self-organized maps” section in Materials and Methods and text S2).

**Table 1.** Description of eight SOM clusters. This table provides information about each of the eight SOM clusters including the number of genomes per cluster, the growth strategy as determined by the dCUB distributions, the mean dCUB and growth sensitivity values for the cluster, the number and names of the growth limiting substrate classes, and the two most enriched taxonomic orders for the cluster.

Cluster	Genomes	Growth strategy	% Fast growers	Mean dCUB	Mean growth sensitivity	Limiting classes		Top 2 taxa
						No.	Substrate	
1	97	Slow-intermediate	42.30%	−0.117	0.222	2	Carboxylic acids, peptides	*Marinisomatales, Pelagibacterales*
2	175	Intermediate	60.60%	−0.152	0.166	1	Amino acids	*Rhodospirillales, Burkholdeirales*
3	295	Intermediate-fast	71.90%	−0.190	0.128	1	Carboxylic acids	*Sphingomonadales, Rhodospirillales*
4	108	Intermediate	67.60%	−0.150	0.119	1	Carbohydrates	*Flavobacteriales, Cytophagales*
5	68	Slow	38.20%	−0.085	0.322	2	Amino acids, peptides	*Opitutales, Pelagibacterales*
6	478	Fast	79.50%	−0.235	0.028	0	None	*Enterobacterales, Verrucomicrobiales*
7	129	Intermediate	60.50%	−0.158	0.225	1	B vitamins	*Cytophagales, Pelagibacterales*
8	128	Slow-intermediate	47.70%	−0.120	0.248	2	Carboxylic acids, amino acids	*Pelagibacterales, Marinisomatales*

**Fig. 2.**
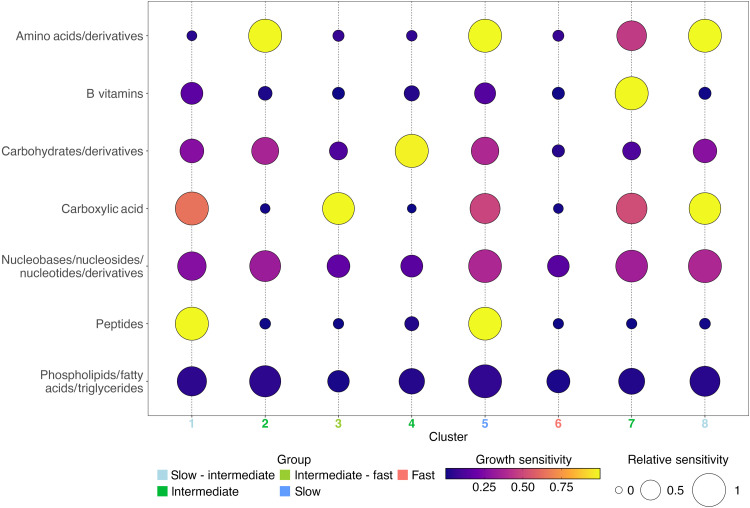
Substrate sensitivities for eight SOM clusters. Bubble plot of the mean growth sensitivity values by compound class for genomes in each of our eight SOM clusters. A growth sensitivity of 1 indicates high sensitivity to that substrate such that the modeled growth rate was reduced proportionally to the reduction in the substrate’s flux (e.g., 50% substrate reduction corresponded to 50% growth rate reduction). The size of the bubbles in this plot reflects the relative sensitivity within each compound class across the eight SOM clusters. The six compound classes that resulted in significant growth reduction for at least one of the SOM clusters are shown here. Phospholipids are shown as an example of the general pattern of sensitivity for the remaining five compound classes that did not elicit substantial growth sensitivity. The full results for all 11 compound classes are provided in table S1. Cluster numbers are colored based on genome-based estimates of maximal growth rate (fig. S8).

Differences in sensitivities to carbohydrates, carboxylic acids, amino acids, peptides, and B vitamins drove the largest separations between the clusters (Fig. 2 and fig. S5B). We observed varying degrees of substrate-driven growth sensitivities from no sensitivity to the removal of any compound class (Cluster 6) to sensitivity to the removal of a single compound class (Clusters 2, 3, 4, and 7) and to sensitivity to the removal of multiple compound classes (Clusters 1, 5, and 8). To further expand the analysis of growth strategy, we computed an estimate of maximum growth rate for each genome based on genomic optimization using codon usage bias (dCUB) (*24*, *25*). We then assessed differences in genome-based estimates of maximum growth rates between clusters and observed significant differences (pairwise Wilcoxon rank sum test with Bonferroni correction and bootstrapping analysis; see the “Statistical analyses” section in Materials and Methods and figs. S8 and S9). dCUB values were not used in the SOM clustering. We identified one cluster with significantly faster maximum growth rates (Cluster 6), three clusters with significantly slower maximum growth rates (Clusters 1, 5, and 8), and four clusters with intermediate growth rates (Clusters 2, 3, 4, and 7) (Table 1 and fig. S8). Moreover, we observed a significant relationship (adjusted *R*^2^ = 0.82) based on a simple linear regression model between the average maximum growth rate per cluster and the average growth sensitivity per cluster (fig. S10). For the remainder of the results, we will refer to three growth types: slow, intermediate, and fast (see text S3.1). For clusters with maximum predicted growth rates that are statistically in between these three types, we refer to them based on the growth types they overlap with (e.g., intermediate-fast).

The eight SOM clusters had conserved phylogenetic signals with enrichment in specific taxonomic groups (Table 1). However, we simultaneously observed that many taxonomic groups appeared across multiple clusters (fig. S6B). This suggests that diverse taxonomic groups have similar substrate preferences and growth sensitivities and also that some taxonomic groups appear to have sublineages with wide variations in lifestyle. For example, the *Enterobacterales* and *Verrucomicrobiales* were enriched in our fast-growing Cluster 6 by 194.1 and 171.8%, respectively, relative to the abundances of these groups in the total dataset (fig. S11). Similarly, the *Pelagibacterales* and *Opitutales* were on average enriched in the slow-growing Clusters 1, 5, and 8 by 346 and 247%, respectively, relative to their abundances in the total dataset. This was compared to 85.3 and 69.1% reductions of these two classically oligotrophic orders in the fast-growing Cluster 6 relative to the total dataset. However, we also found that eight of the 15 most abundant orders were present in all clusters, and only four orders were absent in more than one cluster (the *Sphingomonadales*, *Mycobacteriales*, *Rhizobiales*, and *Marinisomatales*). The *Flavobacteriales*, for instance, were found in all eight clusters accounting for between 10.2% (Cluster 8) and 35.2% (Cluster 4) of genomes (fig. S6B). Thus, although there was variation in the taxonomic composition of the clusters, the differences between clusters were not driven by taxonomy alone.

The fast-growing Cluster 6 was classically “copiotrophic” (average dCUB of −0.235). A total of 79.5% of genomes in this cluster had predicted maximum genomic growth rates that were faster (more negative dCUB) than the threshold of indistinguishably slow growth (dCUB = −0.08, where more negative dCUB values correspond to faster growth). This threshold corresponds to a doubling time of ~5 hours for mesophilic organisms (optimal growth temperature between 20° and 60°C) (fig. S8) (*25*). Taxonomically, Cluster 6 consisted primarily of the *Enterobacterales*, *Flavobacteriales*, *Rhodobacterales*, and *Pseudomonadales* (Fig. 1 and fig. S6A). Metabolically, this fast-growing cluster showed the least sensitivity to the removal of compounds with no growth sensitivity to the reduction of any of the 11 tested compound classes (Fig. 2). This suggests that these organisms have flexible metabolisms capable of growing on a wide range of substrates and are able to synthesize or substitute essential metabolites when not available from the environment. Hereafter, we will refer to this as the fast-growing generalist guild.

In contrast, the slower-growing clusters (Clusters 1, 5, and 8) had significantly slower estimated maximum growth rates than the fast-growing generalist Cluster 6 (average dCUB of −0.111) (figs. S8 and S9). The majority of genomes in these clusters (61.8% in Cluster 5, 57.7% in Cluster 1, and 52.3% in Cluster 8) had dCUB values within the “indistinguishable slow growth range” (dCUB values above the −0.08 threshold). Cluster 5 had the slowest maximum growth rates based on dCUB values and had a high proportion of known oligotrophic orders such as *Opitutales* (of phylum *Verrucomicrobiota*) and *Pelagibacterales*, with these groups enriched in this cluster by 435 and 362% relative to the overall dataset (fig. S8 and S11). All three slow-growing clusters showed substantial growth sensitivity to multiple (two or more) compound classes (Fig. 2; see the “Growth sensitivity analysis” section in Materials and Methods). For example, Cluster 8 exhibited high growth sensitivity to two classes of acids (carboxylic acids and amino acids/derivatives), suggesting that this group might be specialized for the degradation of organic acids (Fig. 2 and fig. S5B). Thus, we could think of this cluster as a slow-growing acid specialist guild.

All four intermediate-growth clusters (Clusters 2, 3, 4, and 7) had predicted growth rates that were significantly slower than the fast-growing Cluster 6 and significantly faster than slow-growing Cluster 5. One cluster (Cluster 3) also had significantly faster growth rates than the slow-growing Clusters 1 and 8 (fig. S8). All four intermediate-growth clusters showed growth sensitivity to a single compound class: amino acids, carboxylic acids, carbohydrates, or B vitamins, respectively (Fig. 2B). Overall, these intermediate-growth clusters appeared to be more flexible metabolically and faster growing than the slow-growing specialist clusters but more specialized and slower growing than the fast-growing generalist cluster. The intermediate-growth clusters corroborate a recent modeling study that suggested that the dominant heterotrophic group in the subsurface ocean might be slow-growing copiotrophs (*4*). These results support the definition of metabolic guilds for these organisms, such as an intermediate-growth, sugar-preferring guild (Cluster 4).

### Biogeographic distribution

To assess the relevance of our genomes to the marine microbial communities in situ, we quantified the relative contribution of organisms in our clusters to microbial assemblages using an amplicon sequence variant (ASV) dataset from two north-south transects across the Atlantic (GA02) and Pacific Ocean (P16N/S) (see the “Global distribution” section in Materials and Methods and text S4). Of the 1478 genomes used to generate the SOM clusters, 773 had 16*S* ribosomal RNA (rRNA) gene sequences from which we could estimate relative abundances in the ASV dataset. Despite this relatively small number of genomes, we accounted for an average of 44.3% (range of 11.9 to 73.6%) of the upper ocean community (≤150 m), using 98% sequence identity and 100% ASV sequence coverage. The fraction of the community accounted for by the high-quality models decreased significantly in the subsurface (figs. S12 to S15), suggesting that the CarveMe pipeline struggled to construct high-quality models for the heterotrophic bacteria present in the subsurface.

**Fig. 3.**
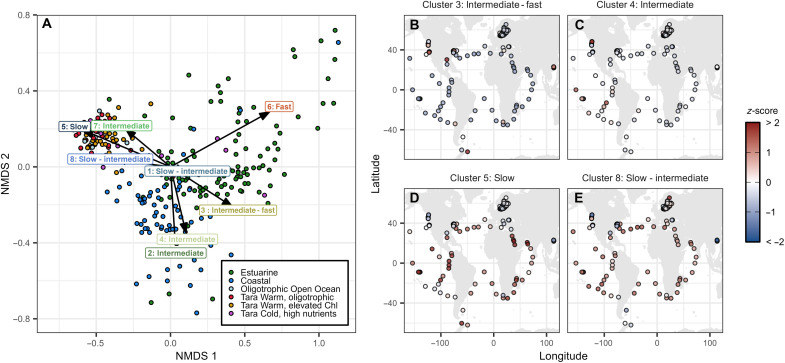
Biogeographical distribution of the SOM clusters. (**A**) NMDS plot of the relative abundances of our eight SOM clusters across six distinct ecotypes. The three ecotypes whose labels begin with “Tara” represent open ocean samples from Tara Oceans (*72*). For the subset of samples for which multiple environmental parameters were available, we further refined the ecotype using prescribing convex hulls to an NMDS of the environmental metadata (fig. S24). The data used to generate this plot are provided in data S1. (**B** to **E**) Global distribution of four example clusters (Clusters 3, 4, 5, and 8). To allow for comparison between clusters, the *z*-score normalized change in abundance is plotted (see fig. S20 for all eight maps).

To investigate the biogeographic distributions of our eight SOM clusters, we performed a competitive metagenomic recruitment and calculated normalized reads per kilobase per million mapped reads (RPKM) for 1209 globally distributed metagenomic samples. This allowed us to determine the relative abundance of each metabolic cluster based on whole genome content rather than via partial representation of our genomes in 16*S* rRNA gene ASV data and provided broader geographic coverage. We observed distinct biogeographical differences across broadly defined oceanographic categories (Fig. 3 and figs. S16 to S23). Specifically, estuarine samples were enriched in the fast-growing generalist cluster (Cluster 6) and the intermediate-fast cluster (Cluster 3), whereas the coastal samples were enriched in two of the intermediate clusters (Clusters 2 and 4). The open ocean community was enriched with the slow-growing, acid-specialist cluster (Cluster 8), the slowest-growing cluster (Clusters 5), and the intermediate-growth cluster with B-vitamin growth sensitivities (Cluster 7). The results for the open ocean samples were consistent for both the RPKM (Fig. 3) and ASV (figs. S12 and S14) analyses.

For a subset of the open ocean samples, we assessed the relationship between cluster abundance and environmental data. We observed the same general separation between metabolic strategies as was observed in the larger dataset (fig. S24) and identified three open ocean community types: high productivity communities found in colder, high-nutrient waters and characterized by higher abundances of Clusters 3 and 6 (Tara cold, high nutrients); open ocean surface communities found in warm waters with elevated chlorophyll and characterized by higher abundances of Clusters 4 and 5 (Tara warm, elevated Chl); and an oligotrophic community found in warm, low-nutrient environments characterized by higher abundances of Clusters 1, 7, and 8 (Tara warm, oligotrophic). Overall, we find that our eight SOM clusters are able to capture differences in the heterotrophic microbial community across a wide range of oceanographic regions.

## DISCUSSION

Although marine microbial heterotrophs play a primary role in regulating organic matter cycling, biogeochemical cycles, and global climate outcomes, we have historically lacked an overarching framework for characterizing these diverse communities and assessing their functional metabolic niches. Because the majority of microbial heterotrophic diversity in the oceans remains uncultured (*26*), we must rely on indirect methods to assess these metabolic strategies. We applied metabolic models to analyze the growth strategies for a large number of marine microbial genomes (the majority uncultured) via in silico methods. By combining metagenomic information, numerical models, and statistical approaches, we identified eight metabolic strategies, each with distinct substrate preferences, growth strategies, taxonomic profiles, and biogeographic distributions (Table 1 and table S1). We demonstrated that some clusters are enriched in certain taxa (e.g., *Enterobacteriales* in Cluster 6 and *Pelagibacterales* in Clusters 5 and 8) as might be expected. However, the majority of the phylogenetic groups in our dataset were distributed across multiple clusters. This suggests that organisms from diverse taxonomic groups can occupy the same metabolic niche (for this analysis, occurring in the same cluster), consistent with the finding that taxonomy and function are often decoupled (*3*) and that closely related organisms often have widely varying growth rates (*27*).

We further demonstrated that these clusters can be used to define microbial communities that differ across oceanographic regions. Our results are consistent with previous studies that have shown covariance between marine microbial communities and environmental factors, e.g., (*3*, *28*–*31*). Here, we were able to go one step further and identified overarching patterns in metabolic strategies and showed that they can be used to simplify microbial heterotrophic diversity. This step is necessary for developing formulations of dynamic marine heterotrophs for inclusion in biogeochemical models. Specifically, we must define growth rate and substrate preferences for each new functional type that is included in these coarse-grained models.

Although the clusters identified in this analysis are robust, they do not necessarily encompass all microbial heterotrophic metabolisms. In particular, we demonstrated that the CarveMe tool was not successful in creating high-quality models for the majority of genomes in many key microbial groups, such as the *Pelagibacterales*. Similarly, we showed that the subsurface community was not well represented by the genomes for which we were able to build high-quality models (text S4). We hypothesize that issues with annotation, particularly of transporter genes, as well as major differences in evolutionary history and life strategy between these marine microbes and the reference genomes for the universal model are likely drivers of poor model quality for these groups. We suggest that the development of even a handful of validated metabolic models for certain orders (e.g., the *Pelagibacterales*) will greatly improve the capacity for automated pipelines to produce high-quality models. The addition of high-quality models for poorly represented groups will allow us to investigate their metabolic strategies and potentially identify additional meaningful clusters.

This work provides avenues for future studies aimed at refining our understanding of microbial heterotrophic metabolic strategies in the ocean. We believe two high-priority areas for future work are (i) the relationship between our clusters and the flux of organic carbon compounds, e.g., (*32*), and (ii) elucidating cross-feeding dynamics and other interactions between members of the microbial community, e.g., (*33*–*35*). There are currently very few datasets that quantify microbial community composition, organic matter composition (including extracellular metabolites), and biogeochemical rate measurements. Expanding the occurrence of these types of studies is currently a priority for the community and is the goal of several international efforts including BioGeoSCAPES (*36*), Bio-GO-SHIP (*37*), and AtlantEco (*38*). Coupling such datasets with modeling approaches such as those presented in this study will provide unparalleled insight into marine microbial activity and function. In addition, metabolic models provide a means for investigating metabolite-mediated interactions between members of the microbial community. Future work focusing on validating model predictions of overflow metabolism and metabolite export will catapult these approaches from predicting the metabolic readout of individual organisms to predicting interactions between whole communities and their environment.

We demonstrated that heterotrophic microbial communities across a wide range of biogeographical regions can be described using our eight clusters as microbial building blocks, each with a distinct metabolic strategy. This provides a first step toward reducing the immense complexity of microbial heterotrophic diversity to accurately capture the impact of microbial communities on rates of biogeochemical cycling in the ocean. Our work provides a roadmap for explicitly incorporating diverse microbial communities into biogeochemical models using the growth strategies and metabolic preferences of our clusters as a set of reduced axes for parameterizing model groups. This paradigm for modeling the impact of complex heterotrophic dynamics in the global ocean promises to refine our predictions of how physical and chemical changes due to climate change will affect the biological composition of the future ocean.

## MATERIALS AND METHODS

### Genomic data

Genomic data were obtained from the OMD hosted at microbiomics.io (*15*), which contains ~35,000 microbial genomes including metagenome-assembled genomes, single amplified genomes, and cultured isolates. We included only bacterial genomes with >80% completeness and <5% contamination (*39*, *40*). These estimates were determined based on the average of the CheckM (*41*) and Anvi’o (*42*) completeness and contamination scores. Genomes were then dereplicated using dRep (*43*) with a 95% Average Nucleotide Identity (ANI) threshold, which was provided in the OMD metadata. Because we were interested specifically in examining patterns in metabolism of heterotrophic organisms, we also removed 180 photoautotrophic bacteria at the phylum level (Cyanobacteriota). We used the resulting 3738 high-quality dereplicated heterotrophic bacterial genomes as our preliminary dataset for analysis.

### Phylogeny

The phylogenetic tree of the 3918 dereplicated genomes was determined using GtoTree v1.7.0 (*44*) and IQ-TREE v2.0.3 (*45*). We chose to keep the 180 Cyanobacteriota genomes as an outgroup to help us more robustly root the tree and enforce the phylogenetic relationships of the heterotrophic genomes. We also included the 66 unique bacterial reference genomes underlying the bacterial metabolic models in the Biochemical, Genetic and Genomic (BiGG) database (*46*) that were used to generate CarveMe’s universal reaction model (*10*). The other 21 listed bacterial metabolic models in the database reflect either additional models built from the same reference genome or models that are no longer publicly available. From these 3984 total genomes, we created a multiple sequence alignment (MSA) file using the predefined bacteria single copy gene (SCG) set in GToTree v1.7.0 (*44*). During this process, eight genomes were excluded from the tree due to an insufficient number of hits to the target SCG set resulting in an alignment file of 3976 genomes. However, these eight genomes were still included in our taxonomic analyses of the SOM clusters, as we were able to assign their phylogeny using the Genome Taxonomy Database (GTDB). The MSA file was then passed to IQ-TREE v2.0.3 using the LG + R10 model with 3554 amino-acid sites to generate a phylogenetic tree (Fig. 1). For a current taxonomy of all genomes in the dataset, we overlaid full taxonomic assignments generated by GTDB-Tk v2.1.0 (*47*) with the GTDB r214 database (*48*) onto this tree.

### Model generation and quality assessment

CarveMe v1.5.1 (*10*) was used to generate multiple metabolic models for each genome, called an ensemble. The CarveMe program predicts a metabolic model for a given genome by initializing the universal model with a weight for each reaction based on its presence or absence in the annotation of the input genome. Annotated reactions receive weighting factors based on their gene-protein-reaction score, a metric that reflects the level of confidence in whether all proteins and subunits required for a reaction to take place are supported in the genome. Unannotated reactions are assigned a negative score directly by CarveMe that denotes to the solver that they are not supported with genomic evidence. These initialized reaction states are then passed to a mixed integer linear programming (MILP) algorithm, which solves for the predicted metabolism based on an objective function.

When CarveMe is initialized to predict a single metabolic model for a genome, the process is deterministic, with all reactions assigned discrete scores based on their presence or absence in the genome annotation (unannotated reactions are assigned a value of −1). When run in ensemble mode, CarveMe randomly initializes the weights of unannotated reactions between −1 and 0 for each individual model prediction, which introduces stochasticity into the model prediction process. This stochasticity better represents the unknown nature of unannotated reactions where they could be missing due to methodological issues (e.g., sequencing or annotation issues) or missing because they are truly absent. We tested a variety of ensemble sizes ranging from two models to 100 models to assess the necessary number of model replicates to effectively capture the reaction space of each genome (fig. S25). We found that the new reactions added to the total reaction space started to plateau around an ensemble size of 60. This suggests that an ensemble of 60 models is sufficient to capture the majority of possible model solutions for a given genome.

For each of the 3738 genomes in our dataset, 60 models were generated using their protein fasta files as input. CarveMe (*10*) was run using Python 3.7 (*49*) and IBM ILOG CPLEX Optimizer v20.1.0, using the native DIAMOND annotation procedure v0.9.14 (*50*). To assess the quality of the metabolic models generated by CarveMe, we developed a consensus score metric *C*. The consensus score is defined as

$$C=\frac{1}{R}\sum_{r=1}^{r=R}\frac{1}{M}\sum_{m=1}^{m=M}I\left(X_{\mathrm{mr}}=1\right)$$ (1)

where *X*_*mr*_ is the presence/absence matrix of ensemble model reactions across *M* individually generated models, *r* is an individual reaction, and *R* is the total number of reactions in the ensemble. In this context, *I* is the indicator function for the case that reaction *r* is present in ensemble model *m*. In plain terms, *C* measures the consistency of the CarveMe model reactions across the ensemble generated for a single genome. If all models in the ensemble contained all the same reactions, then the consensus score would be 1; if only half of the models had the same set of reactions, then the consensus score would be 0.5. Similar to Machado *et al.* (*10*), we equate the consensus score with overall ensemble quality because significant dissimilarities between ensemble models suggest that the optimizer in CarveMe was forced to make more uninformed choices about which reactions to keep in the model. On the other hand, an ensemble with highly consistent models suggests that the optimizer had sufficient information to consistently find the same solution (set of reactions). Only genomes with a consensus score greater than 0.8 were used for further analyses (*N* = 1578).

### Compound classification

To provide an assessment of broad metabolic strategies, we analyzed the CarveMe model growth sensitivities by compound classes. To do this, compounds that were used as substrates (imported into the cell by the model) were manually classified into the following 13 major categories: carboxylic acids, amino acids and derivatives, peptides, nucleobases/nucleosides/nucleotides and derivatives, carbohydrates and derivatives, ketones/aldehydes, organic sulfur, phospholipids/fatty acids and triglycerides, alcohols, amines and amides, B vitamins, inorganics, and “other” (table S2). We excluded inorganics and “other” categories from our downstream analyses to focus on the 11 categories with organic substrates necessary for growth. References used in the categorization included Chemical Entities of Biological Interest (ChEBI) (*51*), National Institutes of Health PubChem (*52*), BiGG Database (*53*, *54*), Human Metabolome Database (HMDB) (*55*), BioCyc (*56*, *57*), ChemSpider (*58*), Escherichia coli Metabolome Database (ECMDB) (*59*, *60*), and prior knowledge. There were 2467 external exchange reactions in the universal model representing the acquisition of compounds from the environment or media. Only 633 of the 2467 appeared as external reactions in any of our models. We conducted FBA simulations for all models and identified the compounds that were imported for growth by at least 10 models across all 94,680 CarveMe models generated for this study. These compounds (*N* = 456) were classified into the 13 major categories and were used for the growth sensitivity analyses. We also confirmed that these 456 compounds accounted for greater than 90% of the total import flux in 98.3% models across all our sensitivity tests.

### Growth sensitivity analysis

We used the FBA packages within the COBRApy v0.25.0 (*61*) software to test the CarveMe model growth sensitivities under a wide range of substrate availability. CarveMe ensemble mode generates a Systems Biology Markup Language (SBML) file, which contains a vector for each reaction in the ensemble. This vector provides the presence/absence of the reaction for each model in the ensemble. We then created a pipeline to run each FBA model simulation individually. Specifically, we extracted the reaction states from the SBML file to create a reaction matrix with the presence/absence of each reaction (rows) for each model (columns). Individual models were then run using the slim_optimize function in COBRApy using both the original ensemble SBML file and the reaction states matrix. The SBML file provided critical information about the nature of the reactions such as the reaction direction. This information is contained in the bounds for each reaction where the flux for reversible reactions are bounded between −1000 and 1000, forward reactions are bounded between 0 and 1000, and reverse reactions between −1000 and 0. We then checked the reaction states matrix to determine for the specific model (column) whether the specific reaction was present. For reactions that were not present in the model, the upper and lower bounds were set to 0, thereby excluding them from the FBA analysis. This procedure was then repeated for each model in the ensemble.

For each model in each ensemble (*N* = 94,680 total models), we assessed the type and quantity of compounds preferred for growth under replete conditions. Here, we defined replete conditions as allowing the FBA to have access to the maximum flux of all potential substrates for a given model. Specifically, we estimated the maximum model growth rate using an FBA algorithm with the slim_optimize function within the COBRApy package, which optimizes and returns the value of the biomass function with all possible media components turned on. We then determined the minimal set of compounds that allowed the previously determined maximum model growth rate using the COBRApy minimal media prediction (minimal_media function). This function solves an MILP problem to minimize the import fluxes (external exchange reactions) while maintaining the maximum model growth rate.

To assess the differential substrate requirements predicted by the models, we conducted a set of in silico tests for each model where the availability of compounds in the growth media was limited and FBA was run (hereafter referred to as compound-specific growth sensitivity tests). Specifically, for each of the 11 substrate compound classes (defined above in the “Compound classification” section in Materials and Methods), the available flux for that class was supplied at 50% of the FBA estimated import flux value in the “replete conditions” while all other substrates were allowed to reach their maximum values. Any component from the limited growth compound class that was not originally predicted as part of the minimal media of a given model (using minimal_media) was made unavailable to prevent models from circumventing the substrate limitation. We then assessed how the substrate import fluxes shifted under these limitation scenarios and the resulting change in predicted growth rate. Compound-specific growth sensitivities were computed using the following equation

$$S=\frac{1}{f}\times \left(1-\frac{\mu_{n}}{\mu }\right)$$ (2)

where μ_*n*_ is the predicted growth rate under substrate limitation by compound class *n* and μ is the predicted growth rate in the “replete conditions.” *f* is the degree of limitation applied, which is 0.5 here. The metric *S* ranges from [0, 1] where 0 means no sensitivity and 1 means complete limitation by compound class *n*. Specifically, if the substrate flux of compound class *n* was reduced by half (*f* = 0.5), then one would expect that, if substrate *n* was limiting growth, the growth rate would also drop by a factor 0.5 (*S* = 1). A model was termed to have “substantial” growth sensitivity to a specific compound class if the FBA predicted growth rate decreased at a proportion of 0.8 or greater to the amount of substrate limitation (*S* ≥ 0.8). The 11 compound-specific growth sensitivities that were estimated per model then served as the input feature data for the SOM clustering. The full enumeration of the compound specific growth sensitivities for each genome can be found in table S1.

Prior to processing the 1578 genomes through our clustering procedure, we compared the total variance in growth sensitivity across all metabolite classes to look for genomes with highly variable sensitivity predictions across the 60 models (fig. S8). Over the entire dataset of high-quality models, we observed an average cumulative variance across all metabolite classes of only 0.014. This suggests that, in general, the high-quality model ensembles generated consistent predictions of growth sensitivity to our 11 metabolite classes. We observed a small number of genomes (*N* = 100) where the cumulative variance in growth sensitivity was greater than 0.1 across all metabolite classes. Because this high variability draws into question the robustness of these growth sensitivity predictions for these models, these 100 genomes were excluded from the SOM and clustering analyses, leaving a total of 1478 genomes and 88,680 total data points.

### Validation on experimentally characterized genomes

To validate our CarveMe models and growth sensitivity analysis, we compared our model results to an experimental dataset from a study that quantified the growth of 186 heterotrophic marine microbes on 135 compounds as a sole carbon substrate (*18*). To validate our pipeline, we constructed model ensembles using the genomes for 178 microbes in the study with reported physiological data; 146 generated high-quality CarveMe model ensembles (above the consensus threshold of 0.8). Of the 135 experimentally measured compounds, we tested the 78 nonpolymeric compounds that had corresponding reactions in the BiGG database, which was necessary for them to be used in the FBA with CarveMe models. Our results exclude one of these 78 compounds, oxaloacetate, which is highly unstable and rapidly degrades to pyruvate (*62*), thus confounding the fidelity of the growth experiment with that compound as the sole carbon source.

FBA was conducted in COBRApy for each model within the ensemble using media with a single carbon substrate as done in the Gralka *et al.* study (*18*). Given that we know that the automatically generated CarveMe models are not perfect and issues with annotation can result in missing pathways from the models, we also assessed whether the models were consistently missing a key pathway(s) necessary for growth on certain compounds as sole carbon sources. Specifically, we re-ran the FBA using the sole carbon substrate media plus the addition of trace amounts of one or two additional “rescue” compounds. Here, we define trace amounts as less than 1% of the maximum allowed flux. Across the models in the ensemble, the genome was flagged as no-growth on a substrate if all of the models in the ensemble showed no growth and growth if more than 50% of the models grew on the substrate. For each genome and each substrate, we assigned one of five outcomes: (i) perfect agreement between the models and the data (either growth or no growth on sole carbon substrate); (ii) agreement (growth/no growth) between the models and the data with the addition of one or two “rescue” compounds; (iii) observed growth in the experiments and no observed growth in the models but the pathway to use the carbon substrate was present in the model; (iv) observed growth in the experiments, no observed growth in the models, and the pathway to use the carbon substrate was not present in the model; and (v) no growth in the experiments but predicted growth in the models (fig. S1).

We have also conducted a statistical analysis to provide the appropriate context for evaluating the comparison between the model and the data. Specifically, we took the original binary matrix of growth/no growth data as described in the Gralka *et al.* study (*18*) and created a random binary matrix designed to mimic the properties of the original data. To do this, we identified the number of positive growth signals (ones) present in each of the 146 genomes we assessed from the Gralka *et al.* study (*18*) and created a random matrix such that each genome (row) had that same number of ones in random positions. We then assessed the agreement between the random matrix and the observational dataset. We bootstrapped this approach with 10,000 randomly drawn matrices to determine the agreement between the model and experiments that would result by chance (fig. S2).

### Generation of self-organized maps

To identify clusters of organisms with similar metabolic strategies, we used SOMs to assess compound specific growth sensitivities. SOM is an unsupervised machine learning dimension reduction method capable of handling large data formats (*23*). SOM is a nonparametric approach, capable of highlighting nonlinear, complex patterns in two-dimensional space from highly dimensional data. The SOM map was built using the feature data from the COBRApy growth sensitivity analysis for the 88,680 high-quality ensemble models (C≥0.8) on our 11 manually defined compound classes. These scaled compound flux predictions were processed using kohonen v3.0.12 (*22*) and solved over 1500 iterations with a learning rate vector of (0.025, 0.01) and default neighborhood radii on a 20-by-20 toroidal, hexagonal grid spatially described by standard Euclidean distance. Map parameters were determined using heuristics and metrics of error proposed in the SOM literature (*23*, *63*–*66*) and are discussed further in text S2. Each node in the grid was initialized with a random codebook vector of values for each independent variable. Data entries were then randomly drawn from the dataset—every entry in the dataset was drawn in each iteration—and the grid point values of the closest neighborhood of nodes were updated.

After sufficient training, the values assigned to each grid point reflect the spatial topology of the data (e.g., density of data points and variation) as well as the full range of values in the original dataset. The final SOM map was then grouped into eight distinct clusters using *k*-means clustering (*67*) based on the coherence of the growth compound sensitivity predictions. The full map and designation of the clusters is shown in fig. S26A. After 1500 iterations, the mean object distance to its closest map unit (the quantization error) was ~1 × 10^−4^ (fig. S26B). The parameter optimization of the SOM map developed in this study is discussed in further detail in text S2.

The SOM map and clusters were built using all of the ensemble models (60 per genome). We then assigned each genome in the dataset to the SOM cluster that had a simple majority of the 60 models generated from that given genome. Of the 1478 genomes, 99.1% (1465 of the 1478) have at least 57 of their 60 models (95%) assigned to a single cluster and 71.5% of genomes had all 60 models assigned to the same SOM cluster (fig. S26C). The intracluster distances for both *k*-means and hierarchical clustering are shown in fig. S26D. Generally, intracluster distance decreases monotonically as the naive case of every data point getting its own cluster produces distance values of 0. We selected eight clusters based on the plateau in intracluster distance in the 7 to 10 cluster range as well as based on the input data.

We further tested the stability of our predicted *k*-means clusters from the SOM map nodes as well as the effect of using a different threshold for our input data on the resulting patterns we identified from the *k*-means clusters (text S3). To compare the predicted cluster assignments of our genomes between multiple independent runs of the map training and *k*-means clustering, we used the adjusted Rand index (ARI) metric (*68*), which is a [−1, 1] metric. The ARI describes how strongly correlated two independent clustering assignments are and does not require exact label matching, which is important given that the numeric labels of *k*-means clustering can shuffle randomly even for strongly conserved clusters. To capture the full variability of solutions captured by the CarveMe models, we selected individual representative models for each genome, which also allowed us to organically test the effect of using all 60 ensemble models versus one representative model. We generated 100 independent SOM maps with eight *k*-means clusters from these data subsets, as well as generating 100 random classification vectors to baseline the ARI values of truly random assignments.

### Maximum growth rate estimations

To assess differences in maximum growth rates, we estimated the dCUB for all 1478 genomes using the gRodon program (*24*). dCUB is a metric that has been empirically linked with optimization for faster growth. gRodon measures dCUB of highly expressed genes, in this case, ribosomal proteins, compared to the codon usage patterns across the whole genome. This genomic measure of maximum growth is a reasonable proxy and allows us to examine the differences in growth optimization for this set of uncultured organisms without needing to do extensive culturing and metabolic characterization efforts. Because estimating actual growth rates from dCUB requires correcting for temperature, we used the raw dCUB scores for this analysis to assess relative differences in genomic optimization for rapid growth. Previous works by Weissman *et al.* (*24*, *25*) suggest that differences in dCUB values are only reliable below the threshold of −0.08 (i.e., lower values of dCUB represent faster growth rates). We use this threshold to differentiate between “slow growth” and “fast growth” organisms. The results from these analyses are presented in text S3.2.

### Global distribution

To assess the global distribution of the genomes within each SOM cluster, we performed competitive metagenomic recruitment. Specifically, we calculated normalized RPKM with the pipeline RPKM Recruitment Analysis Pipeline (RRAP) v1.3.2 (*69*). RRAP uses bowtie2 v2.4.2 (*70*) to align reads and SAMTools v1.14 (*71*) to index and sort the read alignment data. RRAP takes read alignment statistics generated by SAMTools to calculate RPKM. A total of 1209 metagenomes were used for the read recruitment from several metagenomics surveys including Tara Oceans, BioGeoTraces, and Malaspina (*15*). The Sequence Read Archive (SRA) accession numbers and per sample read coverage estimates for the 1209 metagenomes can be found in table S3. Raw metagenome fastq files were aggregated by sample and by depth when multiple depths were present, e.g., the Tara Oceans dataset (*72*), and quality-filtered using the iu-filter-quality-minoche script from the Illumina-utils library v2.10 with default parameters. This script follows the quality filtering approach outlined in (*73*). After quality filtering, our genome set was recruited to the metagenomic reads, and RPKM values were calculated for each genome at each site.

We then partitioned our data into 22 oceanographic regions defined by Lanclos *et al.* (*74*) and aggregated the raw RPKM values for the genomes in our study (*74*). The 22 regions averaged 52.3 distinct samples per region (ranging from 2 samples/sites in the Southern Ocean to 298 samples at station ALOHA) (table S4). We then further clustered the 22 oceanographic regions into five categories: Estuarine, Coastal, Oligotrophic Seas, Oligotrophic Open Oceans, and the Southern Ocean (table S5). We used this categorization to group the sampling sites and compare the relative abundances determined from the raw RPKM values. For the sampling sites associated with each category, we clustered the relative abundances of the eight SOM clusters at each site using Bray-Curtis distance and hierarchical clustering with McQuitty linkage distance (figs. S16 and S17).

To assess the relative abundance of genomes assigned to each SOM cluster per oceanographic region or category, we conducted a bootstrap recruitment of the individual genome abundance values at each station. We used bootstrapping due to large variation both in the number of samples present in each region (ranging from 3 at SOC to 299 at ALOHA) and in the number of genomes assigned to each cluster (ranging from 68 genomes in Cluster 5 to 478 genomes in Cluster 6). For each region, we computed 1000 independent bootstrap iterations (using the fixed random seed 123), drawing 10,000 data points from the pool of RPKM samples for each of our eight clusters. During each bootstrapping step, we calculated the cumulative RPKM of the sampled data for each cluster and then compared their magnitudes to determine the relative abundances of the clusters. Average values and 95% confidence intervals were then computed from the resulting distributions of relative abundances for each cluster/region combination.

To assess the relationship between environmental data and the abundance of the SOM clusters, we conducted an Non-Metric Multidimensional Scaling (NMDS) with Bray-Curtis distance on the relative abundances of each cluster across the 84 sites from Tara Oceans for which we had environmental metadata (fig. S24). We applied an additional NMDS on the full set of parameters from the environmental data [temperature, chlorophyll-a, oxygen, nitrogen (nitrite plus nitrate), phosphate, and silicate] and defined three distinct ecotypes by calculating the convex hulls of the NMDS space using *k*-means clustering (text S4.3 and fig. S24). We defined these three ecotypes as warm, oligotrophic; warm, high chlorophyll; and cold, high nutrients based on the NMDS axes relative to each of these hulls. A handful of samples from Tara Oceans could not be assigned environmental metadata and are referred to as “Other” or “Oligotrophic Open Ocean” as this is the oceanographic regime that these stations without environmental data were assigned to. Last, we conducted an additional NMDS with Bray-Curtis distance combining the Tara Oceans metagenomic samples with the estuarine and coastal samples to identify overarching patterns of enrichments across broad oceanographic regions (Fig. 3).

In addition to our read recruitment analysis, we performed a second independent analysis of the distribution of our genomes in situ using a global ASV dataset from McNichol *et al.* (*75*). We used ASVs and the authors’ predictions of relative ASV abundances in the heterotrophic fraction of each community. We used barrnap v0.9 (*76*) with a partial match rejection threshold of 30% to identify the full set of partial/complete 16*S* rRNA sequences from our set of 1478 genomes. We identified 16*S* rRNA sequences in 773 of our 1478 genomes that we used to compare to the ASVs from two north-south transects in the Atlantic (GA02) and Pacific (P16) oceans (*75*). We then assessed the relative abundance of our genomes at each of these transects using the relative abundance data for these ASVs from McNichol *et al.* (*75*). Specifically, we used a nucleotide BLAST with BLAST v2.17.0 to map our identified 16*S* rRNA sequences to the environmental ASV dataset. Sequence alignments were thresholded stringently, only allowing alignments with percent identity greater than or equal to 98% and ASV sequence coverage of 100%. Only the top overall alignment was kept for each ASV to avoid any “cross-mapping” effect where one ASV mapped to multiple genomes. The results of these two independent estimates of biogeographic distribution and coverage are discussed in greater detail in text S4.

### Data visualization

All data visualizations in R v4.2.3 were performed using ggplot v3.4.2, ggridges v0.5.4 (*77*), ggtree (*78*), patchwork v1.1.2 (*79*), ragg v1.2.5, and plots native to kohonen v3.0.12 (*22*).

### Statistical analyses

Comparisons of predictive accuracy between CarveMe model predictions and experimental growth predictions were performed on *N* = 146 experimentally tested strains across *N* = 77 distinct substrates. Model to experiment prediction accuracy was tested against randomized model prediction accuracy using bootstrapped permutations of the experimental matrix of growth/no growth prediction. Permutation was performed by randomly assigning the same number of positive growth substrates for each of the *N* = 146 strains (e.g., a strain that was found to grow on 10 substrates would still grow on 10 random substrates). Each permuted growth matrix was compared to the original experimentally derived set of predictions, and accuracy was computed. A distribution of bootstrapped (*N* = 10,000 bootstraps) accuracy values was compared to the real model prediction accuracy by calculating whether the real prediction accuracy was greater than 2 standard deviations above the mean randomized prediction accuracy. Variations in model growth rates versus experimentally measured growth rates were determined by binning normalized model growth predictions into three groups and testing the distribution means using an analysis of variance (ANOVA) test with Tukey’s Honestly Significant Difference (HSD) and a confidence interval of 95% (or *P* = 0.05).

Variations in the distributions of growth estimates (dCUB) for the genomes associated with each of the eight SOM clusters (*N* = 68 to 478 genomes per cluster) were calculated using a nonparametric Wilcoxon rank sum test for multiple samples at a confidence interval of 95% (or *P* = 0.05) with Bonferroni-corrected *P* values. The full list of *P* values for all pairwise cluster comparisons is provided in table S6. To compare the proportion of genomes with faster growth (proportion with dCUB <−0.08) in each cluster, we performed a bootstrapping of the dCUB data using bootstrap sample sizes of *N* = 185, which reflect the mean number of genomes associated with one of the eight SOM clusters. We generated 10,000 samples of *N* = 185 genomes, computed the proportion of genomes with a dCUB below the defined threshold, and created a distribution of these proportions. The real proportions for our eight SOM clusters were plotted against this distribution and segregated into three statistical groupings based on whether the cluster proportion fell 2 standard deviations below the distribution mean, within 2 standard deviations of the mean, and 2 standard deviations above the mean. We also aggregated the mean growth sensitivity and dCUB values for each cluster and computed a simple linear regression of these two measures to assess whether sensitivity correlated with estimates of maximum growth. We computed an adjusted *R*^2^ value of 0.82 with 6 degrees of freedom between the two measures, suggesting that the two measures are strongly correlated with one another.

## Supplementary Materials

### The PDF file includes:

Supplementary Text S1 to S4 Figs. S1 to S28 Legends for tables S1 to S6 Legend for data S1 References
