# Supplementary material for: Defining metabolic niches for marine microbial heterotrophs
Source: Sci Adv. 2026 Apr 22;12(17):eadz0537. doi: 10.1126/sciadv.adz0537 (PMC13101867; doi:10.1126/sciadv.adz0537)
Supplement: Supplementary file 1 — Supplementary Text S1 to S4 Figs. S1 to S28 Legends for tables S1 to S6 Legend for data S1 References [file sciadv.adz0537_sm.pdf]

Science Advances
AAAS

Supplementary Materials for

Defining metabolic niches for marine microbial heterotrophs

Ryan C. Reynolds *et al.*

Corresponding author: Naomi M. Levine, n.levine@usc.edu

Sci. Adv. **12**, eadz0537 (2026)
DOI: 10.1126/sciadv.adz0537

The PDF file includes:

Supplementary Text S1 to S4
Figs. S1 to S28
Legends for tables S1 to S6
Legend for data S1
References

Other Supplementary Material for this manuscript includes the following:

Tables S1 to S6
Data S1

S1 CarveMe Validation

We validated the CarveMe models and predictions of growth sensitivity to specific compounds by comparing our model predictions to an extensive experimental dataset where 186 marine bacterial strains were tested for their ability to grow on 135 substrates as a sole carbon source(*18*). Of the 186 strains, 178 had reported physiological data and 146 generated CarveMe model ensembles which satisfied our 0.8 consensus threshold criteria to be considered high quality. Of the 135 tested substrates from this dataset, we identified 78 non-polymeric metabolites with corresponding BiGG annotations, which was necessary to be able to test them for growth using our model ensembles. Our results exclude one of these 78 compounds, oxaloacetate, which is highly unstable and rapidly degrades to pyruvate thus confounding the fidelity of the growth experiment with that compound as the sole carbon source. For each genome, FBA was conducted in COBRApy for each model within the ensemble using media with a single carbon substrate as done in the Gralka et al. (*18*) study. In 68.8% of cases, the CarveMe ensembles directly agreed between the model predictions and experimental results (either growth or no growth).

Given that we know the automatically generated CarveMe models are not perfect and issues with annotation can result in missing pathways from the models, we next assessed whether the models were consistently missing a key pathway(s) necessary for growth on certain compounds. Indeed, we found that adding trace amounts of one or two additional 'rescue' compounds to the model media allowed positive growth on a sole carbon substrate (Supplemental Figure S1) in an additional 6.7% of test cases. Here, we define trace amounts as less than 1% of the maximum allowed flux. To ensure that this signal was not driven simply by the ability of the models to grow on these 'rescue' compounds, we calculated the growth on just the trace amounts of the 'rescue' compounds to determine a baseline. We only counted a positive growth signal for the tested carbon substrate when growth on the sole carbon substrate in the presence of 'rescue' compounds was substantially higher than this baseline growth rate. Combining the default media and 'rescue' media results, we observed a 75.5% agreement between the models and the Gralka data.

For the instances where adding 'rescue' compounds allowed for growth, we found that a single compound nicotinamide mononucleotide (NMN) resulted in 533 more instances of agreement between the model predictions and experiments, increasing overall agreement between the modeled and experimental results from 68.8% to 73.9% (rescue media = inorganic media components + 1 tested carbon substrate + trace amounts of NMN). This suggests that specific 'rescue' compounds may be key metabolic co-factors in the universal model that were consistently excluded from the CarveMe models generated for the Gralka genomes either due to issues with annotation or due to biases in the universal model. We further observed that in 18.9% of cases the model contained the transport reaction to uptake a specific substrate but model growth was not achieved on media with the compound as a sole carbon source, even with one or two rescue compounds. This points to the fact that there are potentially additional missing cofactors or combinations of cofactors needed for growth on these compounds or missing pathways in the model.

Analyzing the model results by compound class, we found 75.0% agreement for growth/no-growth on carbohydrates, 72.0% agreement for growth/no-growth on amino acids, and 76.0% agreement for growth/no-growth on carboxylic acids. We also found that the models performed particularly poorly on lactate (56.2% agreement), butyrate (50% agreement), propionate (39.7% agreement), acetate (32.8% agreement), and pyruvate (17.8% agreement). This points to a potential bias in the CarveMe models for growth on these compounds as sole carbon sources but does not impact the primary analyses in this study where we look at the growth sensitivities to compound classes as a whole where multiple carbon substrates are supplied. Moreover, previous work suggests that many organic acids, particularly acetate and other short chain fatty acids (SCFAs) with a terminal methyl group and a single carboxyl function can become protonated and freely diffuse into the cell when present at high concentrations in the 10s of mM range(*80*). Thus, it is possible that the Gralka study, which used concentrations of 30mM, was estimating growth that resulted from a transmembrane flux of organic acids into the cell – a process not captured by the CarveMe models – rather than uptake through transporters. As a result of this potential bias, we excluded the 5 SCFAs with this particular chemical structure from our comparative analysis.

To further validate the robustness of our comparative analysis between the CarveMe model predictions and experimental data, we conducted a statistical analysis to determine the accuracy we would measure if we compared randomly generated predictions to the experimental data. Specifically, we took the original binary matrix of growth/no growth data as described in the Gralka et al. (*18*) study, and created a random binary matrix designed to mimic the properties of the original data. To do this, we identified the number of positive growth signals (ones) present across all 77 substrates in each of the 146 genomes we assessed from the Gralka study. Using this information, we created a random matrix such that each genome (row) had that same number of ones in random positions (i.e., it grew on the same number of substrates, at random). We then assessed the agreement between the random matrix and the observational dataset in the same manner as described above. We bootstrapped this approach with 10,000 randomly drawn matrices of this nature to determine the agreement between the model and experiments that would result by chance. The resulting distribution of the bootstrap analysis had a mean agreement of 61.0% with a standard deviation of 0.39%. Thus, our agreement of 75.5% overall is well above the significance threshold reinforcing our conclusion that the CarveMe models are doing a good job of capturing the Gralka results (Supplemental Figure S2). We found similar results when we permuted the growth matrix by randomly assigning the same number of positive growth signals to each strain across the N=77 substrates we compared (e.g., a substrate that 10 strains were able to grow on would still show positive growth on 10 random strains).

Finally, we compared the growth rates predicted by the model against the experimental data and found good agreement (Supplemental Figure S3). Specifically, compounds that yielded faster growth rates in the experiments also produced higher growth rates in the model (for this analysis we compare the relative growth rates by organism across the compounds tested). This relationship was determined to be statistically significant for the three binned groups of model growth rates using Tukey's HSD with a 95% confidence interval.

S2 SOM Clustering

The model growth sensitivity analysis generated 88,680 data points (1,478 genomes x 60 models each with 11 metabolic sensitivity features). To analyze this large dataset and identify overarching patterns we employed Self Organizing Maps (SOM), a type of dimensional reduction method. In addition to SOM, we analyzed the data using other traditional dimensional reduction approaches such as PCA, and clustering methods such as direct hierarchical clustering. The PCA was able to distinguish two broad clusters within the dataset with PC1 explaining 28.3% of the total variance and PC2 explaining 22.5% of the total variance (Supplemental Figure S27). However, PCA did not allow us to further differentiate within these groups while the SOM clustering provided clearly differentiated clusters. This is illustrated in Supplemental Figure S27 when the points in the PCA are colored by their corresponding SOM cluster. We also colored the points in the PCA by the predicted maximal growth rate according to normalized codon usage bias (dCUB) Normalized codon usage bias (dCUB) is a metric that examines the frequency at which alternative codons are used in different expressed genes and is a predictor of maximum growth rates in uncultured organisms. It is a unitless metric where larger negative values denote faster growth while small negative or positive values denote slower growth. Most notably, dCUB values that are greater than a critical threshold of -0.08 are considered to be indistinguishable and are broadly treated as representative of oligotrophic organisms. Analyzing these dCUB values across the whole dataset demonstrated a clear trend of faster growing genomes clustering together in the upper right quadrant (Supplemental Figure S28). Direct hierarchical clustering was computationally infeasible on the full dataset.

We used a hexagonal, toroidal grid configuration to build the SOM in order to avoid the development of edge effects where the majority of data ends up in the corners of the map. Training a toroidal map connects these edges together fluidly to negate these edge effects (e.g., the top and bottom of the map are treated as adjacent). Adjusting for potential edge effects was necessary because, although the growth sensitivity results are continuous on a scale from 0-1, we observed bimodal distributions for most of the sensitivity tests where either a model was sensitive to the removal of a substrate (value between 0.8 and 1) or was insensitive to the removal (value between 0 and 0.2) (Supplemental Figure S4). Due to this bimodality, we also adjusted the learning rate vector from the default (0.05, 0.01) down to (0.025, 0.01). This reduced the learning rate for the map in early iterations to produce a smoother set of map values and combat the effect of bimodal data to tend towards extremes. We tested the number of iterations needed to achieve small quantization error values (discussed further below) (Supplemental Figure S26b) and increased the run length to 1500 iterations from the default setting of 100 iterations, in part due to the reduction in the initial learning rate which slows down convergence.

We also conducted a sensitivity analysis on the size of the map from 5-by-5 to 100-by-100. Determining map size for SOM clustering is an open problem, and there are no definitive theoretical bases for defining a “correct” size based on the size and characteristics of the input data(*63–65*). Heuristic rules of thumb have been suggested(*81*) as well as field-specific guidelines(*64*). However, the guidelines are not generalizable and remain dependent on data

characteristics. Metrics of error have been proposed(*66*, *82*) to identify if a given set of map parameters generates a SOM with an appropriate level of resolution (quantization error) or topology preservation (topographic error). We computed quantization error which measures the resolution of a SOM by looking at the difference between a data vector and its best mapping unit (BMU). The final map size is a balance between a map that is large enough to fully differentiate all of the patterns in the dataset and one that does not contain too many unassigned nodes. We chose a 20-by-20 grid size such that the map had sufficient space to distribute the variability in the data without overfitting the data. Quantization error decreases with increasing map size so map size cannot be optimized to a global error minimum but only a local minimum(*64*). Borders of unassigned nodes between clustered mapping units are one way that a sufficiently large map size is determined. The final map had 132 unassigned nodes (33%).

To identify clusters amongst the SOM nodes, we used k-means clustering. We also tried hierarchical clustering but elected to use k-means because it consistently performed better based on intra-cluster distances. The number of clusters $k = 8$ was chosen to minimize the intra-cluster difference while not overfitting the map (Supplemental Figure S26d). The final map is shown in Supplemental Figure S26a colored by cluster. When visualized in two-dimensional space this toroidal map appears as roughly square but the top and bottom, and left and right, sides of the map are continuously connected.

To test the stability of our clustering, we generated a set of 100 unique SOM maps and k-means clustering from our starting set of 1,478 genome ensembles and compared these independent cluster classifications using the Adjusted Rand Index (ARI) (*Methods 2.7*). The ARI describes how strongly correlated two independent clustering assignments are, and does not require exact label matching which is important given that the numeric labels of k-means clustering can shuffle randomly even for strongly conserved clusters. To capture the full variability of solutions captured by the CarveMe models, for each of the 100 SOM maps we randomly selected a single model from each genome rather than representing all 60 individual models in an ensemble. To baseline our ARI comparisons, we also drew 100 sets of random cluster assignments from a uniform distribution of the cluster values. The mean pairwise ARI values between all combinations of these 100 random assignments was $ARI = -4.88 \times 10^{-5}$ which aligns with the standard interpretation of an ARI of approximately 0. By comparison, the mean pairwise ARI value between our 100 independent SOM/k-means re-runs was 0.81, indicating strong correlation between the cluster assignments across all 100 runs. Furthermore, since the individual model selected for each data subset was random, we show that there is no substantial impact for using a specific "representative" model. Comparing these 100 unique clustering assignments to our final assignment using all 60 models per genome also showed a strong mean ARI of 0.85, supporting our choice to use the sensitivity values of all 60 models in our training data.

In addition to using ARI to test the stochasticity of re-running the SOM training and k-means clustering on the same or similar data, we further compared our 100 unique maps/clustering assignments against maps/clustering generated from a subset of the data. Specifically, we built a map and assigned clusters to data thresholded at 90% consensus rather than 80% consensus. This

reduced dataset contained only 963 genomes. Again, we found a mean ARI of 0.85 when comparing to the set of cluster assignments generated on the 80% consensus data. This analysis of cluster stability demonstrates that, while there is some fuzziness in our clusters, the overall groupings and patterns we find are robust and consistent across independent runs and alternative data thresholding.

S3 SOM Cluster Analyses

To understand the underlying metabolic niches identified by the SOM clusters, we performed a variety of additional analyses. For each SOM cluster, we determined the set of compound classes that resulted in reduced growth when limited (hereafter called the growth sensitivity profile). We also calculated the genome estimated maximum growth rates for each cluster using codon usage bias (dCUB) and assessed the taxonomic composition of each cluster. For these analyses, we used the dCUB threshold value of $dCUB = -0.08$ from(*24*, *25*) to differentiate between fast growing (more negative dCUB values) and slow growing (more positive dCUB value) organisms.

S3.1 dCUB Growth Estimates

We tested whether there were statistical differences in genome estimated maximum growth rates for the eight SOM clusters. Based on the results of our Wilcoxon signed rank tests, we identified five distinct groups of growth distributions (Supplemental Figure S8). We defined these five groups based on predicted maximum growth rates from slow (Cluster 5) to fast (Cluster 6). Some groups were indistinguishable from their neighboring groups on this continuum and were thus defined as a mixture of their neighboring groups' phenotypes. For example, Clusters 1 and 8 were neither distinguishable from slow growth Cluster 5 nor from intermediate growth Clusters 2, 4, and 7. However, Cluster 5 was statistically slower than the other 7 clusters. Thus, we labeled Clusters 1 and 8 as slow-intermediate. It is important to highlight that dCUB values were not included in building the SOM map and so any differences in dCUB values between clusters are emergent rather than prescribed.

Genomes in Cluster 6 were predicted to be significantly faster growers (Supplemental Table S6) than all clusters based on codon usage bias (average dCUB of -0.235), while Clusters 1, 5, and 8 all had significantly slower predicted growth rates (average dCUB ranging from -0.085 to -0.120). Intermediate growth Clusters 2, 3, 4, and 7 were significantly slower than fast growing Cluster 6 (average dCUB of -0.163) but significantly faster than the slow Cluster 5. Cluster 3 was significantly faster than both slow Cluster 5 and slow-intermediate Clusters 1 and 8 This suggests the possibility of distinguishing not just a general intermediate growth phenotype, but identifying a continuum of growth phenotypes.

We performed an additional test to determine whether the number of 'fast growers' found in each cluster could have occurred by chance. Specifically, we assign a designation of 'slow' or 'fast' growth to the 1,478 genomes in the dataset. Since dCUB values greater than -0.08 are not

distinguishable, we used this value as a cutoff where dCUB values above -0.08 were classified as generically 'slow growers' and values below -0.08 were classified as 'fast growers'. We then performed a bootstrapping analysis where we constructed 10,000 artificial clusters of 185 randomly selected genomes, chosen to reflect the mean number of genomes in our 8 SOM clusters, and calculated the percentages of 'fast growers' in each cluster (Supplemental Figure S9). This analysis confirmed that the 4 SOM clusters classified as oligotrophic had significantly lower percentages of 'fast growing' genomes than would have occurred randomly in the dataset, while the 2 clusters classified as copiotroph and fast intermediate had significantly greater percentages.

Both analyses suggested that there were meaningful differences in the percent 'fast growers' between the four "intermediate growth" clusters (Clusters 2, 3, 4, and 7). Specifically, Clusters 2, 4, and 7 had 60.6% and 67.6%, and 60.5% of genomes classified as 'fast growers', respectively, while 71.9% of genomes in Cluster 3 were 'fast growers'. For comparison, 79.5% of genomes in Cluster 2 were 'fast growers' while on average 42.7% of genomes in the slow growing clusters (range 38.2-47.7%) were 'fast growers' (Supplemental Figure S8). We posit that genomes in the 'intermediate growth' clusters could belong to an intermediary lifestyle phenotype(s) between copiotrophs and oligotrophs. Furthermore, our results hypothesize the existence of more than one of these intermediary phenotypes. Overall, our results suggest that there are multiple growth strategies for the 'intermediate' lifestyle and several growth strategies for the slow growing oligotrophs. In contrast, we identified only one growth strategy for the fast growing genomes. Our inability to differentiate multiple fast growing groups might be a bias in the model formulation as we are only able to differentiate the genomes based on the sensitivity to the 11 compound classes tested.

S3.2 Growth Sensitivity Profiles

We observed substantial differences in the metabolic strategies between all eight of our SOM clusters (Supplemental Figure S5b). Generally, we found that our SOM clusters fell into one of three distinct metabolic strategies, and that these strategies aligned with the five statistically different growth strategies identified above. The slow growing clusters demonstrated high growth sensitivity to two or more compound classes. For example, slow growing Cluster 5 demonstrated substantial sensitivity to both carboxylic acids and peptides. All three of the slow growth clusters had unique pairings of growth sensitive compound classes. By contrast, our fast growing Cluster 6 demonstrated low to zero sensitivity to any compound classes. Our four intermediate growth clusters each demonstrated a single compound class sensitivity, with our three slower intermediate growth Clusters 2, 4, and 7 demonstrating sensitivity to amino acids, carbohydrates, and B vitamins respectively, while the faster intermediate growth Cluster 3 showed sensitivity to carboxylic acids. Overall, carboxylic acids (3 clusters), amino acids/derivatives (3 clusters), and peptides (2 clusters) were the compound classes that caused the most significantly high sensitivities amongst our eight SOM clusters.

S4 Biogeographic Distribution

We examined the geographic distribution and relative abundances of our SOM clusters using two distinct methods of comparison across multiple datasets. Specific details on how each of these two methods were performed can be found in *Methods 2.9*.

To broadly assess the fraction of the heterotrophic community captured by our clusters, we aligned the genomes from our study to previously reported amplicon sequence variance (ASV) data from two north-south ocean transects, Atlantic (GA02) and Pacific (P16). There were 3,956 unique alignments that met our quality thresholds that were then used to examine the relative abundances of ASVs associated with each of our clusters.

Since the above analysis only mapped to the 16S rRNA sequence, to provide an alternative assessment of the geographical distribution of our SOM clusters, we also performed a competitive read recruitment of the 1,478 genomes in our study against a survey of 1,209 globally-distributed metagenomes (SRAs in Supplemental Table S3). We compared the number of reads that our genomes mapped to against the number of unmapped reads and found that our genomes recruited an average of 6.2% (range 0.02-68.6%) of all reads in a given metagenome (see Supplemental Table S3). (Note: these are full community metagenomes not filtered to only account for heterotrophic bacteria.)

Finally, we analyzed the relative abundance of the SOM clusters in the context of environmental metadata for a subset of our metagenomic stations that were taken from the Tara Oceans survey. This allowed us to expand beyond geographic distributions to examine patterns in the biogeography of our SOM clusters in different environmental ecotypes.

S4.1 ASV Analysis

We observed significant variation in the enrichment of different SOM clusters along the P16 and GA02 transects. Broadly, we saw that the fraction of the heterotrophic community that mapped to our SOM clusters were dominated by slow growing Clusters 5 and 8 with very rare amounts of the faster growing Clusters 3 and 6 (Supplemental Figures S12 and S14). This is consistent with the overall oligotrophic nature of the stations along this transect. We found that the 773 genomes with aligned 16S rRNA gene sequences accounted for on average 32.7% of the heterotrophic bacterial community along the P16 and GA02 transects based on ASV abundance (range 7.9-73.6%). In surface ocean samples (≤150 m), our coverage was even better with a mean of 44.3% (range 11.9-73.6%). We also identified some interesting depth-dependent patterns. Specifically, in surface ocean samples (≤ 150 m) our coverage was even higher with a mean of 44.0% (range 11.9-64.2%) (Supplemental Figures S13 & S15). Along both transects, Cluster 7 was observed to be one of the most abundant clusters in the upper ocean but absent or significantly reduced at depth. In contrast, Cluster 3 was absent or present only at very low abundances in the upper ocean samples but was found enriched in the P16 subsurface samples, particularly in those >500m.

Finally, we evaluated the effect of percent identity stringency on our results. While we prefer the more conservative 98% threshold, at 95% sequence identity our collective genomes relative abundance increases to 40.8% on average (range 15.6-73.4%) across all depths and 56.7%

on average (range 22.6% - 73.4%) in the upper 150m. It is important to highlight that these abundance measurements arise from only roughly half the dataset used to generate the SOM clusters (773 of 1,478 genomes) because many of our genomes were metagenome assembled genomes (MAGs) and MAGs often do not assemble full 16S rRNA. Since our initial dereplication of genomes was performed at 95% ANI, were we to have recovered 16S rRNA gene sequences from the remaining 705 genomes, these would very likely have represented additional unique ASVs. Therefore, we consider our cluster abundance estimates using this method to be quite conservative.

S4.2 Read Recruitment Analysis (RPKM)

Significant variations in the enrichment of different SOM clusters were observed across the 1,209 surveyed metagenomes (*Methods 2.9*, Supplemental Figures S16 & S17). When comparing the bootstrapped relative abundance values of our 8 SOM clusters (Supplemental Figures S18 & S19), we found that slow growing Cluster 8 was the most numerically dominant of our eight SOM clusters with a mean relative abundance of 29.8%, while the intermediate growing Cluster 4 was the least abundant with a mean relative abundance of 2.4%. Our fast growing Cluster 6 was the fourth most abundant with an average relative abundance of 11.4% while the intermediate-fast Cluster 3 was third least abundant with an average relative abundance of 9.58%. Cluster 8 was found to be the most abundant relative to the other seven clusters in every oceanographic category except the Southern Ocean. We visualized these geographic patterns in a set of global maps for each cluster based on z-scores of the standardized relative abundance data (Supplemental Figure S20). Visual differences in cluster distributions across different biogeographical regions can be seen in Supplemental Figures S20-S23. For example, in the South Pacific, samples enriched in Clusters 7 and 8 are depleted in Clusters 1 and 3 (and vice versa). For the two Southern Ocean samples, one is enriched in Cluster 3 while the other is enriched in Cluster 5. Supplemental Figure S21 provides a comparison of cluster enrichment at the deep chlorophyll maximum. Supplemental Figures S22 and S23 provide zoomed in maps of the Chesapeake Bay and Baltic Sea.

When we looked within specific oceanographic categories, further patterns emerged. While overall Clusters 3 and 6 had low relative abundances, these two clusters were two to three times as abundant at estuarine stations, reaching mean relative abundances of 30.3% for Cluster 6 (highest abundance in estuarine regions amongst all clusters) and 18.3% for Cluster 3 (second highest abundance in estuarine regions among all clusters). The estuarine stations were highly diverse, with seven of the eight SOM clusters present at relatively high abundances at statistically similar levels (Cluster 4 was significantly rarer than the other clusters in the estuarine samples). The relative evenness of the majority of the SOM clusters at estuarine stations could indicate that the microbial community is being supplied with a diverse set of compounds at concentrations sufficiently high to support the metabolic requirements of a diverse group of organisms. This type of environment with diverse compound availability favors more balanced metabolic strategies and

higher maximum growth rates of the faster growing clusters which is consistent with the higher abundances of these clusters at estuarine stations.

The coastal stations were primarily dominated by the intermediate growth Cluster 2 (29.5% mean relative abundance) and slow growing Cluster 8 (23.6% mean relative abundance). Similar to the estuarine stations, the overall cluster evenness suggests that the microbial community at coastal sites are also being supplied a diverse set of compounds at sufficiently high concentrations to support the growth of diverse metabolic strategies. In particular, since the two most dominant clusters were sensitive to amino acids, their abundance in coastal locations suggests that those stations had high enough concentrations of amino acids to sustain large total biomasses of these organisms.

The remaining three categories - oligotrophic seas, oligotrophic open oceans, and the southern ocean - were dominated by just two of the eight SOM clusters. The oligotrophic seas and oligotrophic open ocean samples showed similar distributions of relative abundance and were both dominated by slow growing Clusters 5 and 8. The high abundance of slow growing organisms in these categories is consistent with these oceanographic regions being resource limited. In resource limited environments, organisms often cope with consistently low nutrient concentrations by specializing on specific compounds for growth resulting in an environment with rigid, defined niches(*83*). Organisms typically specialize in growth on certain compounds by using transporters with greater affinity for these compounds, and/or streamlining their genomes to reduce internal nutrient requirements. This sort of rigid niche structure, and low nutrient availability, is unfavorable for fast growing, metabolically flexible organisms as they can be outcompeted for compound acquisition by specialists. Resource limited conditions thus favor compound sensitive, specialist organisms occupying defined niches for growth on their specific compounds(*84*). The low abundances of slow growing Cluster 1 in the open ocean suggests then that the environmental niche(s) that this cluster occupies was not present.

S4.3 Comparison to Environmental Data

In addition to the analysis of geographic distribution using RPKM values from our metagenomic recruitment survey, we also were able to examine patterns based on environmental metadata for a limited number of samples from the Tara oceans metagenomic survey. These samples allowed us to not only look at changes in enrichment across the global ocean, but also what physical chemical characteristics of different water masses might be influencing these enrichment differences. We performed an NMDS of all of the environmental variables (Supplemental Figure S24) and identified three distinct clusters of data points, represented by the three convex hulls drawn on the plot, that separate based on distinct environmental characteristics. Broadly, these three data clusters broke down into three environmental ecotypes based on their position relative to major environmental axes: cold, high nutrient waters; warm waters with elevated chlorophyll (higher primary productivity); and warm, oligotrophic samples with low primary productivity and inorganic nutrients. We overlaid these environmental ecotypes onto our

NMDS of the SOM cluster relative abundances. We also added these hulls to the NMDS of just the Tara oceans samples (Supplemental Figure S24b). Clusters 3, 4, and 6 which are the faster growth clusters were enriched in cold, high nutrient stations while clusters 1, 7 and 8 (the slow growth clusters) were enriched in warmer waters with lower nutrients and varying levels of chlorophyll (a proxy for primary productivity) (Supplemental Figure S24). The ecotypes defined from this set of samples were also used in comparison with samples from other biogeographic regions, such as estuarine and coastal samples in main text Figure 3.

Supplemental Figures:

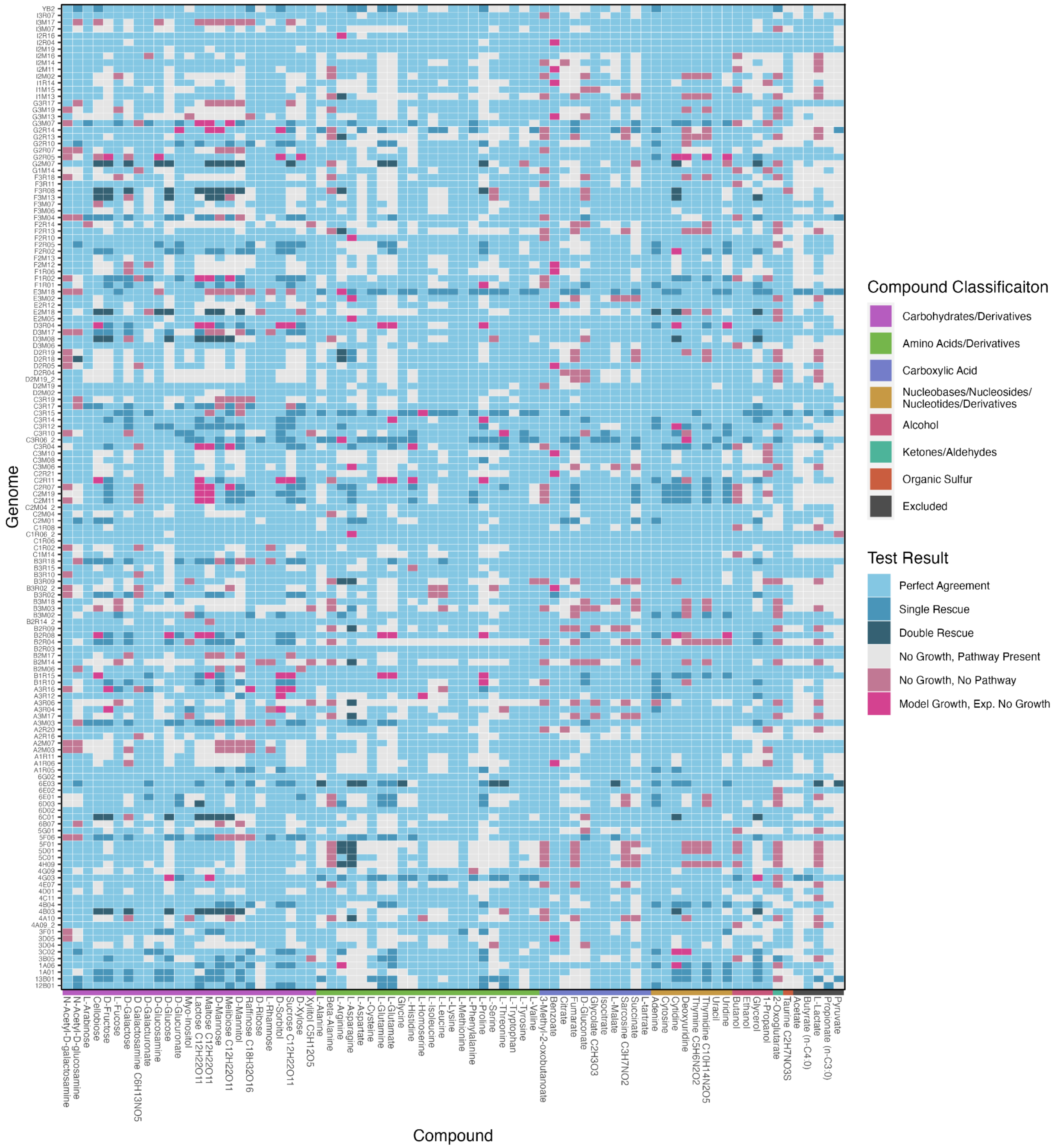

Supplemental Figure S1: Comparison of modeled and experimentally determined growth rates on a range of sole carbon sources. Heatmap of agreement between CarveMe model growth results and experimental growth data from Gralka et al. (*18*) for a collection of 146 strains grown on 77 different sole carbon sources. The specific classification of each compound according to our manually curated categories is shown as a sequence of colored bars along the x-axis. When comparing the growth/no growth predictions between CarveMe and the experimental data, we classified the results as 1) perfect agreement (light blue, 68.8%), 2) agreement when

supplementing the CarveMe models with one (single rescue, medium blue, 5.8% additional agreement) or 3) two (double rescue, dark blue, 0.96% additional agreement) carbon compounds. We also distinguished cases where growth was observed experimentally and the CarveMe model had the pathway to uptake the compound in question but did not demonstrate growth on it (grey, 18.9%), suggesting the model may have been missing key pathways to catabolize that compound. Finally, two types of 'problematic errors' were observed: 1) experimental data predicted growth but the CarveMe model neither grew on that compound nor had the pathway to uptake that compound (light pink, 4.5%), and 2) the CarveMe model predicted growth on a compound but those microbes showed no ability to grow on that compound in the experimental data (dark pink, 1%).

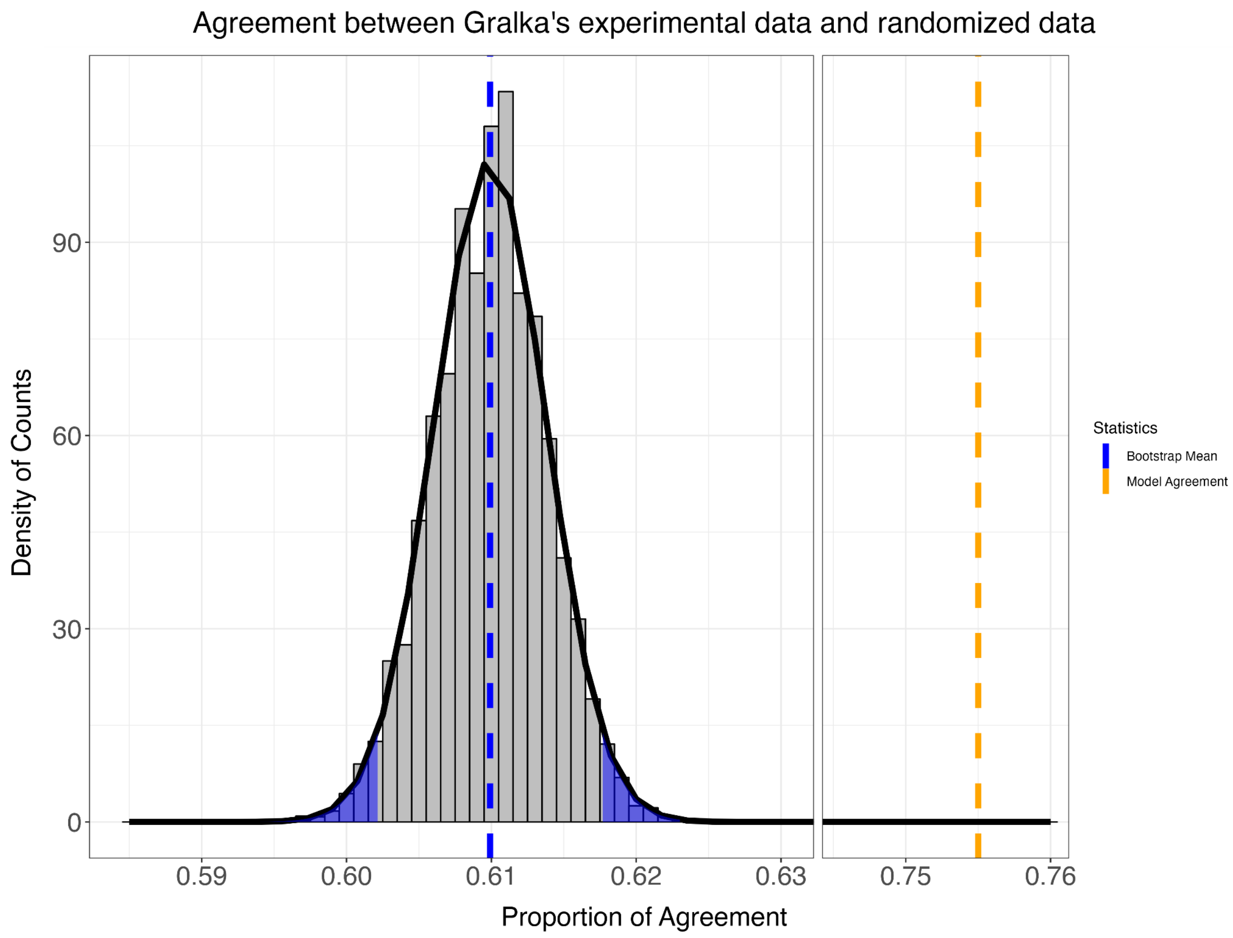

Supplemental Figure S2: Bootstrap experiment of model accuracy to experimental data. We performed a bootstrap analysis to compare the accuracy (model agreement) of the CarveMe model growth/no-growth predictions for the Gralka et al. (*18*) experimental results to the accuracy we would achieve using a randomized data schema. In this analysis the binary growth/no growth outcomes were randomized such that the randomized data had the same number of positive growth signals for each of the genomes from the experimental study. This was then simulated 10,000 times to establish a distribution of accuracy values using random data. The mean value of these measured accuracy values is shown with the blue dashed line, while the data beyond 2 standard deviations in each direction is highlighted by the blue shaded areas of the Gaussian curve drawn over this distribution. Our observed accuracy measurement is shown with the dashed orange line, sufficiently higher than the predicted mean accuracy with randomized data (note the break in the x-axis values).

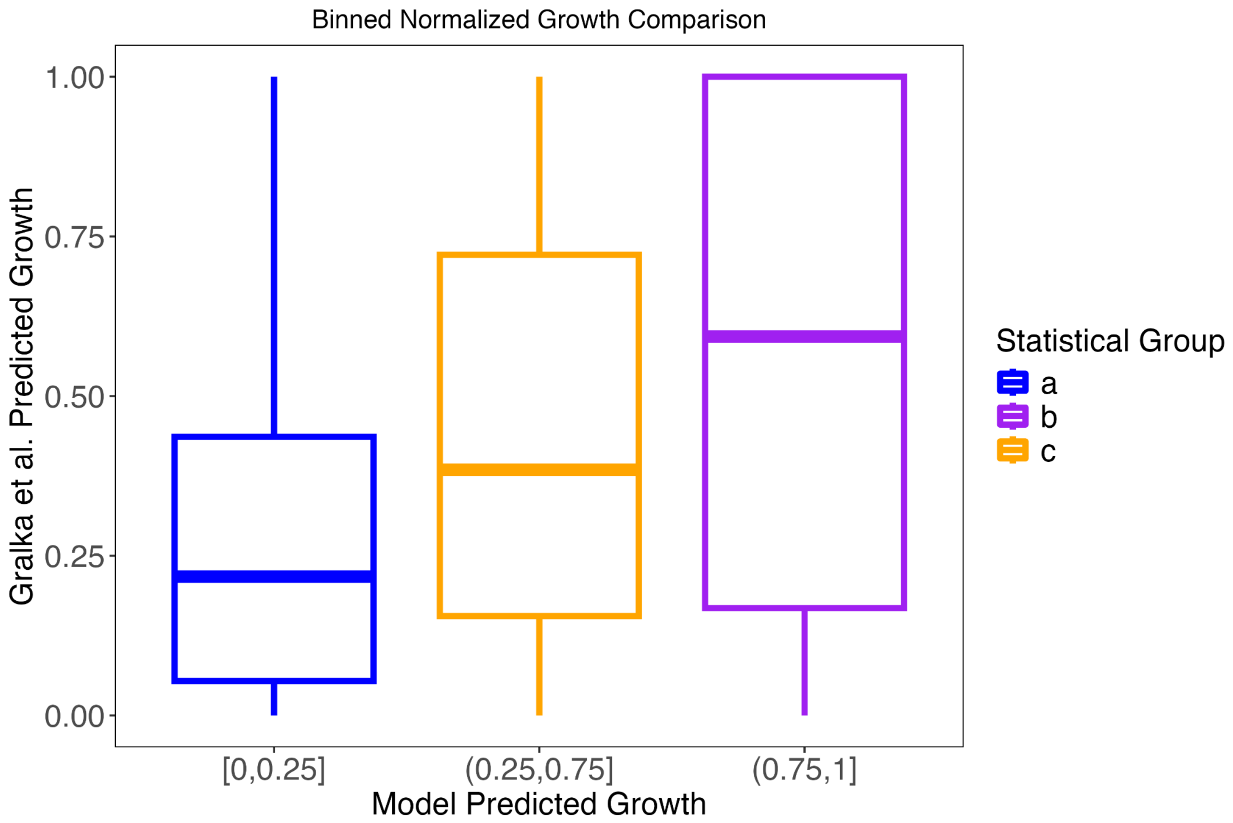

Supplemental Figure S3: Comparison of CarveMe modeled growth rates and experimental data. We compared our predictions of growth rates with the experimentally observed growth rates from the Gralka et al. (*18*) study. This plot shows the distribution of model and experimental growth rates normalized to the compound on which the fastest growth was observed per genome. We binned these growth rates into compounds on which there was relatively low growth rates (values in the range 0-0.25), intermediate growth (values in the range 0.25-0.75), and high growth rates (values in the range from 0.75-1). Statistical groupings were determined by an ANOVA test of the distributions of experimentally predicted growth rates in these three bins using Tukey's HSD with a 95% confidence interval.

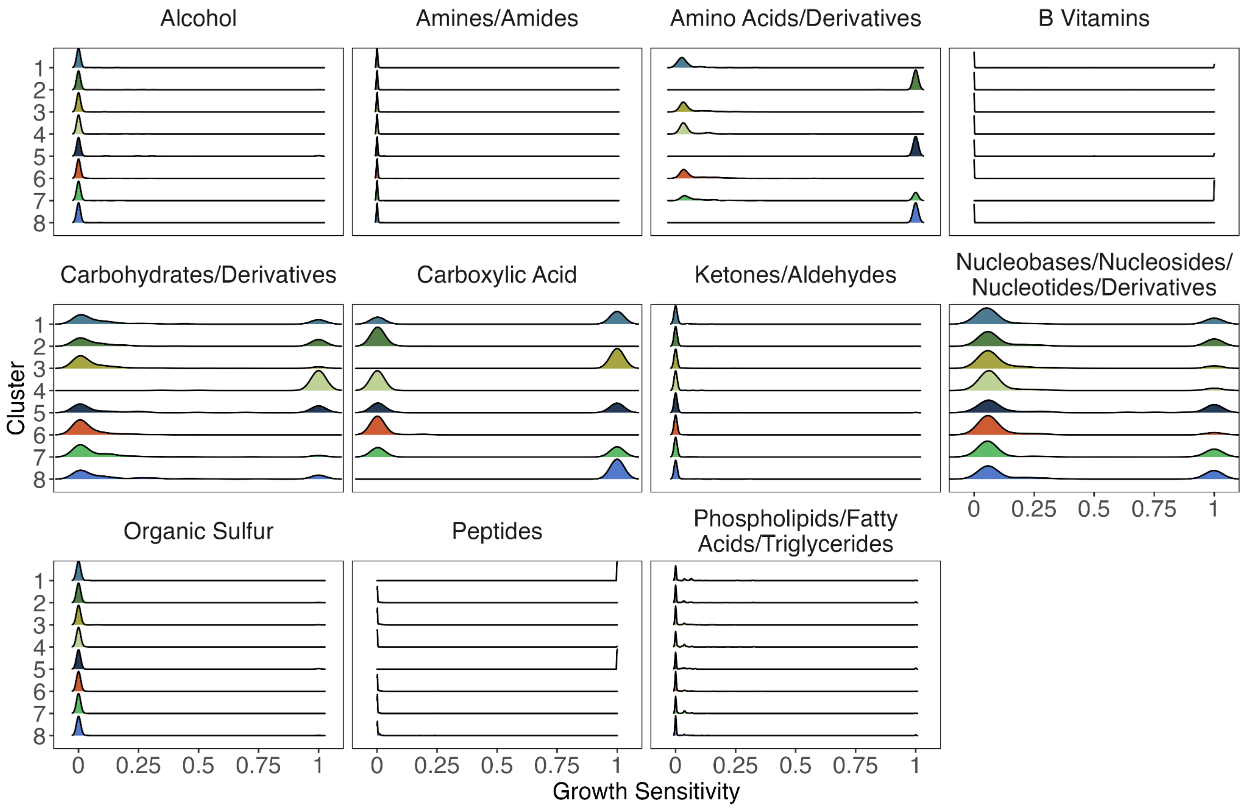

Supplemental Figure S4: Distribution of growth sensitivity values by cluster. Density plots of the growth sensitivity values for each model for each of the 11 compound classes grouped by SOM cluster.

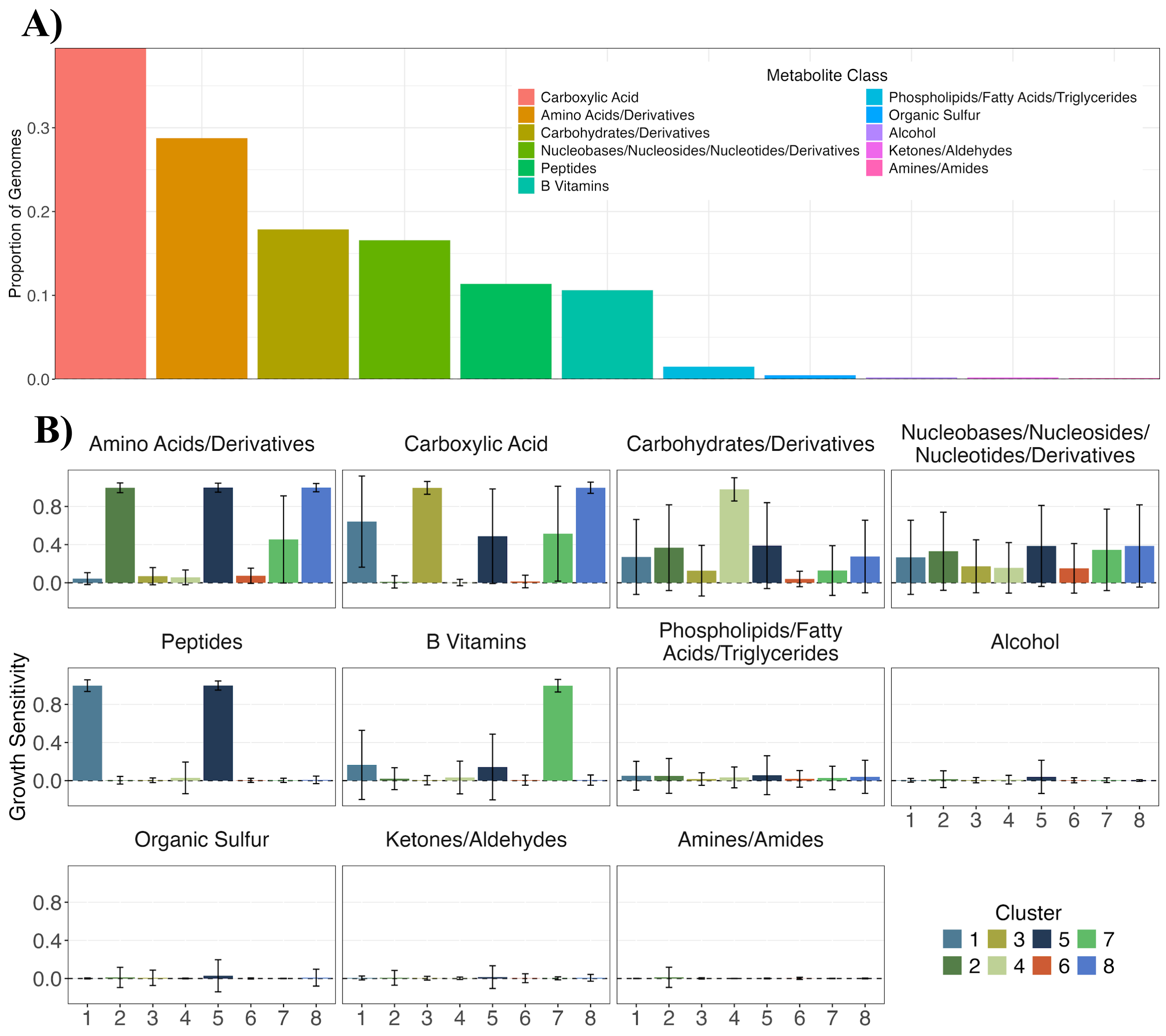

Supplemental Figure S5: Relative growth sensitivities between SOM clusters. (**A**) Ordered bar plot of the proportion of models across all clusters with substantial growth sensitivity to the reduction of each compound class (substantial is defined as >80% reduction in growth). (**B**) Bar plots of the relative mean growth sensitivity values for each of the 11 compound classes across the 8 SOM clusters. The error bars represent one standard deviation. Plot facets are ordered from the highest overall sensitivity (carboxylic acids) to the lowest (amines/amides).

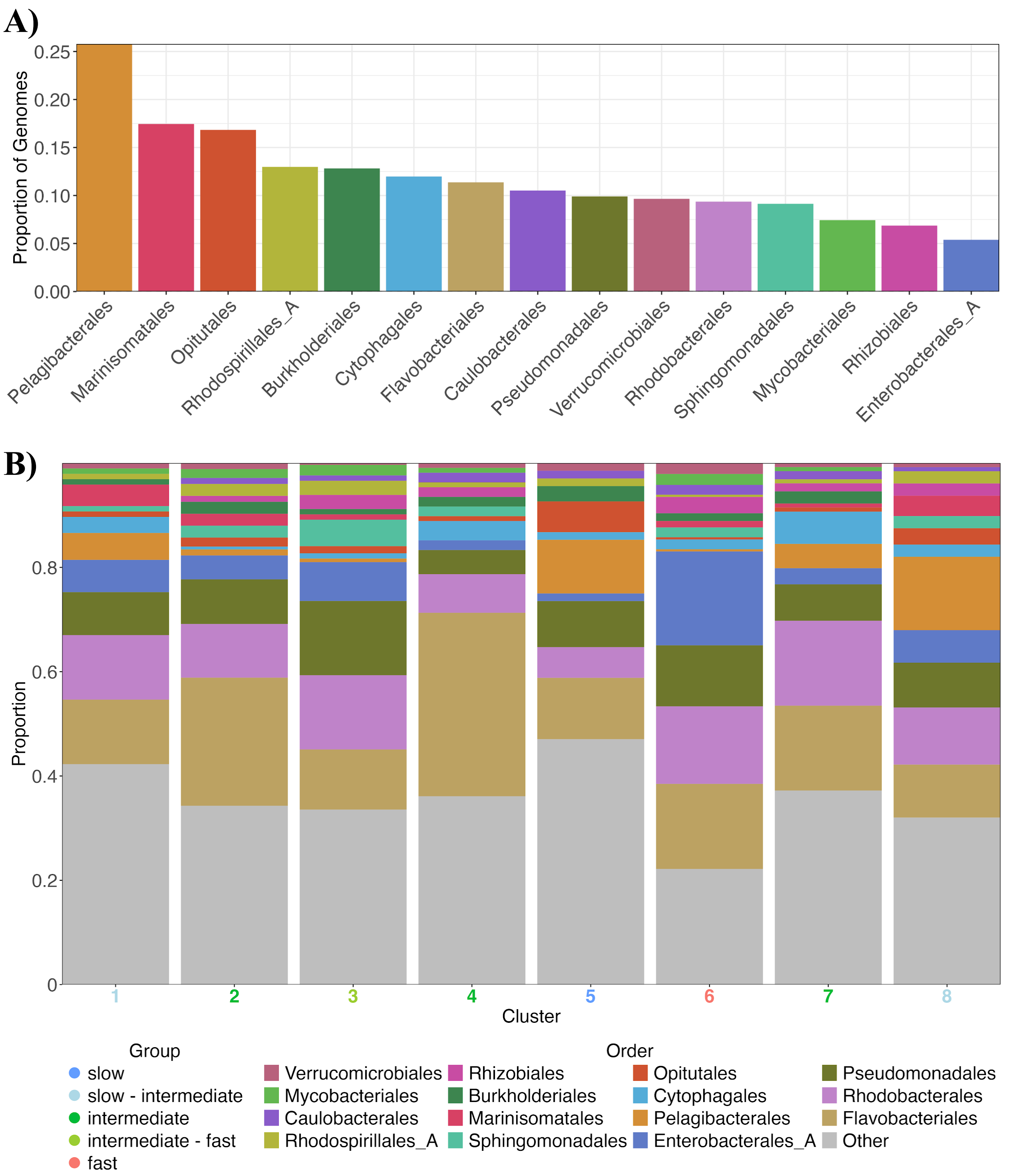

Supplemental Figure S6: Taxonomy by cluster. (**A**) Ordered bar plot of the proportion of models in each of the 15 most abundant orders with substantial growth sensitivity to the reduction of any compound class (substantial is defined as >80% reduction in growth). (**B**) Stacked bar plots of the relative abundances of the top 15 orders in each cluster.

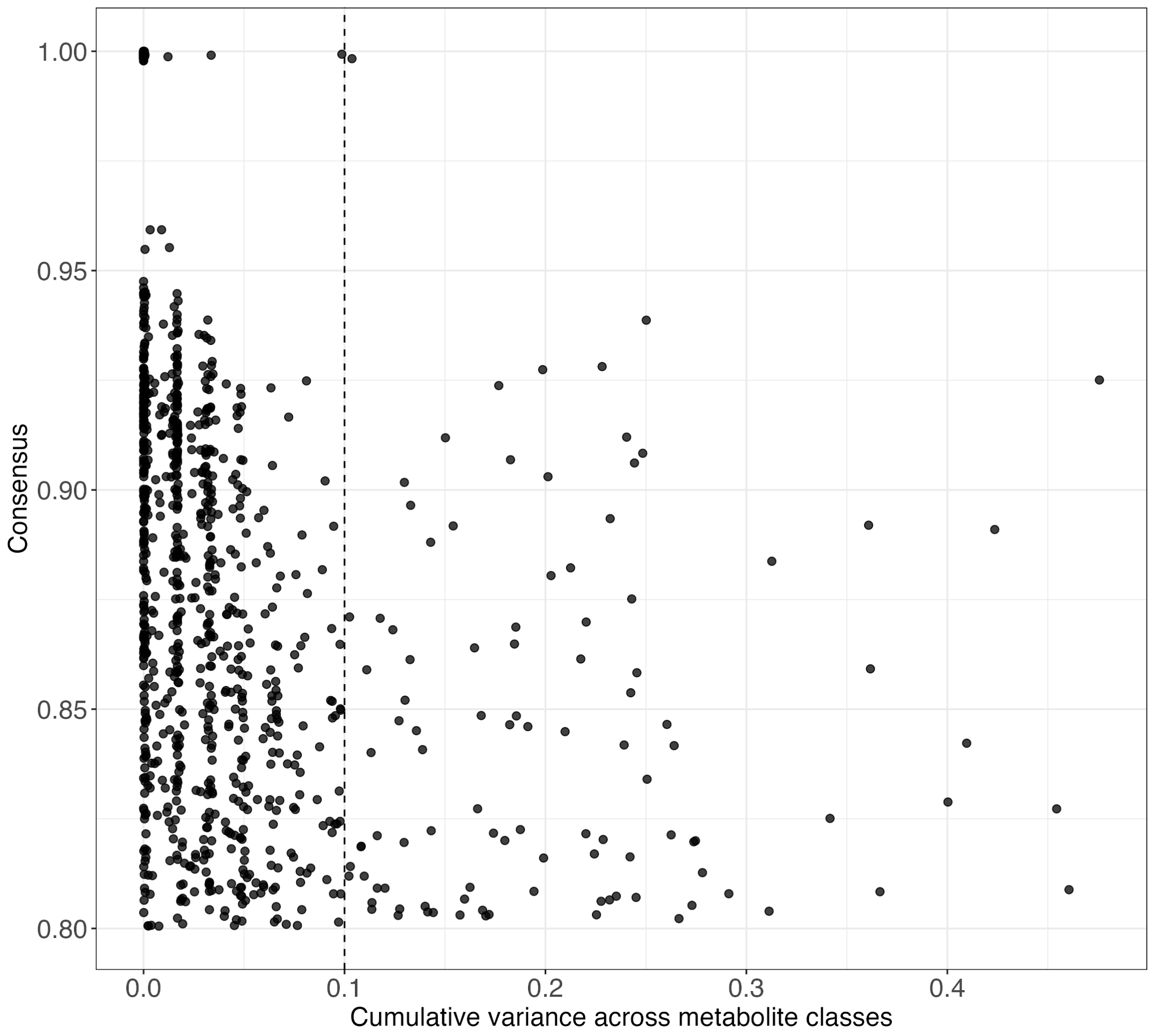

Supplemental Figure S7: Cumulative variance of growth sensitivity versus ensemble consensus. Scatterplot showing the cumulative variance in predicted growth sensitivity for the 60 models in each ensemble across all 11 metabolite classes against the ensemble consensus value for each genome. The dashed line represents a cumulative variance of 0.1 and demarks the threshold we used to filter out high variance outliers (N=100) from our set of 1,578 high quality genomes.

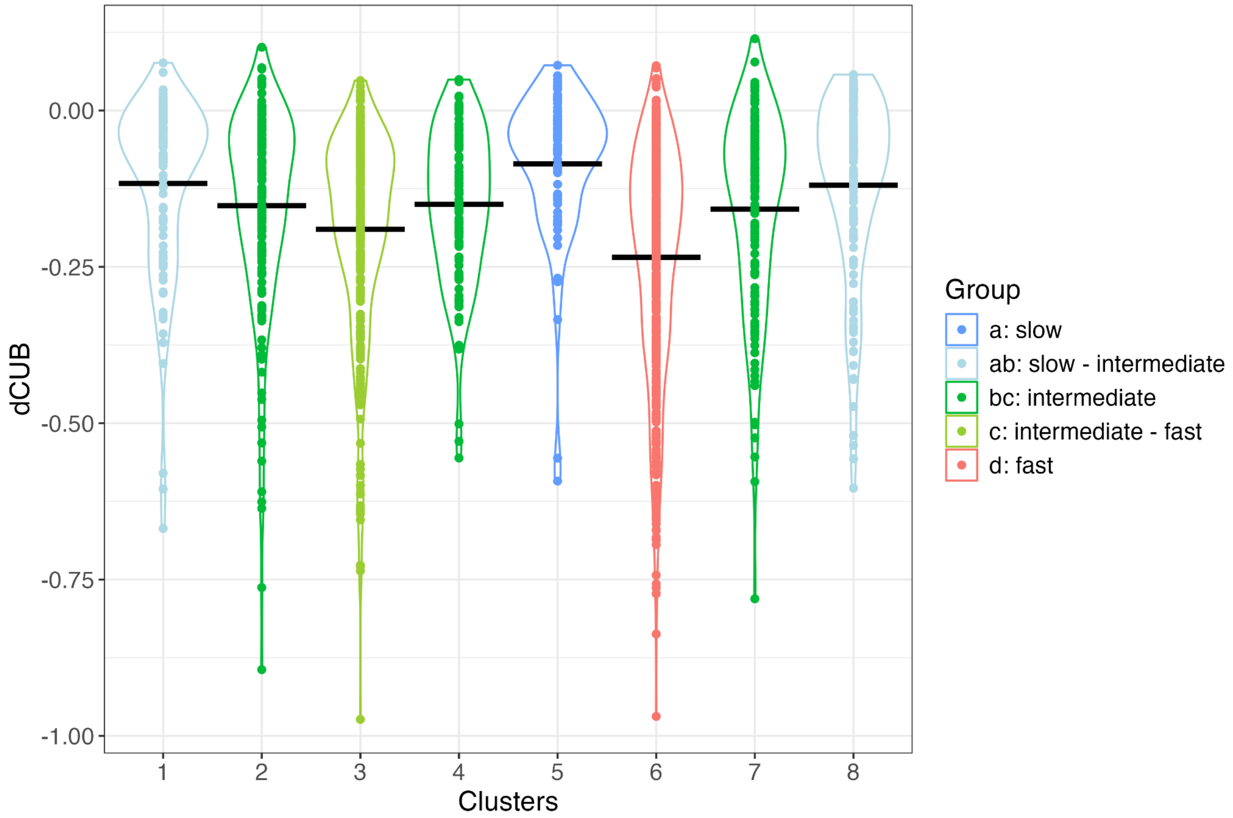

Supplemental Figure S8: Codon usage bias (dCUB) by cluster. Violin plots of the dCUB distributions for genomes assigned to each of the 8 SOM clusters. The horizontal black bars represent the mean value of each of the 8 distributions. The dCUB values fall into five statistically distinct groups designated with letters according to the key. Statistical groupings were determined by a pairwise Wilcoxon signed rank test of the dCUB distributions using Bonferroni correction and a 95% confidence interval. Group *a* is the slow-growing group (Cluster 5) and statistically distinct from the other clusters. Group *bc* (Clusters 2, 4, and 7) is the intermediate-growing group and significantly faster than the slow group. Group *d* (Cluster 6) is the fast-growing group and is significantly faster than all other clusters. Group *ab* (Clusters 1 and 8) is not significantly different from groups *a* and *bc* and reflects organisms that tend towards both slow and intermediate growth phenotypes. Group *c* (Cluster 3) is significantly faster than groups *a* and *ab* but slower than group *d* and reflects organisms that tend towards both intermediate and fast growth phenotypes.

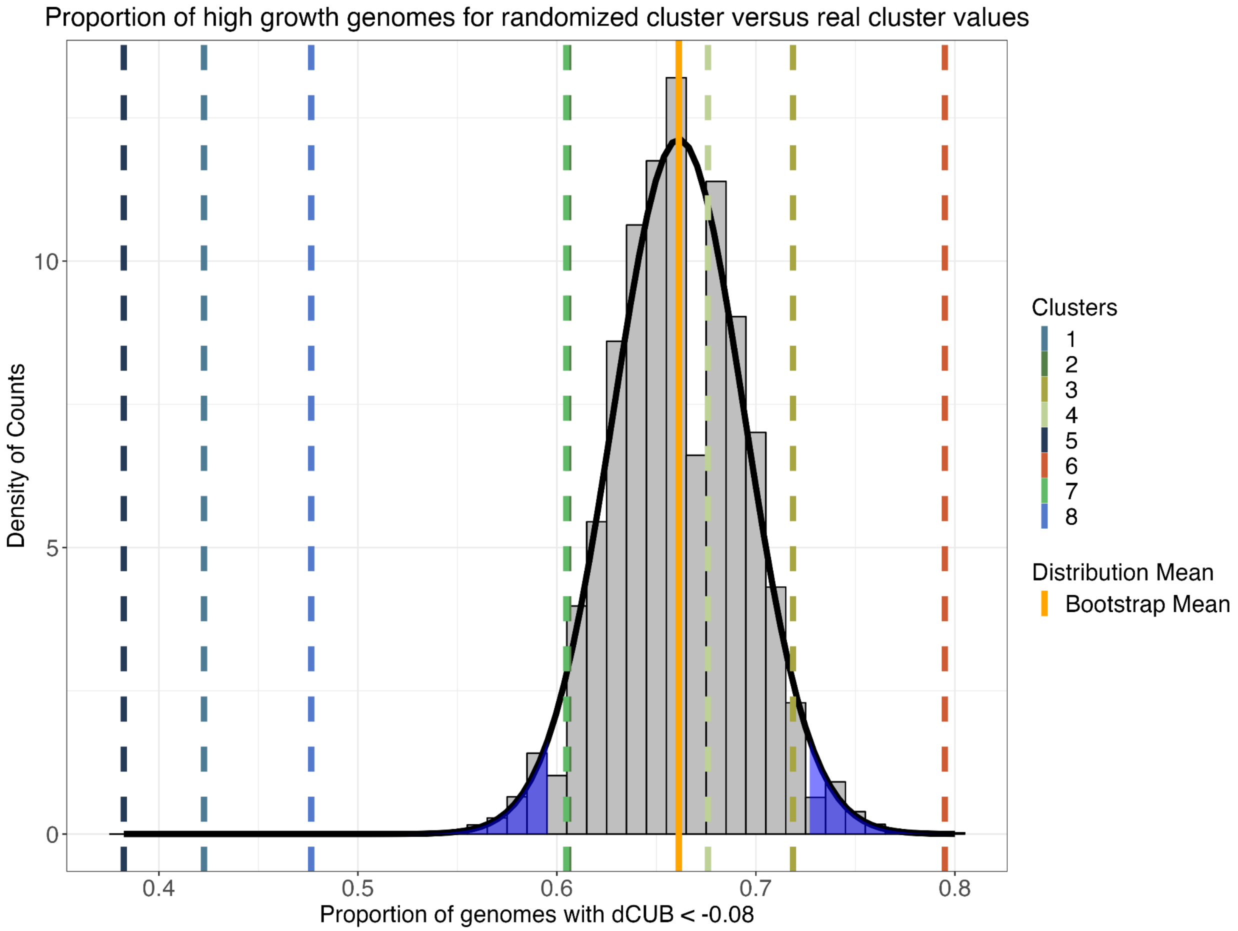

Supplemental Figure S9: Bootstrap experiment of dCUB growth rates for randomized SOM clusters. We performed a bootstrap analysis to compare the proportion of genomes below the critical dCUB threshold of -0.08 for clusters with randomly assigned genomes versus the real SOM clusters predicted by our method. The mean proportion from the bootstrapping analysis is shown in the dashed orange line. The area of the distribution beyond 2 standard deviations from this mean is highlighted in the blue shaded area of the Gaussian curve drawn over this distribution. The mean proportion of genomes below the critical dCUB threshold for our 8 SOM clusters are shown by the various dashed lines colored according to which cluster they represent. We see that our three intermediate growth clusters fall within our randomized distribution, which is unsurprising given that they represent an intermediate growth rate physiology. The 3 slow growing clusters fall more than 2 standard deviations below the bootstrap mean, indicating that those clusters grow more slowly than can be explained by random effects. Conversely, the fast and intermediate-fast clusters fall more than 2 standard deviations above the bootstrap mean, confirming that on average they grow faster than we would expect from a random group of genomes.

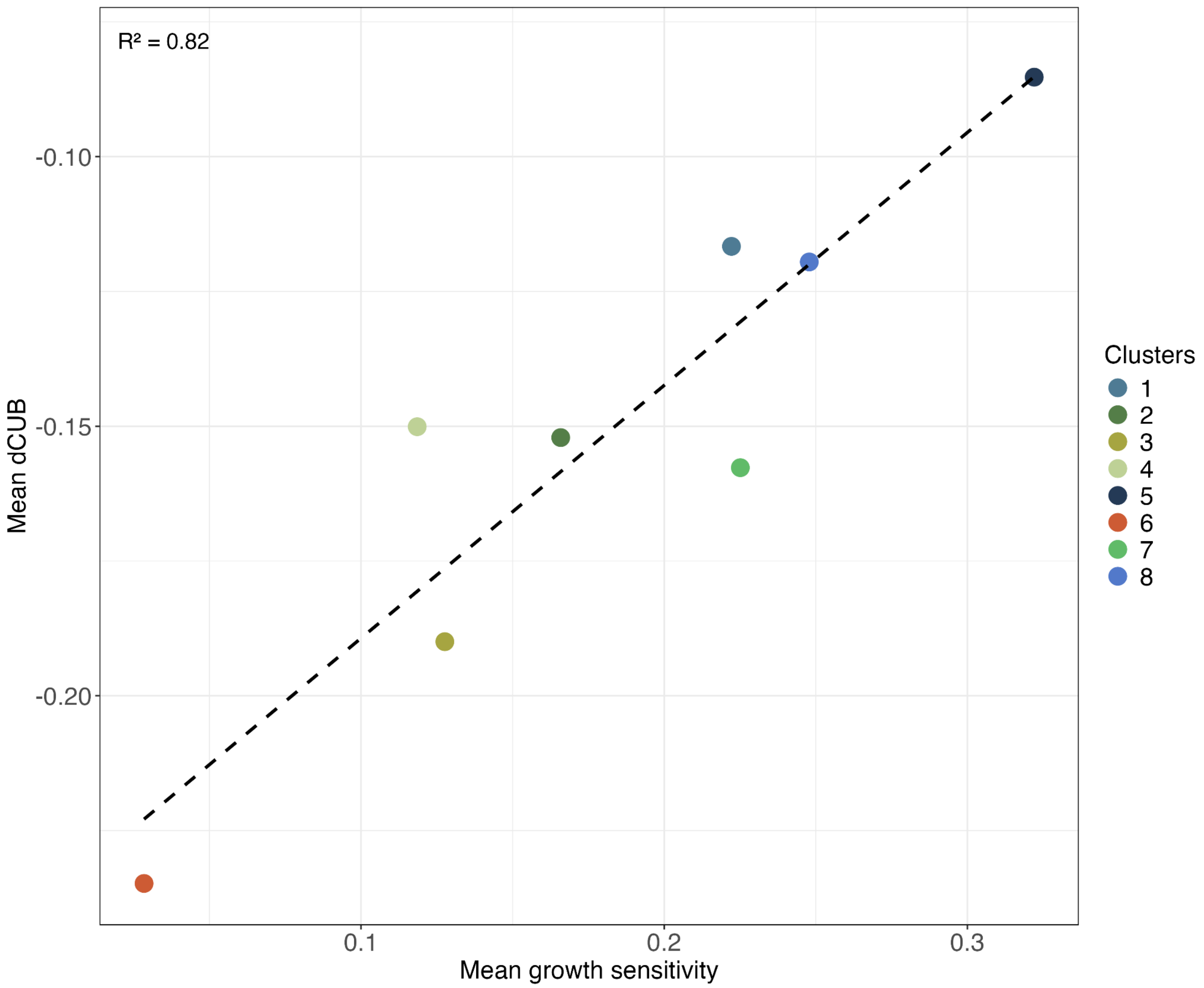

Supplemental Figure S10: Comparison between mean dCUB and growth sensitivity values per cluster. For each of the 8 SOM clusters we characterized their mean growth sensitivity value across all 11 compound classes as well as their mean dCUB values to examine the relationship between growth and metabolic sensitivity on average. A simple linear model was fitted to this data (black dashed line) and we found that the mean values of the two measures are strongly correlated with an adjusted $R^2 = 0.825$ and $p = 0.001$ with 6 degrees of freedom.

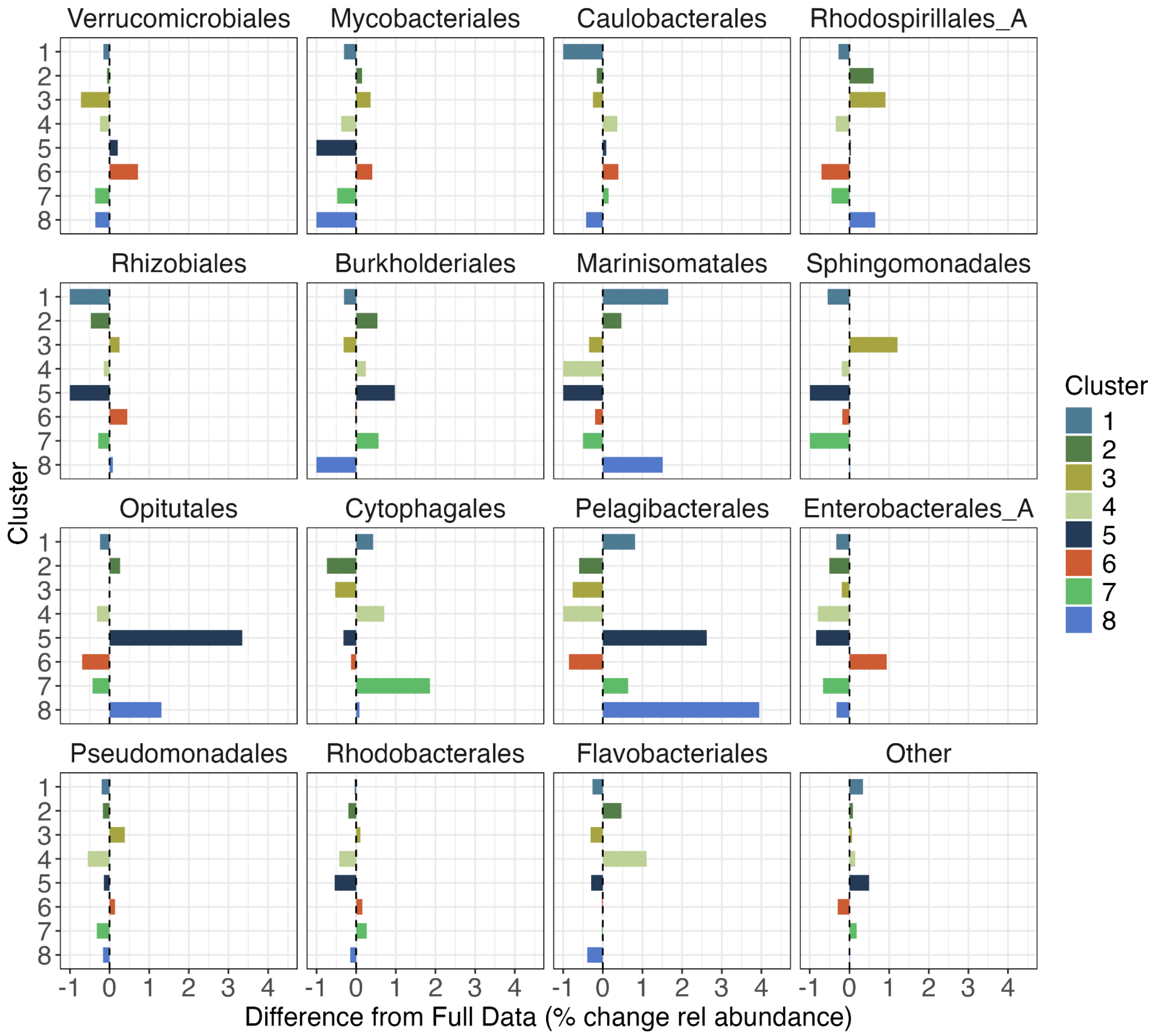

Supplemental Figure S11: Taxonomic abundance enrichments of top 15 Orders by cluster. Percentage enrichment in the relative abundance of the top 15 Orders (and Other) in each of the 8 SOM clusters compared to the relative abundances of each of these Orders in the full dataset.

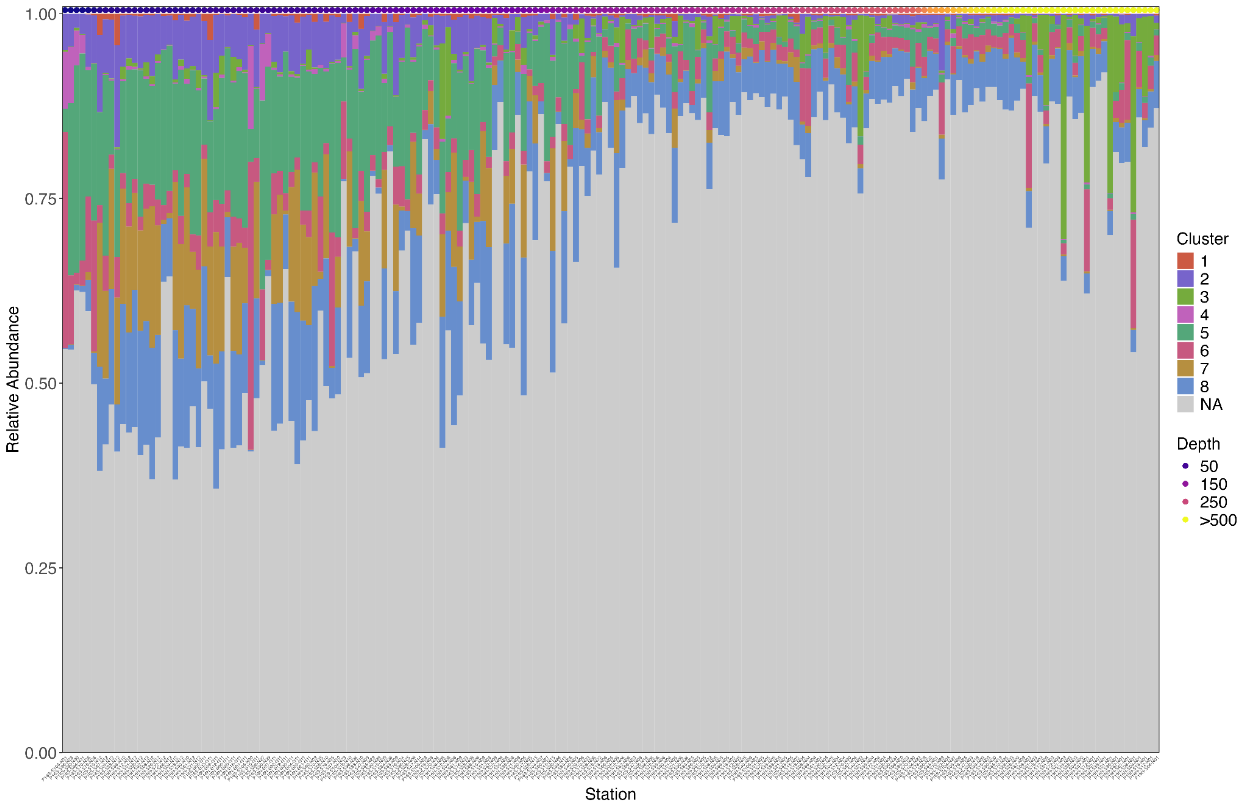

Supplemental Figure S12: Relative abundances of the P16 ASV alignments. Relative abundances of the 8 SOM clusters including the fraction of the station abundance attributed to ASVs not assigned to a SOM cluster. Stations are ordered from left to right by increasing sampling depth (capped at 500m). As depth increases, the fraction of the community abundance captured by genomes in our SOM clusters begins to drop substantially. The fraction of abundance that is unattributable to our genomes increases linearly until about 150 meters depth after which the unattributable fraction levels off around 80% of the total community abundance.

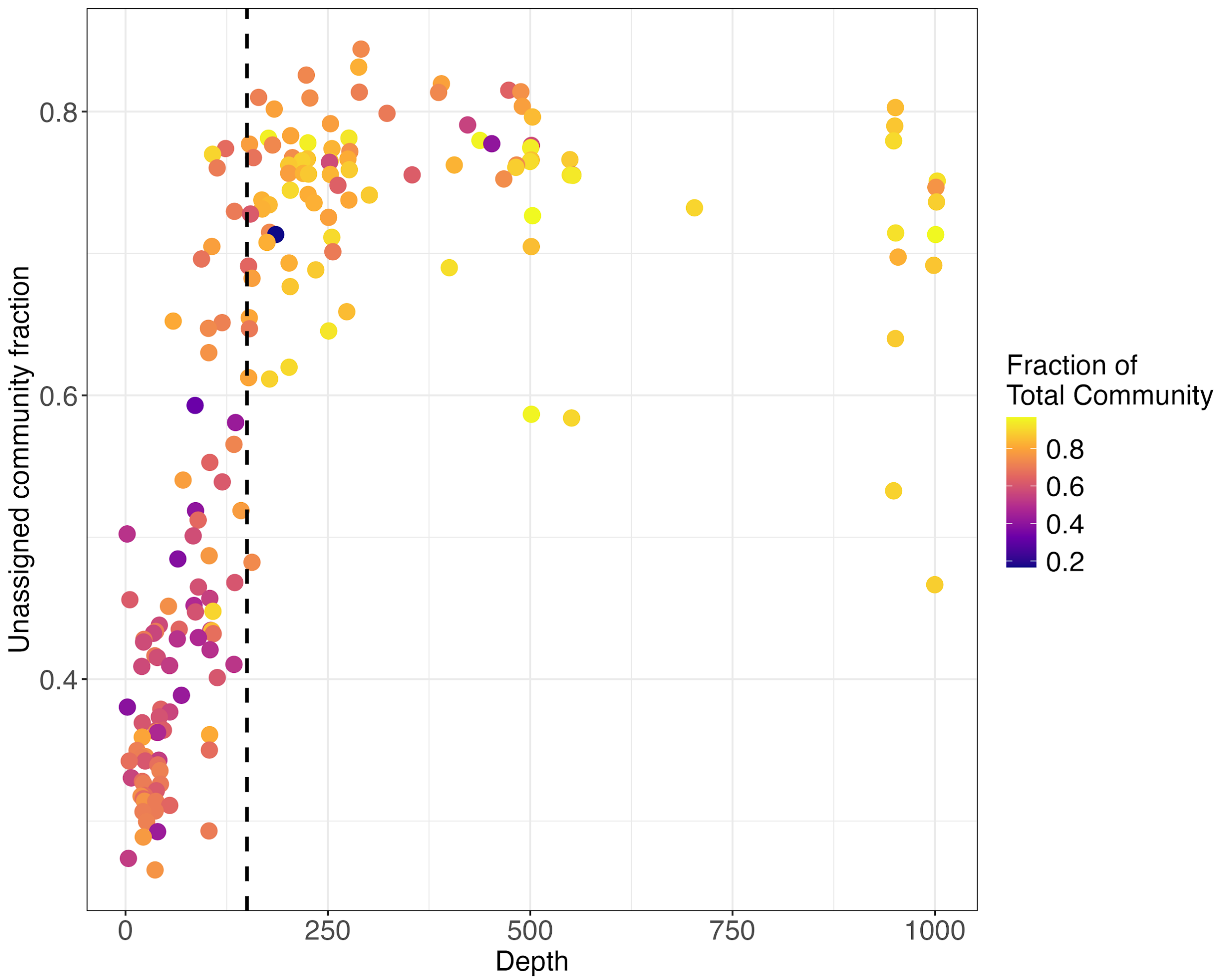

Supplemental Figure S13: Depth versus unassigned heterotrophic community fraction at P16. Scatterplot of the sampling depths and the corresponding fraction of the heterotrophic community that does not map to the ASVs aligned to our 773 genomes with 16S sequences for the P16 transect. Points are colored by the fraction of the total bacterial community represented by the heterotrophic community. The black dashed line delineates a depth of 150m.

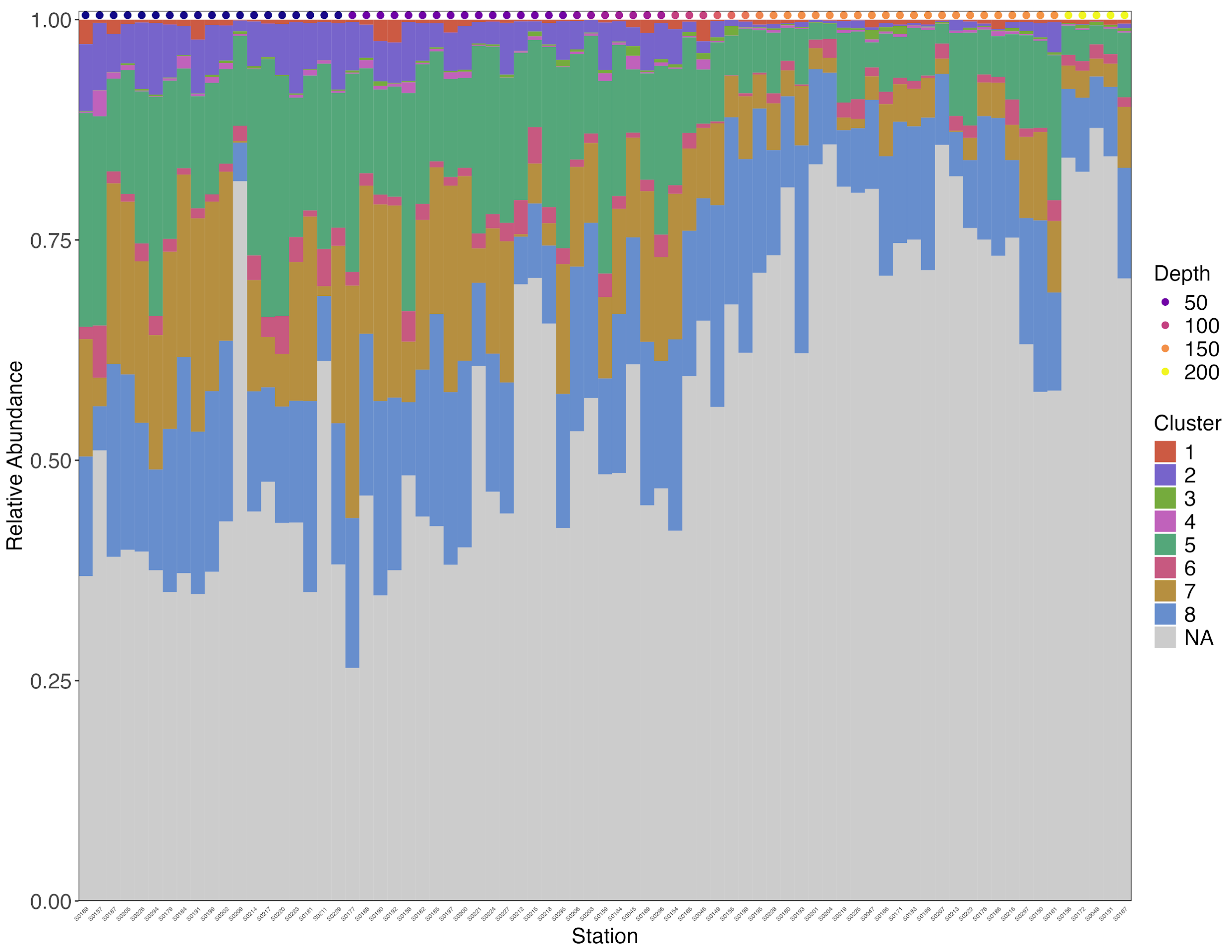

Supplemental Figure S14: Relative abundances of the GA02 ASV alignments. Relative abundances of the 8 SOM clusters including the fraction of the station abundance attributed to ASVs not assigned to a SOM cluster. Stations are ordered from left to right by increasing sampling depth. As depth increases, the fraction of the community abundance captured by genomes in our SOM clusters begins to drop substantially. The fraction of abundance that is unattributable to our genomes increases linearly until about 150 meters depth after which the unattributable fraction levels off around 80% of the total community abundance.

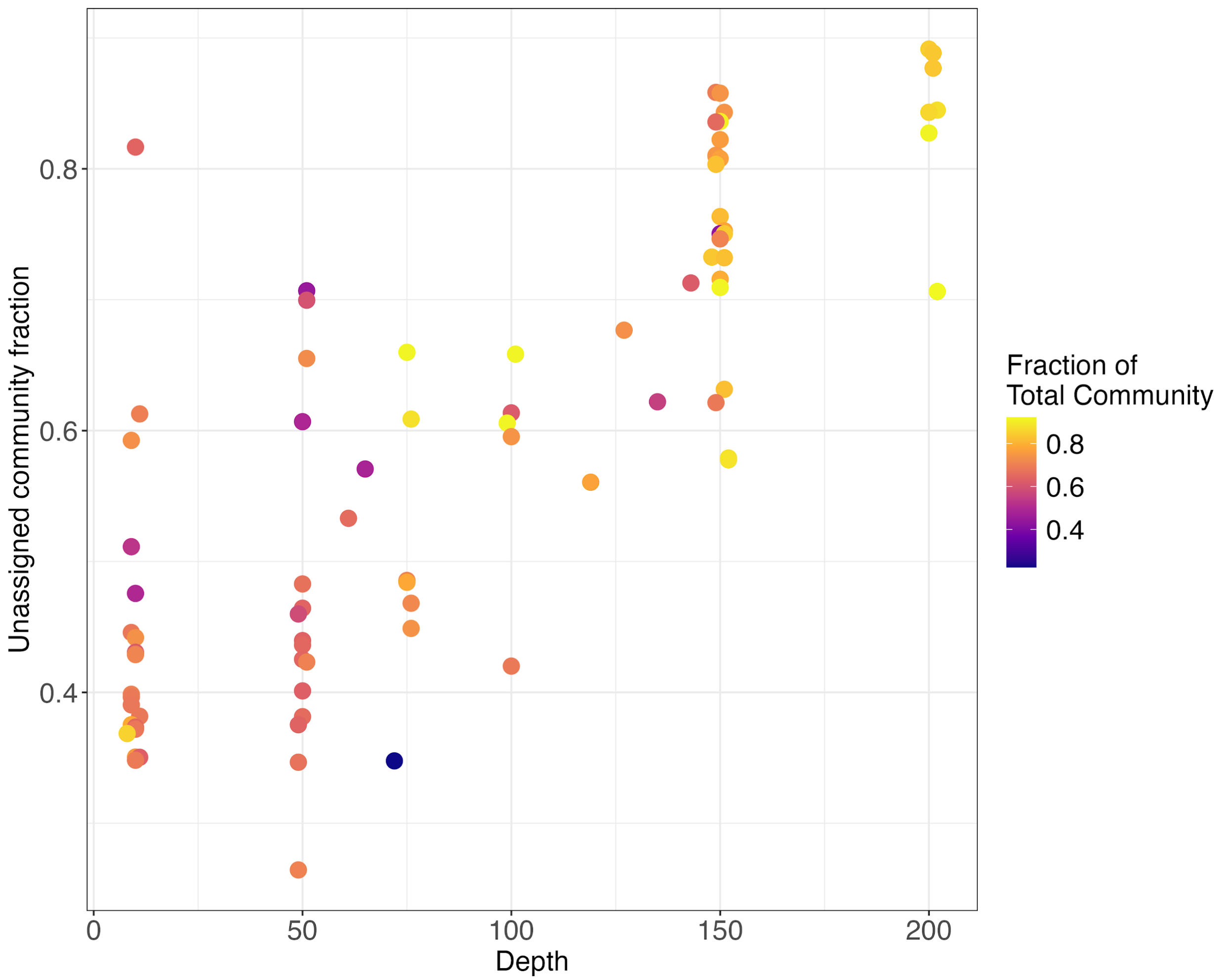

Supplemental Figure S15: Depth versus unassigned heterotrophic community fraction at GA02. Scatterplot of the sampling depths and the corresponding fraction of the heterotrophic community that does not map to the ASVs aligned to our 773 genomes with 16S rRNA sequences for the GA02 transect. Points are colored by the fraction of the total bacterial community represented by the heterotrophic community. The black dashed line delineates a depth of 150m.

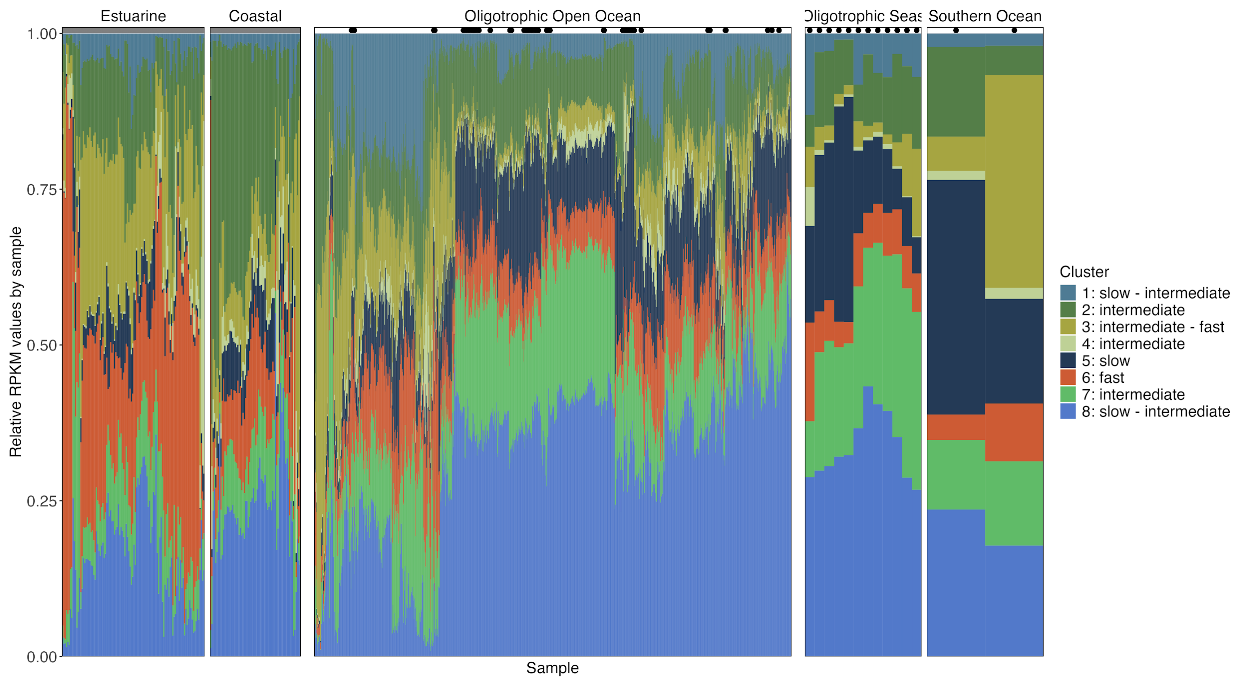

Supplemental Figure S16: Relative abundances by oceanographic regime. The 5 plot subpanels represent all of the samples from each of our 5 defined oceanographic regimes from classically high productivity Estuarine samples (left) to the classically more nutrient poor Southern Ocean samples (right). Relative abundances are colored by the associated SOM cluster and color palette denotes each cluster's growth strategy with fast growers in red, intermediate growers in shades of green, and slow growers in shades of blue. Abundances are ordered within each of the 5 regimes according to a McQuitty linkage clustering on the Bray-Curtis similarity values of the cluster relative abundances.

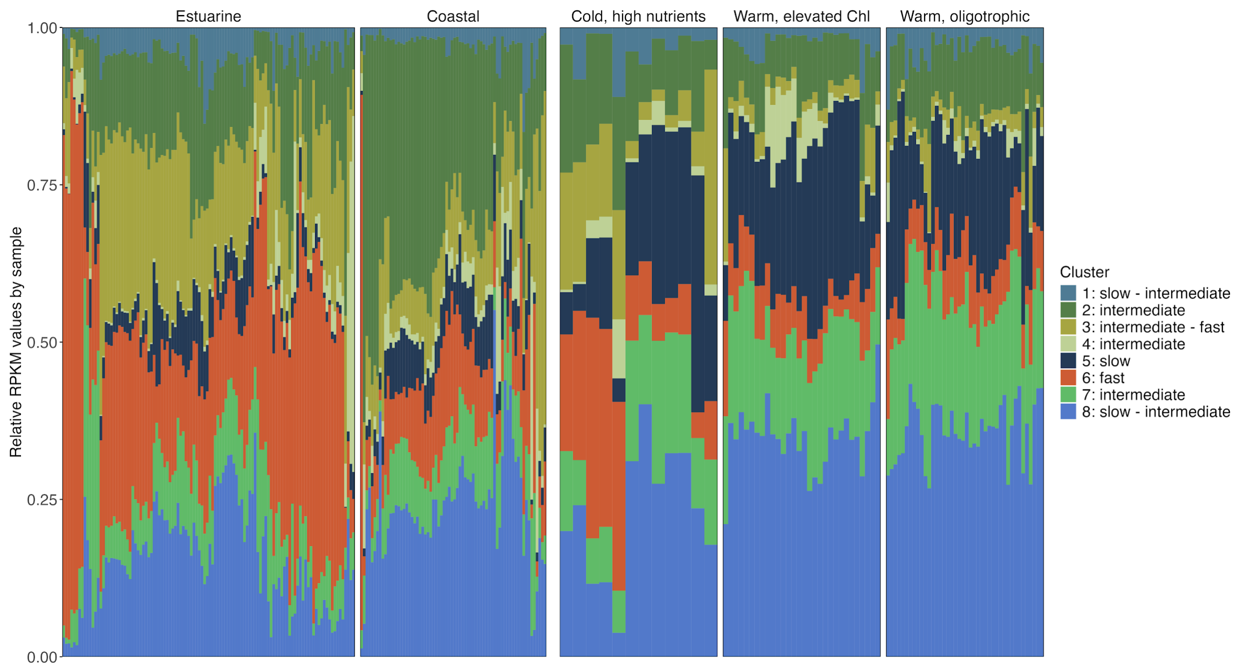

Supplemental Figure S17: Relative abundances by oceanographic regime/environmental ecotype. The 5 plot subpanels represent all the samples from just the Estuarine and Coastal oceanographic regimes, which did not have any representative environmental data along with the three environmental ecotypes defined on the Tara Oceans samples. Relative abundances are colored by the associated SOM cluster and color palette denotes each cluster's growth strategy with fast growers in red, intermediate growers in shades of green, and slow growers in shades of blue. Abundances are ordered within each of the 5 regimes according to a McQuitty linkage clustering on the Bray-Curtis similarity values of the cluster relative abundances.

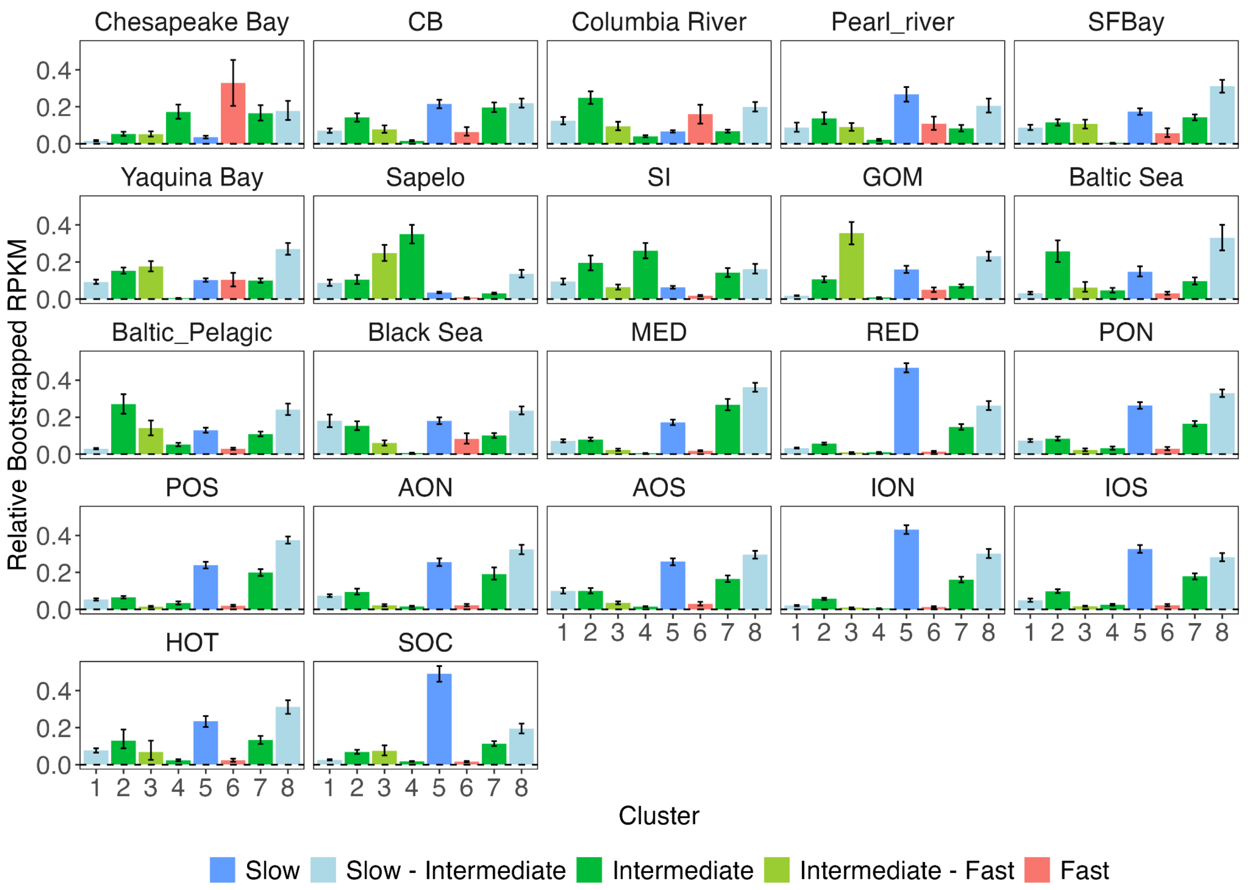

Supplemental Figure S18: Relative abundances of SOM clusters by oceanographic region. Sampling sites were grouped for bootstrapping according to the 22 oceanographic regions given in Table S4. For each region, bar plots of the average relative abundances of each of the 8 SOM clusters are shown. The relative abundances are calculated based on the bootstrap distributions of the raw RPKM values. The clusters are colored by their growth strategy (fast, fast-intermediate, slow-intermediate, and slow). Error bars represent the standard deviations of the bootstrapped distributions.

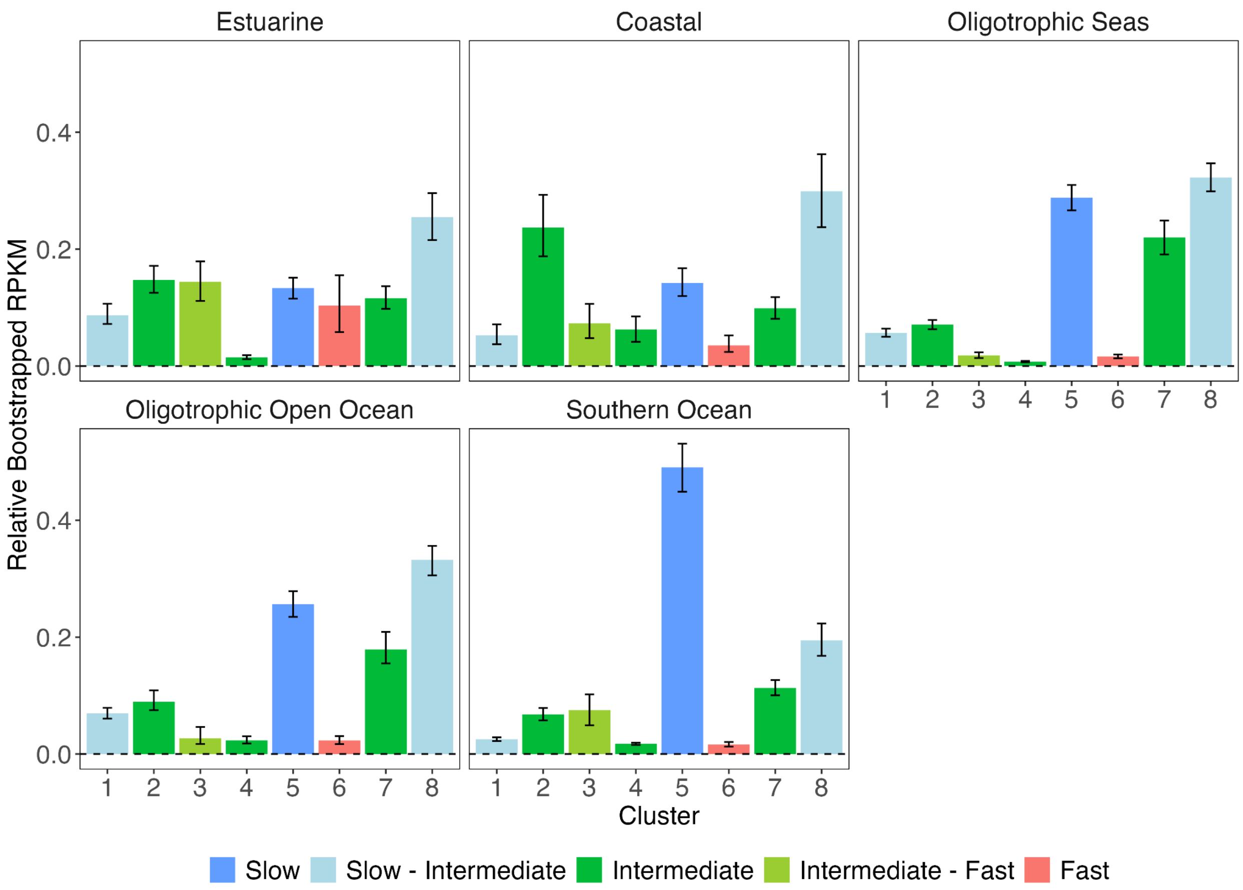

Supplemental Figure S19: Relative abundances of SOM clusters by oceanographic category. Sampling sites were grouped for bootstrapping according to the 5 oceanographic categories. Bar plots show the average relative abundances of each of the 8 SOM clusters in each category where the abundance is based on the bootstrap distributions of the raw RPKM values. The clusters are colored by their growth strategy (fast, fast-intermediate, slow-intermediate, and slow). Error bars represent the standard deviations of the bootstrapped distributions.

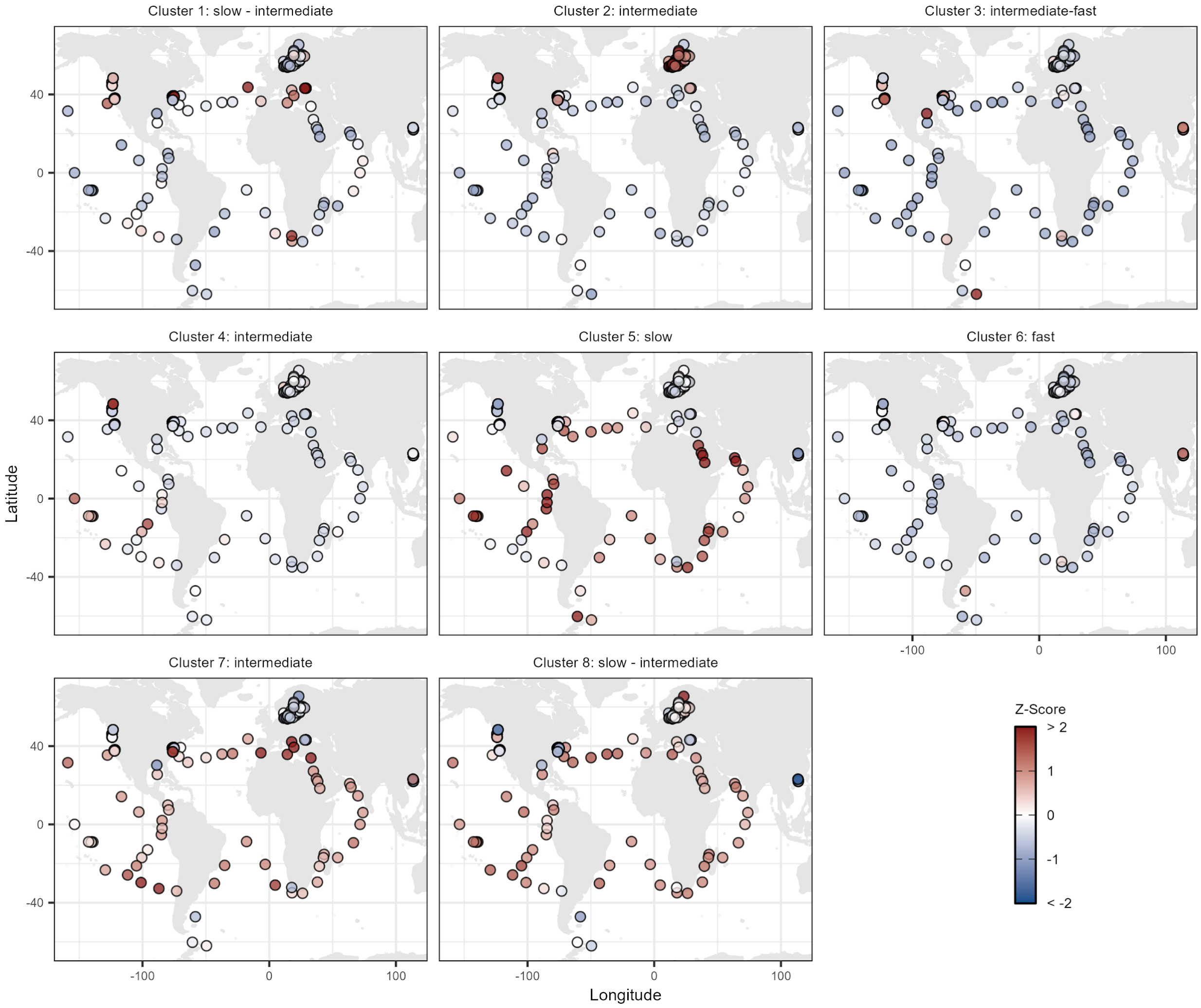

Supplemental Figure S20: Global map of cluster relative abundance. Each map of the globe shows the entire biogeography of stations surveyed by our read recruitment survey as individual circles. Each station is colored by the z-score value for a given SOM cluster from standardized relative abundance data with blue colors showing areas of rarity and red colors showing areas of enrichment for a given SOM cluster.

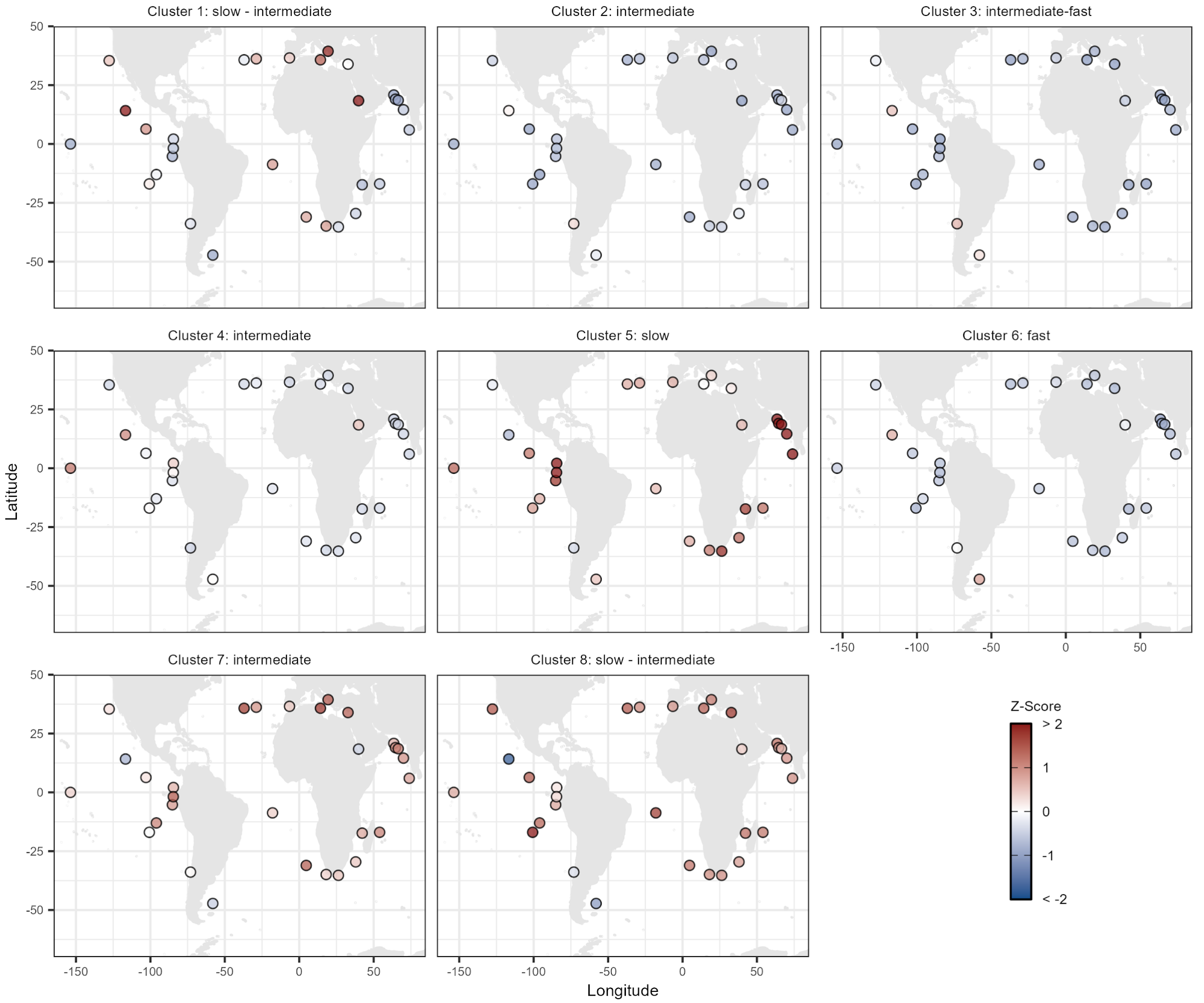

Supplemental Figure S21: Map of cluster relative abundances at Tara DCM samples. Each map of the globe shows the biogeography of Tara Oceans stations at the deep chlorophyll maximum (DCM) depth surveyed by our read recruitment survey as individual circles. Each station is colored by the z-score value for a given SOM cluster from standardized relative abundance data with blue colors showing areas of rarity and red colors showing areas of enrichment for a given SOM cluster.

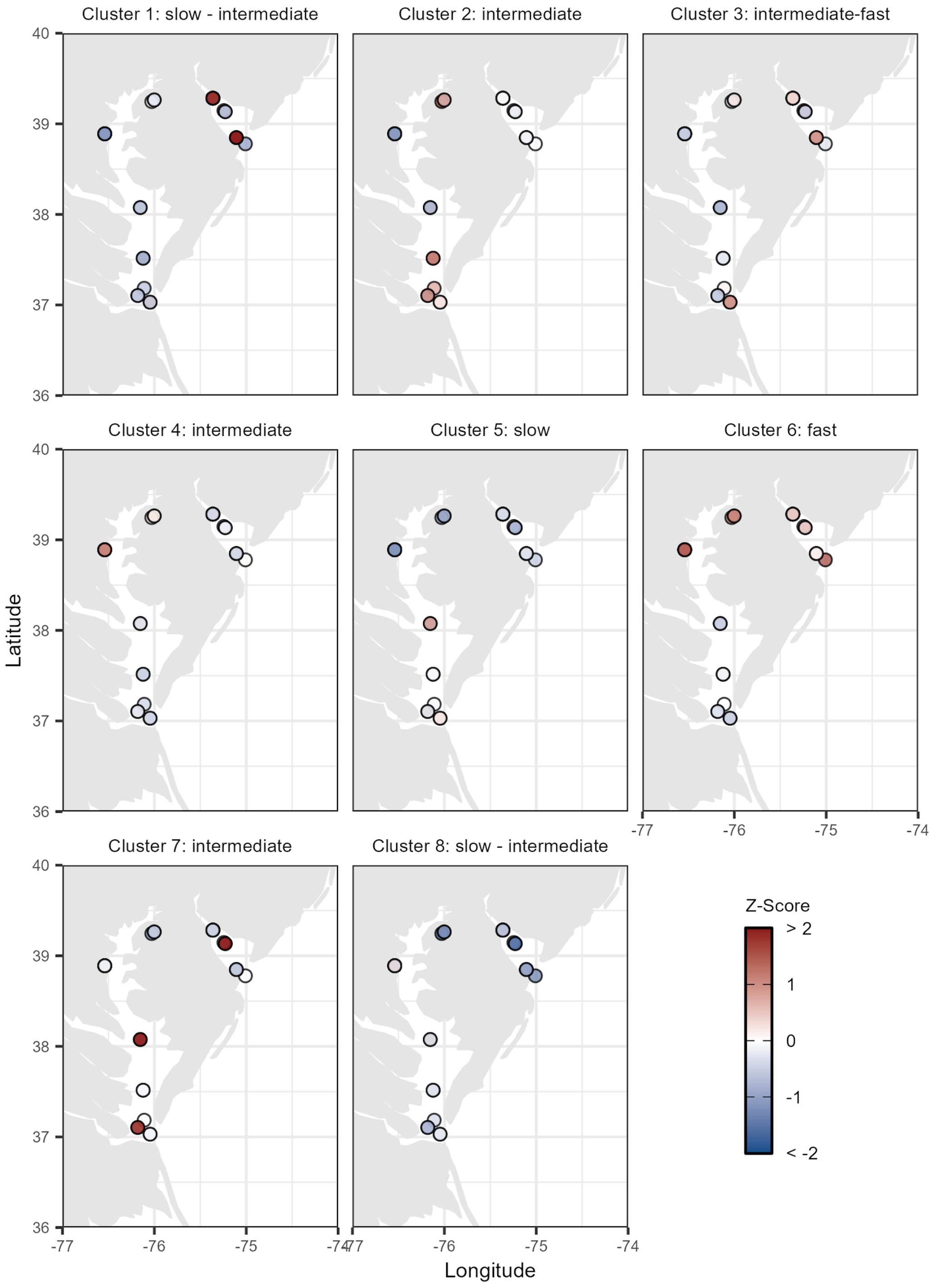

Supplemental Figure S22: Map of cluster relative abundances in Chesapeake Bay. Each map shows the biogeography of stations surveyed by our read recruitment survey, specifically from the Chesapeake Bay, as individual circles. Each station is colored by the z-score value for a given SOM cluster from standardized relative abundance data with blue colors showing areas of rarity and red colors showing areas of enrichment for a given SOM cluster.

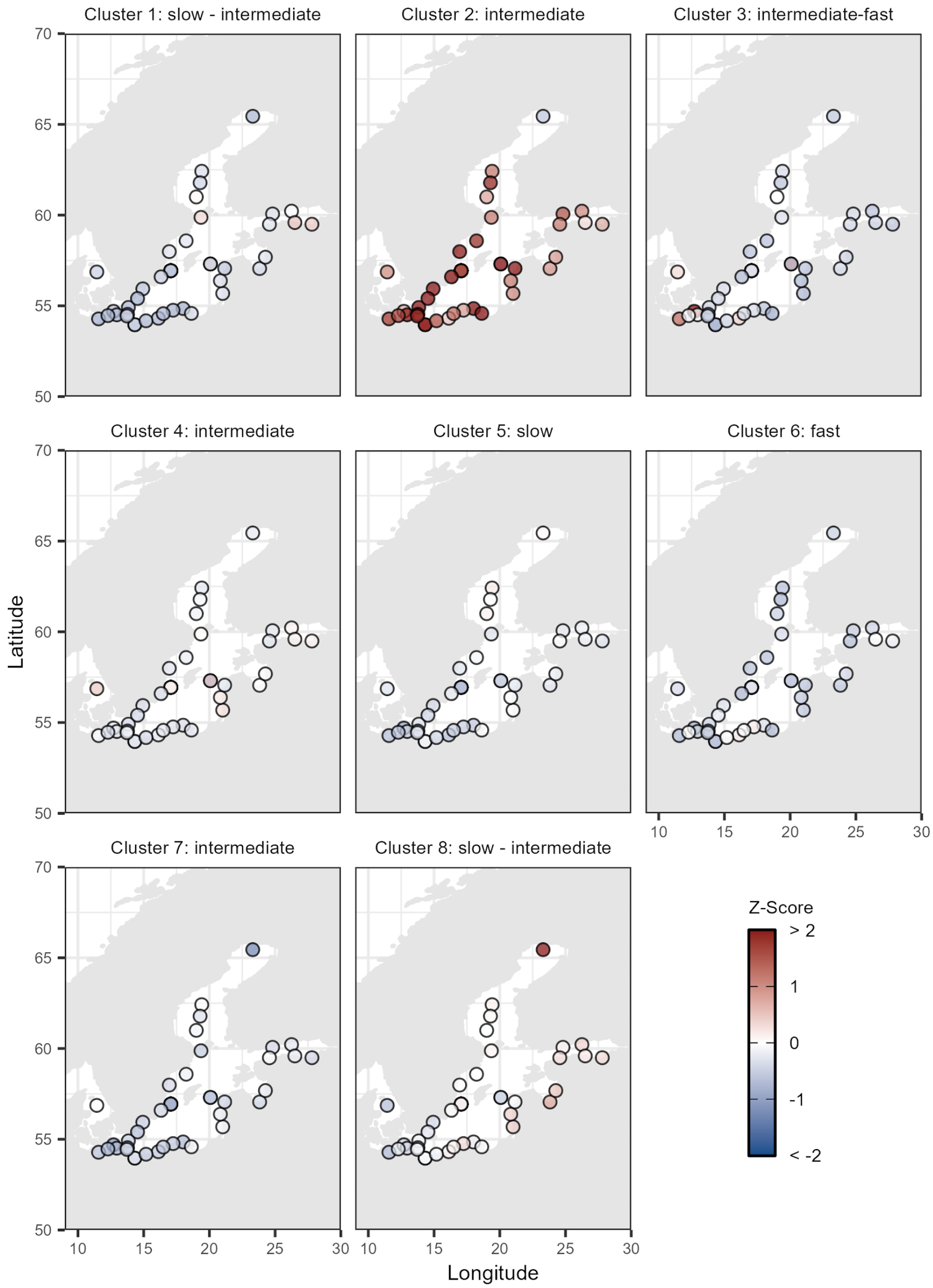

Supplemental Figure S23: Map of cluster relative abundances in the Baltic Sea. Each map shows the entire biogeography of stations surveyed by our read recruitment survey, specifically in the Baltic Sea, as individual circles. Each station is colored by the z-score value for a given SOM cluster from standardized relative abundance data with blue colors showing areas of rarity and red colors showing areas of enrichment for a given SOM cluster.

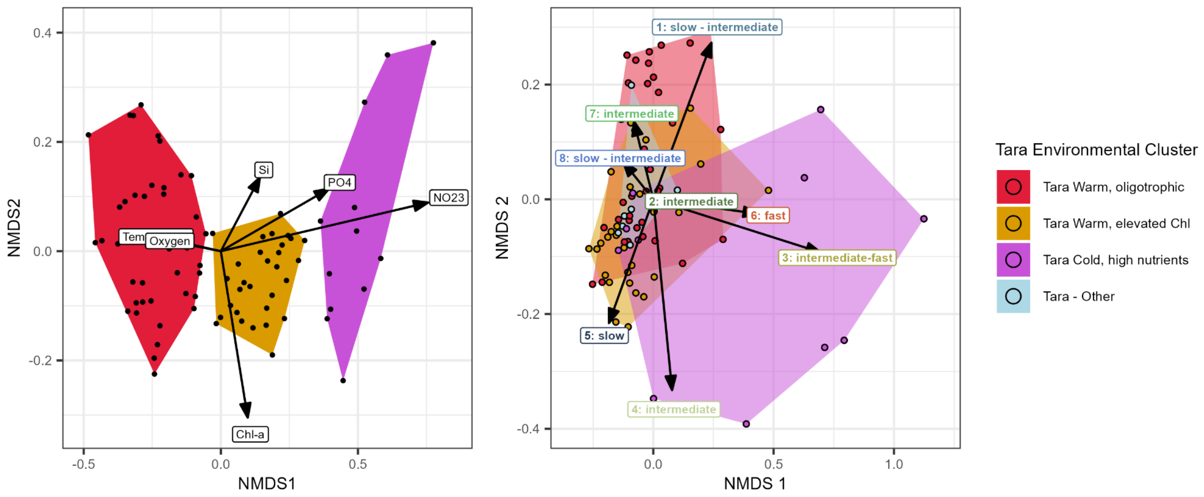

Supplemental Figure S24: Definition of environmental ecotypes. For the 84 samples from the Tara Oceans database in our metagenomic survey, we performed an NMDS of the environmental parameters (left panel) to identify distinct environmental ecotypes. This figure also shows the NMDS of cluster relative abundances colored by these three environmental ecotypes (right panel). Samples from Tara that could not be aligned to their corresponding environmental data are referred to as "Other". The ecotypes identified by the three distinct convex hulls (left panel) are carried over to the NMDS of cluster relative abundances (right panel) to demonstrate how these ecotypes relate to the relative abundances of the 8 SOM clusters. An additional hull is drawn around the samples that were labeled "Other" and their position suggests they likely are from water masses that are either oligotrophic or warm with elevated Chlorophyll.

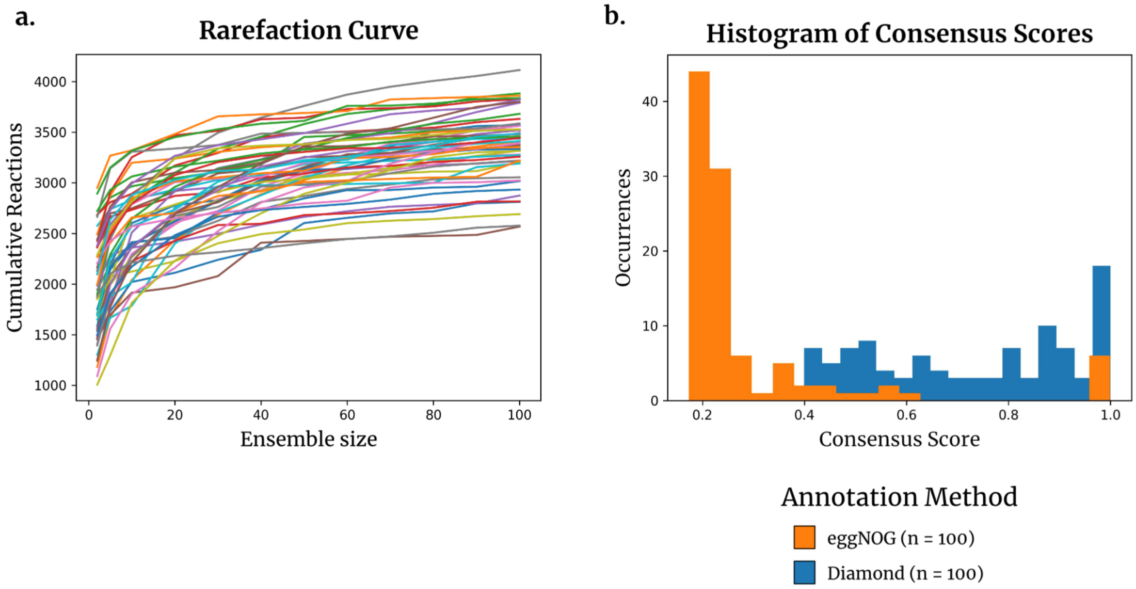

Supplemental Figure S25: CarveMe run parameterizations. (**A**) Rarefaction curve of the total number of unique reactions found in any model within an ensemble of models generated for a given genome. This curve was generated for ensemble sizes ranging from 2-100 models. At low ensemble sizes (e.g., in the range from 2-20) the model space rapidly identifies new unique reactions as more models are generated. The curves stabilize around ensemble sizes of 40-80 such that increasing the number of models in the ensemble does not add new reactions. (**B**) Histogram of the consensus scores for model ensembles when annotating reactions for CarveMe with eggNOG vs. the native Diamond (ensemble size = 60). Overall models generated with Diamond annotation produced significantly higher quality models than when eggNOG annotations were used for the same genomes.

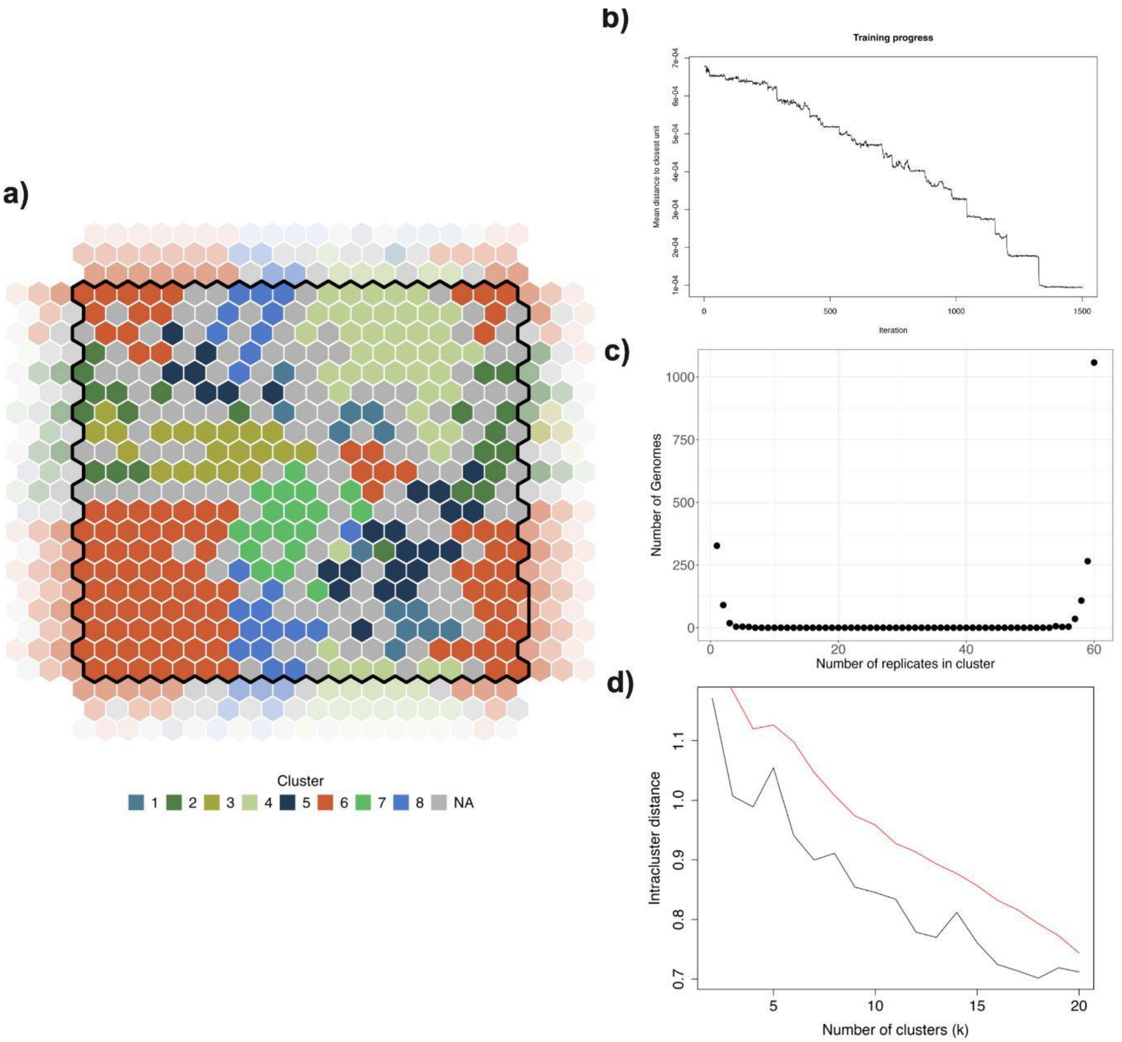

Supplemental Figure S26: SOM metrics. (**A**) The SOM grid is shown where each hexagon represents a grid point in the map (N=400). Grid points are colored by their assignment to the 8 defined SOM clusters. Grid points to which no genomes were assigned are colored gray to represent the absence of mapped data. It is important to note that the SOM uses a toroidal grid where the edges wrap around such that, for example, all nodes in Cluster 6 are in fact connected. We illustrate this wrapping with the transparent nodes shown outside the boundary of the SOM (denoted with the thick black line). (**B**) The training progress of the grid is shown for the duration of the map refinement process. (**C**) The number of models from each genome ensemble that were assigned to each SOM cluster, where a value of 60 denotes instances when all models from the ensemble were assigned to the same SOM cluster and a value of 0 denotes that no models from a specific ensemble were assigned to the cluster. 99.1% of genomes (1,465 of 1,478) have at least 57 models assigned to a single cluster (95% of models generated for the genome). 71.5% of the 1,478 genomes have all 60 models assigned to a single cluster. (**D**) Plot of intracluster distance for k-means and hierarchical clustering of the SOM map nodes. Intracluster distance is generally monotonically decreasing so cluster number is commonly determined based on field heuristics and inflection points in the intracluster distance function itself.

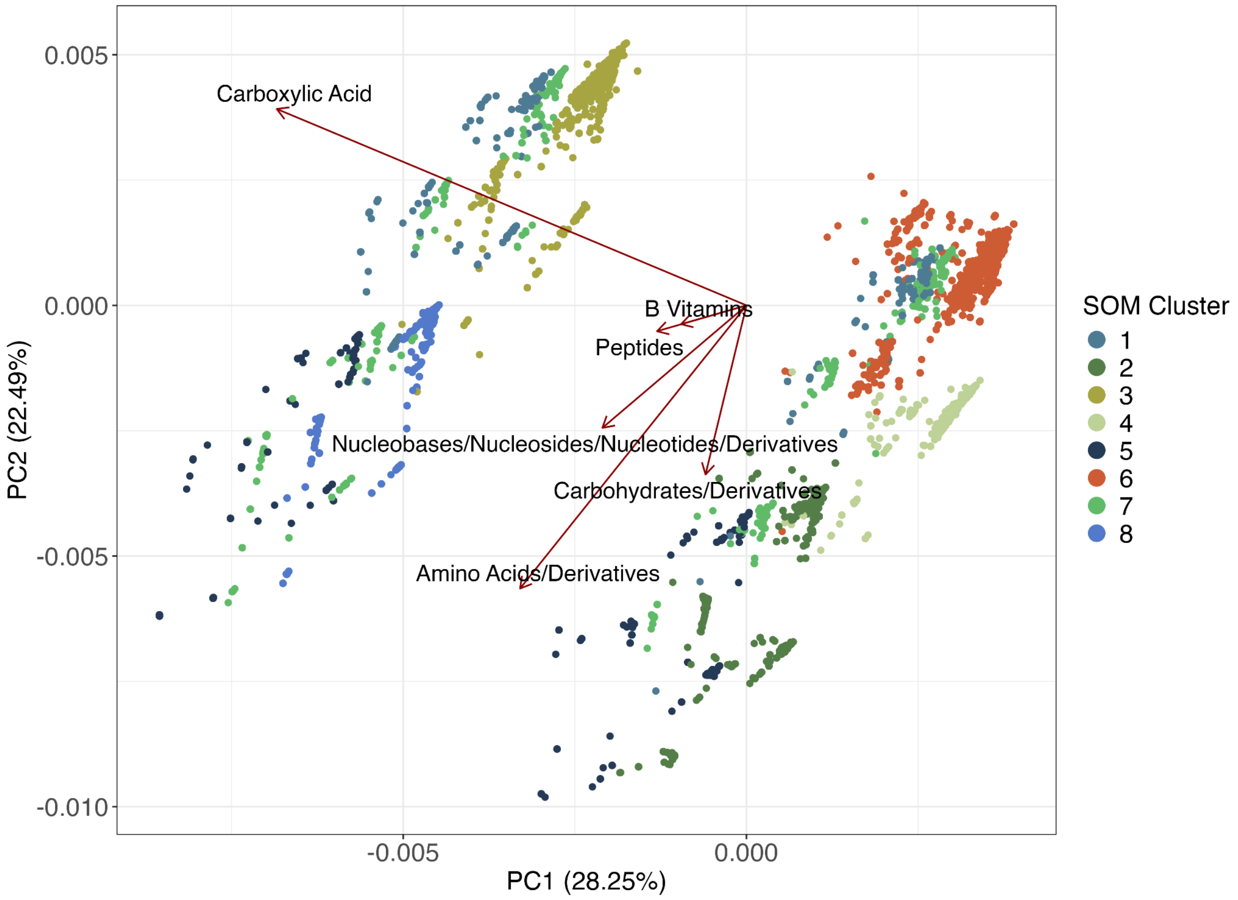

Supplemental Figure S27: PCA plot of the growth sensitivity data. The PCA captured 50.6% of the total variance on the first two principal component axes, and distinguished two major groups of data points, primarily separated by sensitivity to sugars (Carbohydrates/Derivatives) versus acids (Carboxylic Acids). Of note, the estimates of maximum growth rate were not included in this clustering. The points in the PCA are colored by SOM cluster assignment to illustrate that both approaches identified similar clustering of the data but that the SOM method differentiated the data into more distinct groups.

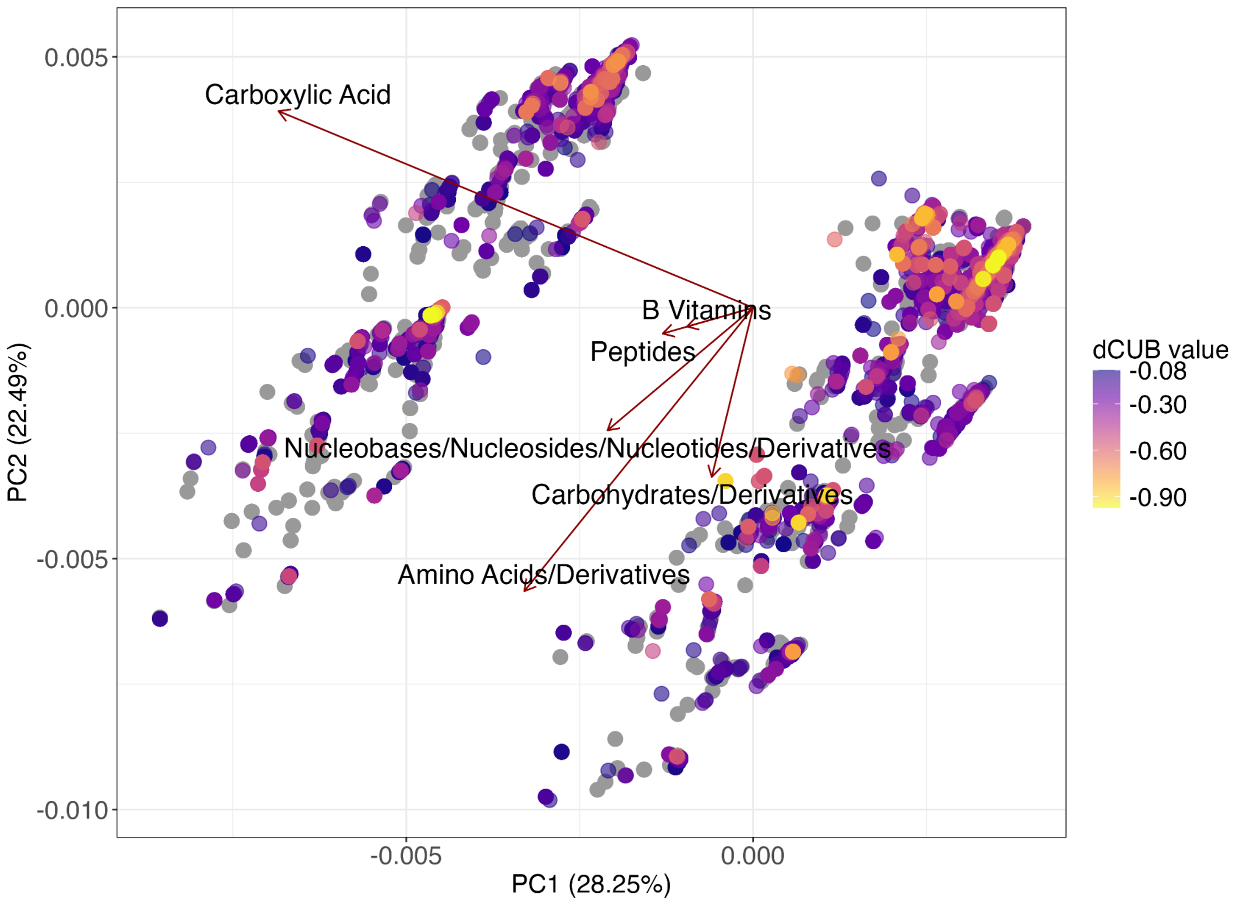

Supplemental Figure S28: PCA plot of the growth sensitivity data colored by dCUB. The PCA captured 50.6% of the total variance on the first two principal component axes, and distinguished two major groups of data points defined by the specific compound classes they're sensitive to. Of note, the estimates of maximum growth rate were not included in this clustering except as a post-processing variable. The points in the PCA are colored by this estimate of growth rate (dCUB) and highlights the greater density of fast-growing organisms (more yellow) anti-parallel to the axes of growth sensitivity.

Supplemental Table S1: All data for 1,478 high consensus genomes. This table provides the unique identifiers for the genomes used in the SOM analysis as well as information on the SOM cluster they were assigned to, their specific value of the dCUB growth proxy, taxonomic information (order, genus, and species), as well as the raw growth sensitivity values computed for the 11 compound classes clustered in this study.

Supplemental Table S2: Metabolite classification information. This table provides information on the 456 compounds that were manually classified for this study including their names in plain English, the compound class they were assigned to, and the name of the corresponding external reaction in the CarveMe universal model.

Supplemental Table S3: SRA accession numbers of surveyed metagenomes. This table provides a list of SRA accessions for the 1,209 surveyed metagenomes for our RPKM read recruitment analysis, as well as the read coverage of our samples in each metagenome.

Supplemental Table S4: Biogeographical distribution of SOM clusters by oceanographic region. This table provides the RPKM information for the 22 regions defined by Lanclos et al. (*74*) including the full name of each region, the identifier for each region, the oceanographic category each region was assigned to, the number of stations assigned to each region, and the relative abundance of the 8 SOM clusters based on the bootstrapped RPKM values.

Supplemental Table S5: Biogeographical distribution of SOM clusters by oceanographic category. This table provides the RPKM information for the 5 oceanographic categories defined in this study including the number of stations assigned to each category and the RPKM relative abundance information for each category.

Supplemental Table S6: Matrix of significance values for all pairs of SOM clusters based on dCUB distributions. This table provides the p-values for all paired comparisons of the distributions of growth rates (estimated using dCUB) for our 8 SOM clusters.

Supplemental Data S1: Relative abundance data of SOM clusters across six distinct ecotypes. This data file provides the relative abundance data used to construct the NMDS plot in main text Figure 3A, including all the relevant environmental metadata used to define the three ecotypes from Tara Oceans (Supplemental Figure S24).

REFERENCES

1. R. Reynolds, S. Hyun, B. Tully, J. Bien, N. M. Levine, Identification of microbial metabolic functional guilds from large genomic datasets. *Front. Microbiol.* **14**, 1197329 (2023).

2. J. Rivas-Santisteban, P. Yubero, S. Robaina-Estévez, J. M. González, J. Tamames, C. Pedrós-Alió, Quantifying microbial guilds. *ISME Commun.* **4**, ycae042 (2024).

3. S. Louca, L. W. Parfrey, M. Doebeli, Decoupling function and taxonomy in the global ocean microbiome. *Science* **353**, 1272–1277 (2016).

4. E. J. Zakem, J. McNichol, J. L. Weissman, Y. Raut, L. Xu, E. R. Halewood, C. A. Carlson, S. Dutkiewicz, J. A. Fuhrman, N. M. Levine, Predictable functional biogeography of marine microbial heterotrophs. bioRxiv 2024.02.14.580411 [Preprint] (2024); https://doi.org/10.1101/2024.02.14.580411.

5. S. T. Lennartz, D. P. Keller, A. Oschlies, B. Blasius, T. Dittmar, Mechanisms underpinning the net removal rates of dissolved organic carbon in the global ocean. *Global Biogeochem. Cycles* **38**, e2023GB007912 (2024).

6. A. L. Koch, Oligotrophs versus copiotrophs. *Bioessays* **23**, 657–661 (2001).

7. S. Liu, R. Parsons, K. Opalk, N. Baetge, S. Giovannoni, L. M. Bolaños, E. B. Kujawinski, K. Longnecker, Y. Lu, E. Halewood, C. A. Carlson, Different carboxyl-rich alicyclic molecules proxy compounds select distinct bacterioplankton for oxidation of dissolved organic matter in the mesopelagic Sargasso Sea. *Limnol. Oceanogr.* **65**, 1532–1553 (2020).

8. M. A. Oberhardt, B. Ø. Palsson, J. A. Papin, Applications of genome-scale metabolic reconstructions. *Mol. Syst. Biol.* **5**, 320 (2009).

9. C. Gu, G. B. Kim, W. J. Kim, H. U. Kim, S. Y. Lee, Current status and applications of genome-scale metabolic models. *Genome Biol.* **20**, 1–18 (2019).

10. D. Machado, S. Andrejev, M. Tramontano, K. R. Patil, Fast automated reconstruction of genome-scale metabolic models for microbial species and communities. *Nucleic Acids Res.* **46**, 7542–7553 (2018).

11. C. S. Henry, M. DeJongh, A. A. Best, P. M. Frybarger, B. Linsay, R. L. Stevens, High-throughput generation, optimization and analysis of genome-scale metabolic models. *Nat. Biotechnol.* **28**, 977–982 (2010).

12. S. Magnúsdóttir, A. Heinken, L. Kutt, D. A. Ravcheev, E. Bauer, A. Noronha, K. Greenhalgh, C. Jäger, J. Baginska, P. Wilmes, R. M. T. Fleming, I. Thiele, Generation of genome-scale metabolic reconstructions for 773 members of the human gut microbiota. *Nat. Biotechnol.* **35**, 81–89 (2017).

13. S. N. Mendoza, B. G. Olivier, D. Molenaar, B. Teusink, A systematic assessment of current genome-scale metabolic reconstruction tools. *Genome Biol.* **20**, 158 (2019).

14. A. Régimbeau, M. Budinich, A. Larhlimi, J. J. Pierella Karlusich, O. Aumont, L. Memery, C. Bowler, D. Eveillard, Contribution of genome-scale metabolic modelling to niche theory. *Ecol. Lett.* **25**, 1352–1364 (2022).

15. L. Paoli, H.-J. Ruscheweyh, C. C. Forneris, F. Hubrich, S. Kautsar, A. Bhushan, A. Lotti, Q. Clayssen, G. Salazar, A. Milanese, C. I. Carlström, C. Papadopoulou, D. Gehrig, M. Karasikov, H. Mustafa, M. Larralde, L. M. Carroll, P. Sánchez, A. A. Zayed, D. R. Cronin, S. G. Acinas, P. Bork, C. Bowler, T. O. Delmont, J. M. Gasol, A. D. Gossert, A. Kahles, M. B. Sullivan, P. Wincker, G. Zeller, S. L. Robinson, J. Piel, S. Sunagawa, Biosynthetic potential of the global ocean microbiome. *Nature* **607**, 111–118 (2022).

16. D. B. Bernstein, S. Sulheim, E. Almaas, D. Segrè, Addressing uncertainty in genome-scale metabolic model reconstruction and analysis. *Genome Biol.* **22**, 64 (2021).

17. N. Giordano, M. Gaudin, C. Trottier, E. Delage, C. Nef, C. Bowler, S. Chaffron, Genome-scale community modelling reveals conserved metabolic cross-feedings in epipelagic bacterioplankton communities. *Nat. Commun.* **15**, 2721 (2024).

18. M. Gralka, S. Pollak, O. X. Cordero, Genome content predicts the carbon catabolic preferences of heterotrophic bacteria. *Nat. Microbiol.* **8**, 1799–1808 (2023).

19. S. J. Giovannoni, SAR11 bacteria: The most abundant plankton in the oceans. *Ann. Rev. Mar. Sci.* **9**, 231–255 (2017).

20. B. K. Swan, B. Tupper, A. Sczyrba, F. M. Lauro, M. Martinez-Garcia, J. M. González, H. Luo, J. J. Wright, Z. C. Landry, N. W. Hanson, B. P. Thompson, N. J. Poulton, P. Schwientek, S. G. Acinas, S. J. Giovannoni, M. A. Moran, S. J. Hallam, R. Cavicchioli, T. Woyke, R. Stepanauskas, Prevalent genome streamlining and latitudinal divergence of planktonic bacteria in the surface ocean. *Proc. Natl. Acad. Sci. U.S.A.* **110**, 11463–11468 (2013).

21. E. W. Getz, V. C. Lanclos, C. Y. Kojima, C. Cheng, M. W. Henson, M. E. Schön, T. J. G. Ettema, B. C. Faircloth, J. C. Thrash, The AEGEAN-169 clade of bacterioplankton is synonymous with SAR11 subclade V (HIMB59) and metabolically distinct. *mSystems* **8**, e0017923 (2023).

22. R. Wehrens, L. M. C. Buydens, Self- and super-organizing maps in R: The kohonen package. *J. Stat. Softw.* **21**, 1–19 (2007).

23. T. Kohonen, The self-organizing map. *Proc. IEEE* **78**, 1464–1480 (1990).

24. J. L. Weissman, S. Hou, J. A. Fuhrman, Estimating maximal microbial growth rates from cultures, metagenomes, and single cells via codon usage patterns. *Proc. Natl. Acad. Sci. U.S.A.* **118**, e2016810118 (2021).

25. J. L. Weissman, E.-R. O. Dimbo, A. I. Krinos, C. Neely, Y. Yagües, D. Nolin, S. Hou, S. Laperriere, D. A. Caron, B. Tully, H. Alexander, J. A. Fuhrman, Estimating global variation in the maximum growth rates of eukaryotic microbes from cultures and metagenomes via codon usage patterns. bioRxiv 2021.10.15.464604 [Preprint] (2022); https://doi.org/10.1101/2021.10.15.464604.

26. K. G. Lloyd, A. D. Steen, J. Ladau, J. Yin, L. Crosby, Phylogenetically novel uncultured microbial cells dominate Earth microbiomes. *mSystems* **3**, 10.1128/msystems.00055-18 (2018).

27. O. Deulofeu-Capo, M. Sebastián, A. Auladell, C. Cardelús, I. Ferrera, O. Sánchez, J. M. Gasol, Growth rates of marine prokaryotes are extremely diverse, even among closely related taxa. *ISME Commun.* **4**, ycae066 (2024).

28. G. Salazar, L. Paoli, A. Alberti, J. Huerta-Cepas, H.-J. Ruscheweyh, M. Cuenca, C. M. Field, L. P. Coelho, C. Cruaud, S. Engelen, A. C. Gregory, K. Labadie, C. Marec, E. Pelletier, M. Royo-Llonch, S. Roux, P. Sánchez, H. Uehara, A. A. Zayed, G. Zeller, M. Carmichael, C. Dimier, J. Ferland, S. Kandels, M. Picheral, S. Pisarev, J. Poulain, Tara Oceans Coordinators, S. G. Acinas, M. Babin, P. Bork, C. Bowler, C. de Vargas, L. Guidi, P. Hingamp, D. Iudicone, L. Karp-Boss, E. Karsenti, H. Ogata, S. Pesant, S. Speich, M. B. Sullivan, P. Wincker, S. Sunagawa, Gene expression changes and community turnover differentially shape the global ocean metatranscriptome. *Cell* **179**, 1068–1083.e21 (2019).

29. Z. Zhang, Q. Zhang, B. Chen, Y. Yu, T. Wang, N. Xu, X. Fan, J. Penuelas, Z. Fu, Y. Deng, Y.-G. Zhu, H. Qian, Global biogeography of microbes driving ocean ecological status under climate change. *Nat. Commun.* **15**, 4657 (2024).

30. S. Sunagawa, L. P. Coelho, S. Chaffron, J. R. Kultima, K. Labadie, G. Salazar, B. Djahanschiri, G. Zeller, D. R. Mende, A. Alberti, F. M. Cornejo-Castillo, P. I. Costea, C. Cruaud, F. d'Ovidio, S. Engelen, I. Ferrera, J. M. Gasol, L. Guidi, F. Hildebrand, F. Kokoszka, C. Lepoivre, G. Lima-Mendez, J. Poulain, B. T. Poulos, M. Royo-Llonch, H. Sarmento, S. Vieira-Silva, C. Dimier, M. Picheral, S. Searson, S. Kandels-Lewis, Tara Oceans Coordinators, C. Bowler, C. de Vargas, G. Gorsky, N. Grimsley, P. Hingamp, D. Iudicone, O. Jaillon, F. Not, H. Ogata, S. Pesant, S. Speich, L. Stemmann, M. B. Sullivan, J. Weissenbach, P. Wincker, E. Karsenti, J. Raes, S. G. Acinas, P. Bork, Structure and function of the global ocean microbiome. *Science* **348**, 1261359 (2015).

31. L. P. Coelho, R. Alves, Á. R. Del Río, P. N. Myers, C. P. Cantalapiedra, J. Giner-Lamia, T. S. Schmidt, D. R. Mende, A. Orakov, I. Letunic, F. Hildebrand, T. Van Rossum, S. K. Forslund, S. Khedkar, O. M. Maistrenko, S. Pan, L. Jia, P. Ferretti, S. Sunagawa, X.-M. Zhao, H. B. Nielsen, J. Huerta-Cepas, P. Bork, Towards the biogeography of prokaryotic genes. *Nature* **601**, 252–256 (2022).

32. L. Guidi, S. Chaffron, L. Bittner, D. Eveillard, A. Larhlimi, S. Roux, Y. Darzi, S. Audic, L. Berline, J. Brum, L. P. Coelho, J. C. I. Espinoza, S. Malviya, S. Sunagawa, C. Dimier, S. Kandels-Lewis, M. Picheral, J. Poulain, S. Searson, Tara Oceans coordinators, L. Stemmann, F. Not, P. Hingamp, S. Speich, M. Follows, L. Karp-Boss, E. Boss, H. Ogata, S. Pesant, J. Weissenbach, P. Wincker, S. G. Acinas, P. Bork, C. de Vargas, D. Iudicone, M. B. Sullivan, J. Raes, E. Karsenti, C. Bowler, G. Gorsky, Plankton networks driving carbon export in the oligotrophic ocean. *Nature* **532**, 465–470 (2016).

33. Q. Peng, C. Zhao, X. Wang, K. Cheng, C. Wang, X. Xu, L. Lin, Modeling bacterial interactions uncovers the importance of outliers in the coastal lignin-degrading consortium. *Nat. Commun.* **16**, 639 (2025).

34. J. Jing, P. Garbeva, J. M. Raaijmakers, M. H. Medema, Strategies for tailoring functional microbial synthetic communities. *ISME J.* **18**, wrae049 (2024).

35. Z. Ruan, K. Chen, W. Cao, L. Meng, B. Yang, M. Xu, Y. Xing, P. Li, S. Freilich, C. Chen, Y. Gao, J. Jiang, X. Xu, Engineering natural microbiomes toward enhanced bioremediation by microbiome modeling. *Nat. Commun.* **15**, 4694 (2024).

36. M. Saito, W. H. O. Institution, H. Alexander, H. Benway, P. Boyd, M. Gledhill, E. Kujawinski, N. Levine, M. Maheigan, A. Marchetti, I. Obernosterer, A. Santoro, D. Shi, K. Suzuki, A. Tagliabue, B. Twining, M. Maldonado, The dawn of the BioGeoSCAPES program: Ocean metabolism and nutrient cycles on a changing planet. *Oceanography* **37**, 162–166 (2024).

37. S. Clayton, H. Alexander, J. R. Graff, N. J. Poulton, L. R. Thompson, H. Benway, E. Boss, A. Martiny, Bio-GO-SHIP: The time is right to establish global repeat sections of ocean biology. *Front. Mar. Sci.* **8**, 767443 (2022).

38. AtlantECO Consortium, AtlantECO—Atlantic ecosystems assessment, forecasting and sustainability, Zenodo (2020); https://doi.org/10.5281/ZENODO.3725912.

39. D. H. Parks, C. Rinke, M. Chuvochina, P.-A. Chaumeil, B. J. Woodcroft, P. N. Evans, P. Hugenholtz, G. W. Tyson, Recovery of nearly 8,000 metagenome-assembled genomes substantially expands the tree of life. *Nat. Microbiol.* **2**, 1533–1542 (2017).

40. B. J. Tully, E. D. Graham, J. F. Heidelberg, The reconstruction of 2,631 draft metagenome-assembled genomes from the global oceans. *Sci. Data* **5**, 170203 (2018).

41. D. H. Parks, M. Imelfort, C. T. Skennerton, P. Hugenholtz, G. W. Tyson, CheckM: Assessing the quality of microbial genomes recovered from isolates, single cells, and metagenomes. *Genome Res.* **25**, 1043–1055 (2015).

42. A. M. Eren, Ö. C. Esen, C. Quince, J. H. Vineis, H. G. Morrison, M. L. Sogin, T. O. Delmont, Anvi'o: An advanced analysis and visualization platform for 'omics data. *PeerJ* **3**, e1319 (2015).

43. M. R. Olm, C. T. Brown, B. Brooks, J. F. Banfield, dRep: A tool for fast and accurate genomic comparisons that enables improved genome recovery from metagenomes through de-replication. *ISME J.* **11**, 2864–2868 (2017).

44. M. D. Lee, GToTree: A user-friendly workflow for phylogenomics. *Bioinformatics* **35**, 4162–4164 (2019).

45. B. Q. Minh, H. A. Schmidt, O. Chernomor, D. Schrempf, M. D. Woodhams, A. von Haeseler, R. Lanfear, IQ-TREE 2: New models and efficient methods for phylogenetic inference in the genomic era. *Mol. Biol. Evol.* **37**, 1530–1534 (2020).

46. Z. A. King, J. Lu, A. Dräger, P. Miller, S. Federowicz, J. A. Lerman, A. Ebrahim, B. O. Palsson, N. E. Lewis, BiGG models: A platform for integrating, standardizing and sharing genome-scale models. *Nucleic Acids Res.* **44**, D515–22 (2016).

47. P.-A. Chaumeil, A. J. Mussig, P. Hugenholtz, D. H. Parks, GTDB-Tk v2: Memory friendly classification with the genome taxonomy database. *Bioinformatics* **38**, 5315–5316 (2022).

48. D. H. Parks, M. Chuvochina, C. Rinke, A. J. Mussig, P.-A. Chaumeil, P. Hugenholtz, GTDB: An ongoing census of bacterial and archaeal diversity through a phylogenetically consistent, rank normalized and complete genome-based taxonomy. *Nucleic Acids Res.* **50**, D785–D794 (2022).

49. G. Van Rossum, F. L. Drake, *Python 3 Reference Manual: (Python Documentation Manual Part 2)* (CreateSpace Independent Publishing Platform, 2009).

50. B. Buchfink, C. Xie, D. H. Huson, Fast and sensitive protein alignment using DIAMOND. *Nat. Methods* **12**, 59–60 (2015).

51. J. Hastings, G. Owen, A. Dekker, M. Ennis, N. Kale, V. Muthukrishnan, S. Turner, N. Swainston, P. Mendes, C. Steinbeck, ChEBI in 2016: Improved services and an expanding collection of metabolites. *Nucleic Acids Res.* **44**, D1214-9 (2016).

52. S. Kim, J. Chen, T. Cheng, A. Gindulyte, J. He, S. He, Q. Li, B. A. Shoemaker, P. A. Thiessen, B. Yu, L. Zaslavsky, J. Zhang, E. E. Bolton, PubChem 2023 update. *Nucleic Acids Res.* **51**, D1373–D1380 (2023).

53. J. Schellenberger, J. O. Park, T. M. Conrad, B. Ø. Palsson, BiGG: A biochemical genetic and genomic knowledgebase of large scale metabolic reconstructions. *BMC Bioinformatics* **11**, 213 (2010).

54. C. J. Norsigian, N. Pusarla, J. L. McConn, J. T. Yurkovich, A. Dräger, B. O. Palsson, Z. King, BiGG models 2020: Multi-strain genome-scale models and expansion across the phylogenetic tree. *Nucleic Acids Res.* **48**, D402–D406 (2020).

55. D. S. Wishart, A. Guo, E. Oler, F. Wang, A. Anjum, H. Peters, R. Dizon, Z. Sayeeda, S. Tian, B. L. Lee, M. Berjanskii, R. Mah, M. Yamamoto, J. Jovel, C. Torres-Calzada, M. Hiebert-Giesbrecht, V. W. Lui, D. Varshavi, D. Varshavi, D. Allen, D. Arndt, N. Khetarpal, A. Sivakumaran, K. Harford, S. Sanford, K. Yee, X. Cao, Z. Budinski, J. Liigand, L. Zhang, J. Zheng, R. Mandal, N. Karu, M. Dambrova, H. B. Schiöth, R. Greiner, V. Gautam, HMDB 5.0: The human metabolome database for 2022. *Nucleic Acids Res.* **50**, D622–D631 (2022).

56. P. D. Karp, R. Billington, R. Caspi, C. A. Fulcher, M. Latendresse, A. Kothari, I. M. Keseler, M. Krummenacker, P. E. Midford, Q. Ong, W. K. Ong, S. M. Paley, P. Subhraveti, The BioCyc collection of microbial genomes and metabolic pathways. *Brief. Bioinform.* **20**, 1085–1093 (2019).

57. R. Caspi, R. Billington, I. M. Keseler, A. Kothari, M. Krummenacker, P. E. Midford, W. K. Ong, S. Paley, P. Subhraveti, P. D. Karp, The MetaCyc database of metabolic pathways and enzymes—A 2019 update. *Nucleic Acids Res.* **48**, D445–D453 (2020).

58. H. E. Pence, A. Williams, ChemSpider: An online chemical information resource. *J. Chem. Educ.* **87**, 1123–1124 (2010).

59. A. C. Guo, T. Jewison, M. Wilson, Y. Liu, C. Knox, Y. Djoumbou, P. Lo, R. Mandal, R. Krishnamurthy, D. S. Wishart, ECMDB: The *E. coli* metabolome database. *Nucleic Acids Res.* **41**, D625–30 (2012).

60. T. Sajed, A. Marcu, M. Ramirez, A. Pon, A. C. Guo, C. Knox, M. Wilson, J. R. Grant, Y. Djoumbou, D. S. Wishart, ECMDB 2.0: A richer resource for understanding the biochemistry of E. coli. *Nucleic Acids Res.* **44**, D495–501 (2016).

61. A. Ebrahim, J. A. Lerman, B. O. Palsson, D. R. Hyduke, COBRApy: COnstraints-based reconstruction and analysis for Python. *BMC Syst. Biol.* **7**, 74 (2013).

62. A. R. Wilcock, D. M. Goldberg, Kinetic determination of malate dehydrogenase activity eliminating problems due to spontaneous conversion of oxaloacetate to pyruvate. *Biochem. Med.* **6**, 116–126 (1972).

63. Y.-S. Park, R. Céréghino, A. Compin, S. Lek, Applications of artificial neural networks for patterning and predicting aquatic insect species richness in running waters. *Ecol. Model.* **160**, 265–280 (2003).

64. R. Céréghino, Y.-S. Park, Review of the self-organizing map (SOM) approach in water resources: Commentary. *Environ. Model. Software* **24**, 945–947 (2009).

65. A. M. Kalteh, P. Hjorth, R. Berndtsson, Review of the self-organizing map (SOM) approach in water resources: Analysis, modelling and application. *Environ. Model. Software* **23**, 835–845 (2008).

66. K. Kiviluoto, "Topology preservation in self-organizing maps" in *Proceedings of International Conference on Neural Networks (ICNN'96)* (IEEE, 1996), vol. 1, pp. 294–299.

67. J. A. Hartigan, M. A. Wong, Algorithm AS 136: A *K*-means clustering algorithm. *J. R. Stat. Soc. Ser. C Appl. Stat.* **28**, 100–108 (1979).

68. L. Hubert, P. Arabie, Comparing partitions. *J. Classif.* **2**, 193–218 (1985).

69. C. Y. Kojima, E. W. Getz, J. C. Thrash, RRAP: RPKM recruitment analysis pipeline. *Microbiol. Resour. Announc.* **11**, e0064422 (2022).

70. B. Langmead, S. L. Salzberg, Fast gapped-read alignment with Bowtie 2. *Nat. Methods* **9**, 357–359 (2012).

71. P. Danecek, J. K. Bonfield, J. Liddle, J. Marshall, V. Ohan, M. O. Pollard, A. Whitwham, T. Keane, S. A. McCarthy, R. M. Davies, H. Li, Twelve years of SAMtools and BCFtools. *Gigascience* **10**, giab008 (2021).

72. Tara Oceans Consortium, Coordinators, Tara Oceans Expedition, Participants, Registry of all samples from the Tara Oceans Expedition (2009–2013). *PANGAEA*, 10.1594/PANGAEA.875582 (2017).

73. A. E. Minoche, J. C. Dohm, H. Himmelbauer, Evaluation of genomic high-throughput sequencing data generated on Illumina HiSeq and genome analyzer systems. *Genome Biol.* **12**, R112 (2011).

74. V. C. Lanclos, A. N. Rasmussen, C. Y. Kojima, C. Cheng, M. W. Henson, B. C. Faircloth, C. A. Francis, J. C. Thrash, Ecophysiology and genomics of the brackish water adapted SAR11 subclade IIIa. *ISME J.* **17**, 620–629 (2023).

75. J. McNichol, N. L. R. Williams, Y. Raut, C. Carlson, E. R. Halewood, K. Turk-Kubo, J. P. Zehr, A. P. Rees, G. Tarran, M. R. Gradoville, M. Wietz, C. Bienhold, K. Metfies, S. Torres-Valdés, T. Mock, S. L. Eggers, W. Jeffrey, J. Moss, P. Berube, S. Biller, L. Bodrossy, J. Van De Kamp, M. Brown, S. L. S. Sow, E. V. Armbrust, J. Fuhrman, Characterizing organisms from three domains of life with universal primers from throughout the global ocean. *Sci. Data* **12**, 1078 (2025).

76. T. Seemann, Barrnap: Microscope: Bacterial ribosomal RNA predictor, Github; https://github.com/tseemann/barrnap.

77. C. O. Wilke, ggridges: Ridgeline plots in "ggplot2" (2024); https://wilkelab.org/ggridges/.

78. G. Yu, D. K. Smith, H. Zhu, Y. Guan, T. T.-Y. Lam, Ggtree: An R package for visualization and annotation of phylogenetic trees with their covariates and other associated data. *Methods Ecol. Evol.* **8**, 28–36 (2017).

79. T. L. Pedersen, patchwork: The composer of plots (2024); https://patchwork.data-imaginist.com.

80. J. B. Russell, F. Diez-Gonzalez, The effects of fermentation acids on bacterial growth. *Adv. Microb. Physiol.* **39**, 205–234 (1998).

81. J. Vesanto, "Neural network tool for data mining: SOM toolbox" (Helsinki University of Technology, 2000); https://citeseerx.ist.psu.edu/document?repid=rep1&type=pdf&doi=0e9bee375e885c4740ba0dab007167c485fa1e48.

82. T. Kohonen, The self-organizing map. *Neurocomputing* **21**, 1–6 (1998).

83. S. M. Gifford, S. Sharma, M. Booth, M. A. Moran, Expression patterns reveal niche diversification in a marine microbial assemblage. *ISME J.* **7**, 281–298 (2013).

84. H. Sarmento, C. Morana, J. M. Gasol, Bacterioplankton niche partitioning in the use of phytoplankton-derived dissolved organic carbon: quantity is more important than quality. *ISME J.* **10**, 2582–2592 (2016).
